# Pragmatic methods for reviewing exceptionally large bodies of evidence: systematic mapping review and overview of systematic reviews using lung cancer survival as an exemplar

**DOI:** 10.1186/s13643-019-1087-4

**Published:** 2019-07-16

**Authors:** Ruth Lewis, Maggie Hendry, Nafees Din, Marian A. Stanciu, Sadia Nafees, Annie Hendry, Zhi Hao Teoh, Thomas Lloyd, Rachel Parsonage, Richard D. Neal, Gareth Collier, Dyfed W. Huws

**Affiliations:** 1 North Wales Centre for Primary Care Research, School of Health Sciences, Bangor University, Cambrian 2, Wrexham Technology Park, Wrexham, LL13 7YP UK; 2 Academic Unit of Primary Care, Leeds Institute of Health Sciences, University of Leeds, Leeds, UK; 3 Hywel Dda University Health Board, Carmarthen, Wales, UK; 4 Welsh Cancer Intelligence and Surveillance Unit (WCISU), Health Intelligence Division, Public Health Wales, Cardiff, UK; 5 Swansea University, Swansea, UK; 6 Centre for Mental Health and Society, School of Health Sciences, Bangor University, Academic Unit, Wrexham Technology Park, Wrexham, LL13 7YP UK

**Keywords:** Lung cancer, Prognostic factors, Prognostic research, Overview of reviews, Systematic review

## Abstract

**Introduction:**

Lung cancer (LC) is the most common cause of cancer death in the world and associated with significant economic burden. We conducted a review of published literature to identify prognostic factors associated with LC survival and determine which may be modifiable and could be targeted to improve outcomes.

**Methods:**

The exceptionally large volume of LC prognostic research required a new staged approach to reviewing the literature. This comprised an initial mapping review of existing reviews or meta-analyses, based on titles and abstracts, followed by an overview of systematic reviews evaluating factors that independently contribute to lung cancer survival. The overview of reviews was based on full text papers and incorporated a more in-depth assessment of reviews evaluating modifiable factors.

**Results:**

A large volume of published systematic reviews and meta-analyses were identified, but very few focused on modifiable factors for LC survival. Several modifiable factors were identified, which are potential candidates for targeted interventions aiming to improve cancer outcomes. The mapping review included 398 reviews, of which 207 investigated the independent effect of prognostic factors on lung cancer survival. The most frequently evaluated factors were novel biomarkers (86 biomarkers in 138 reviews). Only 15 modifiable factors were investigated in 20 reviews. Those associated with significant survival improvement included normal BMI/less weight loss, good performance status, not smoking/quitting after diagnosis, good pre-treatment quality of life, small gross volume tumour, early-stage tumour, lung resection undertaken by a thoracic/cardiothoracic surgeon, care being discussed by a multidisciplinary team, and timeliness of care.

**Conclusions:**

The study utilised a novel approach for reviewing an extensive and complicated body of research evidence. It enabled us to address a broad research question and focus on a specific area of priority. The staged approach ensured the review remained relevant to the stakeholders throughout, whilst maintaining the use of objective and transparent methods. It also provided important information on the needs of future research. However, it required extensive planning, management, and ongoing reviewer training.

**Electronic supplementary material:**

The online version of this article (10.1186/s13643-019-1087-4) contains supplementary material, which is available to authorized users.

## Background

The prognosis for lung cancer is poor, with a 12% average 5-year survival rate in Europe [1] falling to only 10% in England and Wales [1]. However, there is considerable clinical heterogeneity between the patients comprising the overall population with lung cancer. If identified early enough, radical treatment including surgery can provide the best chance of a cure. Approximately 80% of lung cancers are non-small cell types, which spread less rapidly than small cell lung cancer, but still have poor prognosis [2].

A prognostic factor is any measure that is associated with the risk of future health outcomes in those with existing disease [3], including sociodemographic factors, patient characteristics, health seeking behaviours, health service factors, and clinical characteristics. Genuine prognostic factors can play an important role in pathways towards improved clinical outcomes [4]. They are crucial in developing treatment plans and providing reliable information on prognosis or survival. However, the potential value of a prognostic factor is dependent on the availability of consistent evidence of its prognostic ability across multiple studies [4].

Prognostic research aims to understand the course, determinants, or probability of outcome in a cohort. In contrast, prognostic factor research is more specific and aims to identify independent factors that might be useful modifiable targets for interventions to improve outcomes, building blocks for prognostic models, or predictors of differential treatment response [4]. Prognostic research is summarised as four inter-related research themes in a framework introduced by the PROGnosis RESearch Strategy (PROGRESS) partnership: theme 1, overall prognosis research, investigates the average prognosis or likely course of disease [5]; theme 2, prognostic factor research, explores the independent impact of individual prognostic factors on lung cancer survival [4]; theme 3, prognostic model research, aims to develop, validate, or assess the impact of a statistical model which combines multiple factors from which the risk of lung cancer survival can be calculated for individual patients [6]; and finally, theme 4, stratified medicine research, aims to identify characteristics of patients likely to derive most clinical benefit or least harm from a specific treatment [7].

We aimed to identify prognostic factors consistently shown to be associated with lung cancer survival in published literature, and determine which factors may be modifiable and could be targeted to improve survival. Systematic reviews provide the gold standard approach for synthesising the evidence as they use standardised and empirically tested methods to minimise bias and error. However, the literature on prognostic factors in lung cancer is increasing exponentially [8], with hundreds of prognostic factors having already been identified [9]. A comprehensive, in-depth systematic review of primary studies was not feasible within a reasonable timescale. A rapid review approach was therefore developed based on elements of existing systematic review methodology and accepted practice [10, 11]. One approach to developing a summary of the existing evidence in a short timeframe is to conduct a systematic review (or overview) of existing reviews [12, 13]. Overviews of reviews are a relatively new and increasingly popular approach [14, 15]. Evidence mapping is another new tool that has been developed for addressing the challenges of a large evidence base, and belongs to the family of systematic approaches to reviewing the evidence [16, 17]. The methods for conducting, interpreting, and reporting overviews of reviews are still in their infancy, and guidance for their conduct is limited [15, 18, 19]. Guidance is especially limited for the less common types of overview, such as those addressing reviews of prognosis or diagnostic test accuracy [15]. The methodology for conducting systematic reviews of prognostic research is also still evolving with no accepted guidance on how best to conduct them [20].

The current review provides an example of the development and application of the combined use of these newly evolving methods for reviewing an extensive body of research evidence, in this instance on prognostic factors to inform decision-making. This paper focuses on the methodological approach used and is intended to inform those with an interest in review methods; it does not provide an in-depth presentation of the findings of prognostic factors associated with lung cancer.

## Methods

### Review methods

We conducted a pragmatic review of reviews using a three-stage approach, as outlined in Table 1. This included an initial mapping review of the prognostic research (stage 1), an overview of reviews of prognostic factor research (stage 2), followed by a more in-depth evaluation of modifiable factors (stage 3). The overall approach was guided by accepted methodological and reporting standards for systematic reviews [10, 11, 21]. The mapping review was informed by the methods used by the Evidence for Policy and Practice Information (EPPI) Centre and the overview of reviews by guidance for conducting systematic reviews of prognostic factors [22, 23].

**Table 1:** Summary of the review process

Stage	Elements of the review process
Stage 1: initial systematic mapping review of prognostic research	A descriptive map of prognostic research for lung cancer survival based on the assessment of titles and abstracts only. ● Classification of relevant reviews and meta-analysis according to PROGRESS research themes 1–4, lung cancer type, number and type of prognostic factors investigated, and publication type. ● Identification of all prognostic factors, from which a comprehensive coding scheme was developed. ● Identification of prognostic factors deemed to be potentially modifiable (reviewed by two independent public health and clinical stakeholders).
Stage 2: overview of systematic reviews of prognostic factor research	A more in-depth review of a subset of systematic reviews focusing on prognostic factor research (PROGRESS research theme 2) based on the assessment of full text publications. ● Summary of key data from included reviews. ● Coding of reviews according to the prognostic factors they addressed. ● Summary of all prognostic factors investigated by each review, including whether or not they were significantly associated with survival^†^, and the direction of the impact.
Stage 3: in-depth evaluation of potentially modifiable factors	A more in-depth evaluation of the results of included reviews reporting modifiable factors. ● Summary of the magnitude of the effect of modifiable prognostic factors (where possible).

^†^The term ‘significant’ denotes statistical significance and refers to the results of either the regression or meta-analyses (pooled analysis) or, where no pooled analysis was undertaken, to more than 50% of studies in narrative syntheses. For reviews that reported both pooled analysis and narrative synthesis, this was based on the results of the pooled analysis. Where significant findings were based on only a single study within a review, this was highlighted.

The review team was supported by public health and clinical stakeholders. The underlying purpose of the study, which was funded by Public Health Wales (PHW), was to inform the development of multivariate modelling of Welsh data proposed by the Welsh Cancer Intelligence and Surveillance Unit (WCISU). The stakeholders, in this instance, were therefore the director of WCISU (DWH) and a Consultant Respiratory Physician (GC). The stepped approach enabled us to provide WCISU with preliminary findings at the end of each stage and obtain ongoing feedback on prognostic factors that were likely to be the most relevant for both the multivariate model development, and policy and clinical decision making. The whole review process was also guided by monthly meetings with the stakeholders.

### Search strategy

MEDLINE, EMBASE, Cochrane Library, and Web of Science were searched in December 2015 using a strategy based on published prognostic factors search strategies [24], plus a search filter for systematic reviews and meta-analyses (Appendix A in Additional file 1) [25]. Searches were limited to 1990 onwards in order to be clinically and methodologically relevant [26, 27] but were not limited by language. All bibliographic data retrieved by the searches were imported into Endnote X5 and de-duplicated.

### Selecting studies for inclusion

The titles and abstracts were screened by two independent reviewers (SN, ZHT, TL, RP), using the criteria outlined in Table 2. Relevant references were coded according to the PROGRESS framework theme (stage 1). Articles published in English that appeared to report systematic reviews of prognostic factor studies (PROGRESS theme 2) were retrieved in full and re-evaluated (RL, MH, ND, SN, MAS, AH) for inclusion in the subsequent overview of reviews (stage 2). Disagreements regarding relevance were resolved by discussion or, if necessary, consulting a third reviewer.

**Table 2:** Inclusion criteria

	Inclusion criteria	Exclusion criteria and notes
Study design	Systematic reviews or meta-analyses.	Editorials or commentaries on included reviews. Meta-analyses not conducted as part of a systematic review were excluded from the overview of reviews (see text below).
Target population	Patients with a primary diagnosis of lung cancer reported separately from other cancers.	Reviews of mixed cancers were only considered if they included > 1 lung cancer study, with findings reported separately from other cancers. Studies of prognostic or predicative factors for developing lung cancer, in an otherwise healthy population, were not included. Lung cancer studies not reporting on histological subtypes were excluded for the overview of reviews (see text below).
Type of prognostic factors (exposure)	Any prognostic or predictive factors relating to the patient, tumour, socioeconomic position, or healthcare provider and system were included. Reviews of single or multiple factors were included.	The review did not include studies evaluating the effectiveness of treatments to improve survival. However, predictive factors of treatment outcomes (PROGRESS theme 4 research) were considered for inclusion in the mapping review.
Outcome measure	Overall or cancer specific survival.	–
Language and publication type	No language restrictions were used during the searches and mapping review.	Non-English language publications and conference abstracts were not included in the overview of reviews.

#### Systematic reviews

Studies were assessed for eligibility as a systematic review based on the eight essential elements outlined in the Cochrane Handbook [11] and the UK Centre for Reviews and Dissemination’s (CRD’s) guidance [10]. These include the following:

i. A clearly framed research question
ii. Explicit search criteria
iii. A search of more than one reference database
iv. Pre-defined eligibility criteria for studies
v. Explicit and reproducible methodology
vi. A systematic presentation of the characteristics of included studies
vii. The assessment for the validity of the findings of the included studies
viii. A narrative or quantitative synthesis of the included evidence.

The initial mapping review was based on titles and abstracts only, and therefore, a pragmatic and more liberal definition of a systematic review was used during this stage. However, for the purpose of the overview of reviews, studies had to meet four of the essential criteria (i, ii, iv, viii) for inclusion. Systematic reviews of prognostic factors may be of inferior quality compared with reviews of clinical effectiveness and are possibly less likely to include critical appraisal of included studies. It was therefore decided to use some of the ‘essential’ elements (iii, v, vi, vii) as quality, rather than inclusion, criteria. Some studies were retrieved in full during the initial mapping review in order to check eligibility, as the reference details were insufficient to make this judgement. Studies that reported a meta-analysis that was not based on a systematic search of the literature were not included in the second stage of the review.

#### Meta-analysis

Meta-analyses using individual patient data (IPD), where raw data from multiple studies are synthesised, are considered the gold standard for synthesising prognostic factor studies [28, 29]. However, they must be based on a systematic approach and attempt to include all relevant studies in order to avoid producing biased estimates or erroneous conclusions. Meta-analyses of IPD including only studies from a single research group are unlikely to include all relevant data and were excluded.

#### Lung cancer subtypes

Studies reporting on lung cancer as a whole, without distinguishing histological subtypes, were excluded from the overview of reviews unless they investigated prognostic factors that stakeholders deemed to be important and modifiable. Studies that were included in the initial mapping review and reported on lung cancer as a whole were first checked to see if they investigated prognostic factors deemed to be ‘modifiable’ (see the ‘Coding studies and data extraction’ section below). Those that did were then presented to the stakeholders to decide whether they should be included, based on the importance of the modifiable factor.

### Coding studies and data extraction

As part of the initial mapping review, relevant references were coded according to the underlying research questions investigated (prognostic research theme), whether they aimed to evaluate single or multiple factors and whether they were published in the English language (Appendix B in Additional file 2). This coding was undertaken, using Microsoft Excel, by one reviewer and checked by a second reviewer (SN, ZHT, TL, RP, RL). The type of lung cancer and prognostic factors investigated were also recorded. These data were then used in an iterative manner to develop a comprehensive list of codes for the prognostic factors and lung cancer types. The initial lists were developed by one reviewer (RL) and checked by a second (RP). Included prognostic factors were grouped using the following categorisation or domain headings:

- tumour characteristics (histology);
- clinical characteristics (routinely assessed biological variables);
- ‘new’ biomarkers (biological factors not used in routine practice);
- metabolic criteria;
- patient characteristics;
- healthcare provider and system; and
- other.

The data extraction and coding of papers retrieved in full (stage 2) were conducted by one reviewer (MH, ND, SN, MAS, AH, RL), with the first 10–15 checked by a second reviewer (RL, MH). The discrepancies were discussed at a weekly team meeting, and the instructions for coding and data extraction updated accordingly. Because of the complexity of the coding, some additional ongoing checking was conducted whilst developing summary tables of the included reviews and summarising the overall findings. The type of data extracted during stage 2 is provided in the summary tables presented in Additional files 3 and 4 (Appendix C and D), and included the overall aim; the year in which the searches were conducted; type of lung cancer and histological subtypes included; the number and type of prognostic factors investigated; the number of included studies; the sample size range; whether included studies were retrospective, prospective, or a mixture of both; whether multivariate analyses were conducted; the type of synthesis and summary measure used; whether additional analyses were undertaken; results; and author’s conclusions. A further, in-depth evaluation was conducted of included reviews of modifiable factors, described below. The data were extracted using Microsoft Access, which allowed the various codes to be selected from.

The list of prognostic factors developed as part of the mapping review was assessed by two independent public health and clinical stakeholders (DWH and GC) to identify those that were deemed to be potentially modifiable.

### Quality assessment

The quality of included reviews (in stage 2) was assessed using eight essential elements of a systematic review [10, 11], whilst reviews that investigated modifiable prognostic factors were additionally appraised using A Measurement Tool to Assess Systematic Reviews (AMSTAR) [30].

### Synthesis

The findings of existing reviews can be utilised within an overview of reviews or integrated into a new review in different ways [31]. The current review represents a rapid review approach, and as such, we chose not to disaggregate and/or re-analyse the existing review findings. Rather, we used the complete reviews and combined their findings in a narrative synthesis.

The findings of the overview of reviews were summarised and analysed using structured tables, listing all the factors investigated by each review, whether or not they were significantly associated with survival, and the direction of impact. Some systematic reviews included a meta-analysis or meta-regression (pooled analysis), whilst others only presented a narrative synthesis. Prognostic factors that were identified as being ‘significantly’ associated with survival were those that were shown to be statistically significant in either pooled analysis or by more than 50% of studies in the narrative synthesis. For reviews that reported both pooled analysis and narrative synthesis, this was based on the results of the pooled analysis. Pooled analyses and narrative syntheses were summarised separately. Where significant findings were based on only a single included study within a review, this has been highlighted. The magnitude of effects was only extracted for modifiable factors and presented in separate tables. This included both the summary effect estimate and 95% confidence intervals from both univariate and multivariate analysis, where presented.

## Results

### Mapping review

**Fig. 1:**
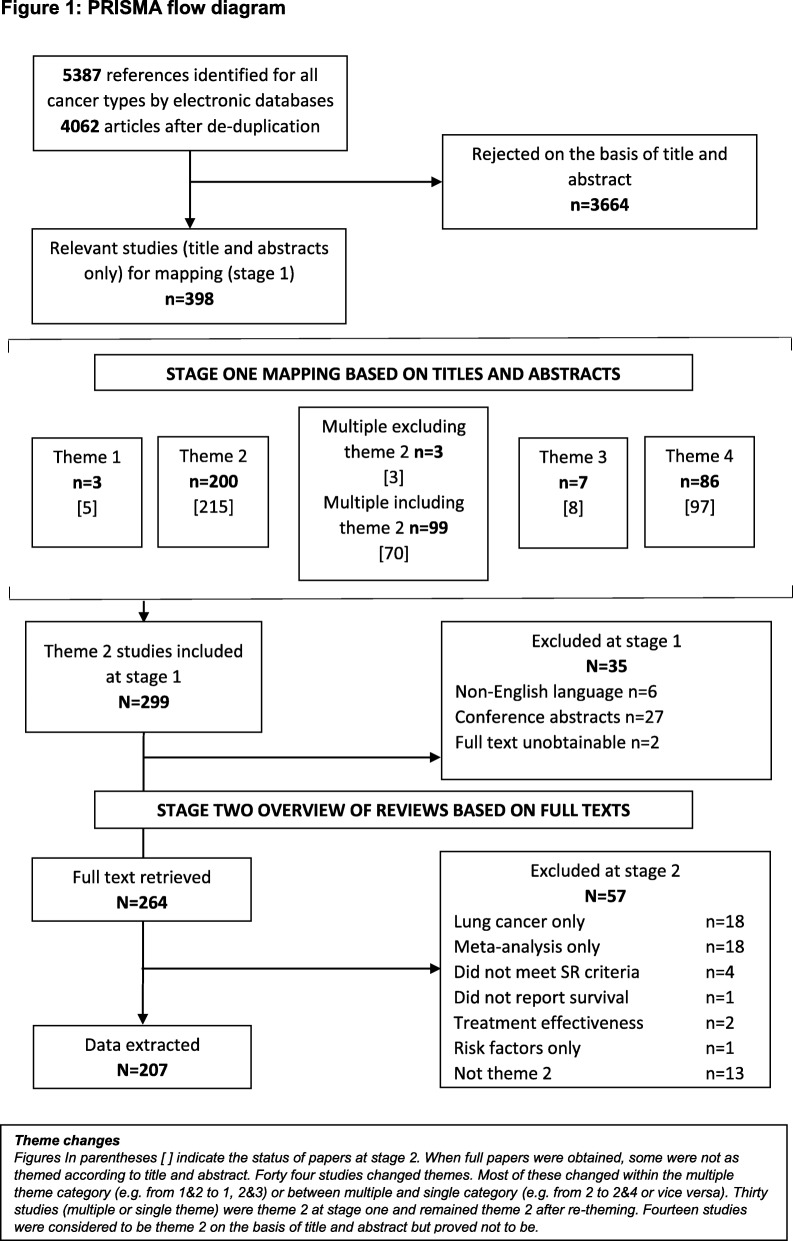
PRISMA flow diagram

Database searches identified 4062 references after de-duplication, of which 398 were eligible for the mapping review. Of these, 264 were retrieved in full, reassessed, and re-themed if appropriate; 207 were subsequently included in the overview of reviews (Fig. 1). A summary of the studies included in the mapping review, indicating the prognostic factors addressed and the type of lung cancer investigated, is available in the Additional file 2 (Appendix B). The final distribution of included references across the four PROGRESS themes was as follows: theme 1, overall prognosis, *n* = 12; theme 2, prognostic factors, *n* = 299; theme 3, prognostic models, *n* = 20; and theme 4, stratified medicine, *n* = 175 (figures differ from those in the PRISMA diagram because 102 studies covered multiple research questions).

### Overview of reviews of prognostic factor research

The findings presented here represent a condensed version of the results to provide an illustration of the quantity, breadth, and type of data provided by the review. More details of the included studies are provided in the additional files.

The overview of reviews included 207 systematic reviews investigating the independent effect of prognostic factors on lung cancer survival (theme 2). Reference details and review characteristics are presented in Additional file 3 (Appendix C) along with the findings of the quality assessment.

Most reviews were recent; 105 (51%) had searches conducted between 2013 and 2015, and only 23 (11%) prior to 2007. Most (146/207, 71%) limited inclusion to patients with non-small cell lung cancer (NSCLC); five (2%) focused on small cell lung cancer (SCLC). Thirty two (15%) included any type of lung cancer, 21 (10%) covered multiple cancer sites including lung, one review limited inclusion to lung squamous cell carcinoma, one to lung adenocarcinoma, and one included multiple primary lung cancers. Most (149, 72%) reviews were from East Asia, 54 (26%) from North America, 33 (16%) from Europe, three from West Asia, and one from Southeast Asia. A complete list of prognostic factors and their association with survival is presented in Additional file 4 (Appendix D) and summarised in brief below. Three reviews aimed to identify *any* prognostic factor associated with survival for NSCLC and as such included over 50 prognostic factors. The findings of these reviews are summarised separately in Additional file 5 (Appendix E).

The most frequently evaluated prognostic factors were ‘new’ biomarkers; 86 were investigated in 138 reviews, although 47 (55%) were each only evaluated in a single review. Eighteen biomarkers were evaluated by three or more reviews: EGFR (*n* = 11), ERCC1 (*n* = 7), K-RAS (*n* = 5), microRNA-155 (*n* = 5), survivin (*n* = 5), CXCR4 (*n* = 4), E-cadherin (*n* = 4), microRNA-21 (*n* = 4), p53 (*n* = 4), CRP (*n* = 4), COX-2 (*n* = 3), FGFR1 (*n* = 3), HER-2 (*n* = 3), HIF-1alpha (*n* = 3), P16 (*n* = 3), TS (*n* = 3), VEGF-C (*n* = 3). EGFR mutation was not found to be a significant factor in 7/11 reviews; ERCC1 was associated with improved survival in chemotherapy patients, but not for patients receiving surgery alone. Poor survival was associated with the presence, high levels, or overexpression of survivin, K-RAS, p53, microRNA-21, CXCR4, VEGF-C, TS, P16, HIF-1alpha, and HER-2 and with reduced E-cadherin levels.

Twenty-one factors relating to tumour characteristics were investigated in 26 reviews; seven were investigated in three or more reviews: circulating tumour cells (CTCs) (*n* = 3), histology (*n* = 6), lymphovascular invasion (LVI) (*n* = 3), nodal status (*n* = 4), stage (*n* = 6), synchronous metastases (*n* = 3), and visceral pleural invasion (VPI) (*n* = 3). Stage is described in more detail under modifiable factors. The presence of CTCs, positive VPI, and LVI was found to be associated with significantly poorer survival in all reviews evaluating these factors. Adenocarcinoma was associated with significantly better survival than squamous cell or other histological subtypes in four out of five reviews.

Eight reviews evaluated the prognostic value of measuring the metabolic activity of the primary tumour. Volumetric parameters evaluated by fluorodeoxyglucose positron emission tomography, including high values of standardised uptake value (SUV), metabolic tumour volume, and total lesion glycolysis were consistently identified as significant prognostic factors of poor survival in all the reviews.

The survival impact of 11 clinical characteristics or routinely assessed biological variables was evaluated in 12 reviews, but none by more than two reviews. High or normal serum albumin and the presence of skin rash associated with targeted chemotherapy treatment (EGFR tyrosine kinase inhibitors) were evaluated by two reviews; both were found to be associated with significantly better survival.

Nine patient characteristics were investigated in nine reviews, five of them in two or more reviews: age (*n* = 3), gender (*n* = 5), performance status (*n* = 2), pre-treatment quality of life (*n* = 2), and smoking status (*n* = 3). Three out of five reviews identified male gender, and two out of three reviews found advanced age to be significant adverse predictors of survival. The remaining three factors are discussed in more detail under modifiable factors, along with BMI, which was only evaluated by one review.

Five factors categorised as ‘healthcare provider and system’ were investigated in five reviews: insurance status, multidisciplinary team (MDT) patient management, surgical procedural volume, surgeon specialty, and timeliness of care, all of which are discussed in more detail under modifiable factors.

Three prognostic factors categorised as ‘other’ were investigated by four reviews; two were only evaluated in a single review. Perioperative blood transfusion due to excessive bleeding, which was evaluated by two reviews, was found to be significantly associated with poor survival.

### Review of modifiable factors

One or both independent clinician and public health stake-holders identified twenty-eight modifiable prognostic factors at the mapping stage, summarised in Additional file 6 (Appendix F). However, one included review reported on body mass index (BMI) and weight loss together so these two factors were subsequently combined. Twenty reviews meeting the criteria for inclusion in the overview of reviews evaluated one or more modifiable factor, but only 15 factors were investigated by these reviews. (The remaining 12 factors are detailed in Additional file 6.) The included reviews are distinguished in this next section using their unique review identification (ID) number presented in brackets. Three reviews that reported findings for lung cancer as a whole, without distinguishing subtypes, did not meet the inclusion criteria but were included based on the importance and potential usefulness of the prognostic factor they evaluated, namely surgeon specialty and the annual volume of surgical lung resections for cancer (414), the presence of tuberculosis (TB) (5789), and duration of the intervals between presentation and diagnosis or treatment (722).

A description of the reviews that investigated modifiable factors is presented in Table 3. The reviews were fairly recent, with all but one conducted from 2007 onwards. However, five reviews (25%) only searched one reference database, and five (25%) did not include a summary of study characteristics. Only seven (35%) reviews reported conducting a quality assessment of included studies. A quality appraisal of the reviews using the AMSTAR tool is presented in Additional file 7 (Appendix G). Almost half of the reviews (9/20 45%) were poor quality, and only two (10%) were considered to be good quality (studies 494 and 695). Most of reviews did not consider unpublished/grey literature or studies not published in the English language. Most reviews did not include an assessment of the scientific quality of the included studies, and the characteristics of included studies were also poorly presented with known prognostic factors, such as age, sex, performance status, stage, and histology, not generally listed. Half of the reviews included patients with NSCLC, and six (30%) included the broader category of lung cancer, whilst none limited inclusion to just SCLC. Three reviews (15%) covered multiple cancer sites, including lung. The 20 modifiable factors investigated by included reviews are summarised in Table 4. However, as shown in the description of the prognostic factors, they were often analysed as multiple subcategories or histological subgroups with varying thresholds. The total number of prognostic factors investigated ranged from one to 20 for 17 reviews, whilst the remaining three evaluated ‘any’ prognostic factor associated with survival (Table 3). (These three reviews (182, 843, 1051) are also described in more detail in the Additional file 5.) Only eight reviews reported pooled analyses, using meta-analysis or meta-regression (Table 3), the results of which are presented in Additional file 8 (Appendix H). The level of statistical heterogeneity between included studies, where investigated, was found to be significant in all but one review (695). The findings of the remaining reviews were based primarily on a descriptive summary of whether the findings were statistically significant or not, or a system of ‘vote counting’.

**Table 3:** Summary of included reviews that investigated modifiable factors

Author, year	REV ID	Country	Search year*	DB	RM	CH	QA	Synthesis	PR theme	No. PFs evaluated**	LC type	No. of studies	Sample size range	Included UVA or MVA
Aboshi, 2014	182	Japan	2012	N	N	Y	N	MR	2, 4	13	NSCLC (late)	65	NS	NS
Ashworth, 2013	237	UK	2012	Y	Y	P	N	Narrative	1, 2	Any (total NS)	NSCLC (oligometastatic)	49 (23 in analysis of PFs)	NS (1064 in analysis of PFs)	MVA
Ashworth, 2014	105	UK	2012	Y	Y	Y	N	MR	1, 2, 3	20	NSCLC (oligometastatic)	20	6–262	Both
Behera, 2016	5815	US	2015	Y	N	Y	N	MA	2	2	NSCLC (I)	19 (11 survival)	8–110	NS
Berghmans, 2011	8434	Belgium	2009	N	N	P	N	Narrative	2	Any (> 50)	NSCLC (III)	39	42–2048	MVA
Brundage, 2002	1051	Canada	2001	N	N	N	N	Narrative	2	Any (169)	NSCLC	887	31–1281	MVA
Buttigliero, 2011	494	Italy	2007	Y	Y	Y	Y	Narrative	2	2	mxdC (LC/NSCLC)	25 (2 LC)	294–447	MVA
Carter, 2014	5362	US	2010	Y	P	N	Y	Narrative	2, 4	12	NSCLC (III–IV)	54	NS	MVA
Christopoulos, 2013	5789	Greece	2013	Y	Y	N	N	Narrative	2	1	LC	17	4–56 (NS for 3 studies)	NS
Deghaidy, 2005	923	Egypt	2004	N	Y	N	N	MA	2	1	LC (NSCLC/SCLC)	29	23–1342	NS
Florou, 2014	51	Greece	2013	Y	N	Y	N	Narrative	2, 4	1	mxdC NSCLC (early)/SCLC (limited)	20 (13 LC; 4 survival only)	66–2258	NS
Montazeri, 2009	703	Iran	2008	Y	N	Y	N	Narrative	2	1	mxdC NSCLC/SCLC	104 (26 LC)	30–651	MVA
Neal, 2015	8441	UK	2013	Y	Y	Y	Y	Narrative	2	15	mxdC (LC/NSCLC)	209 (20 LC)	103–566	NS
Olsson, 2009	722	US	2007	Y	Y	Y	Y	Narrative	2	1	LC	18	NS (2 studies with > 1000 pts)	Both
Parsons, 2010	695	UK	2008	Y	Y	Y	Y	MA	1, 2	1	LC (NSCLC/SCLC)	10	61–611	Both
Prades, 2015	5807	Spain	2012	N	Y	Y	N	Narrative	2	1	mxdC LC (LC/NSCLC)	51 (3 LC; 1 survival)	NS	NS
Salah, 2012	467	Jordan	2010 (IS)	Y	U	Y	N	MR	2	8	NSCLC (isolated met)	51	NS (total 62)	Both
Slatore, 2010	621	US	2008	Y	P	Y	Y	Narrative	2	1	LC (LC/NSCLC)	23 (9 survival)	249–693,697	Both
von Meyenfeldt, 2012	414	Netherlands	2011	Y	Y	P	N	MR	2	3	LC	19	987–90,088	MVA
Yu, 2015	5489	China	2014	Y	U	N	Y	MA	2	1	NSCLC	10	21–412	Yes

*Where the search dates were not reported, this was based on the latest publication year of included studies (IS)

**Three reviews, which evaluated ‘any’ prognostic factor included a minimum of 50 factors: Ashworth, 2013 (237); Berghmans, 2011 (8434); and Brundage, 2002 (1051)

*REV ID* review unique identification number, *DB* searched more than one reference database searched, *No.* number, *RM* used explicit and reproducible methodology used, *CH* a systematic presentation of the characteristics of included studies presented, *QA* assessment for the validity of the findings of the included studies conducted, *ADC* adenocarcinoma, *CTX* chemotherapy, *IS* included studies, *LC* lung cancer, *MA* meta-analysis, *met* metastasis, *MR* meta-regression, *MVA* multivariate analysis, *mxdC* mixed cancer, *NS* not stated, *N* no, *NSCLC* non-small cell lung cancer, *PF* prognostic factor, *PR* prognostic research, *RT* radiotherapy, *SCC* squamous cell carcinoma, *SCLC* small cell lung cancer, *surR* surgical resection, *sync* synchronous, *UVA* univariate analysis, *Y* yes

**Table 4:** Modifiable prognostic factors evaluated by included reviews

Author, year*	REV ID	Patient population	PF code	Description	Study design	Evaluable studies	Sample size range	Summary measure	Pooled analysis	Narrative synthesis***
Patient characteristics										
Carter, 2014	5362	NSCLC (III–IV)	BMI	Less weight loss or normal BMI	Retrospective	21	NS	NS		+ in 11 studies; <> in 10 studies
Berghmans, 2011	8434	NSCLC (III)	BMI	Body mass index	NS	NS	NS	NS		+ in 1 study; <> NS
Berghmans, 2011	8434	NSCLC (III)	Weight loss	Weight loss (no further details)	NS	NS	NS	NS		+ in 5 studies; <> NS
Brundage, 2002	1051	NSCLC (early)	Weight loss	No substantial weight loss	NS	6	55–207	NS		<> in 6 studies
Aboshi, 2014**	182	NSCLC (late)	PS	Percentage of patients in study with ECOG PS1 < 60 (vs ≥ 60)	Prospective	13	39–1217	OR	<>	
Berghmans, 2011	8434	NSCLC (III)	PS	Good performance status	NS	NS	NS	NS		+ in 13 studies; <> NS
Brundage, 2002	1051	NSCLC (early)	PS	High performance status	NS	13	69–408	NS		+ in 4 studies; <> in 9 studies
Carter, 2014	5362	NSCLC (III–IV)	PS	Better PS, ECOG 0-1/KPS < 70 (vs ≥ 2, or vs ≥ 80)	Retrospective	47	NS	NS		+ in 36 studies; <> in 11 studies
Berghmans, 2011	8434	NSCLC (III)	QoL	Quality of life (no further details)	NS	NS	NS	NS		+ in 1 study; <> NS
Montazeri, 2009**	703	mxdC (LC)	QoL	Pre-treatment (baseline) quality of life	NS	26	30–651	NS		+ in 24 studies; <> in 2 studies
Montazeri, 2009**	703	mxdC LC (NSCLC)	Qol	Pre-treatment (baseline) quality of life	NS	11	30–573	NS		+ in 10 studies; <> in 1 study
Montazeri, 2009**	703	mxdC LC (SCLC)	Qol	Pre-treatment (baseline) quality of life	NS	1	70	NS		<> in 1 study
Carter, 2014	5362	NSCLC (III–IV)	QoL	Better pre-treatment health related QoL/fewer symptoms	Retrospective	6	NS	NS		+ in 6 studies
Berghmans, 2011	8434	NSCLC (III)	Smoking status	Smoking (no further details)	NS	NS	NS	NS		+ in 1 study; <> NS
Brundage, 2002	1051	NSCLC (early)	Smoking status	Smoking habit (no further details)	NS	23	50–593	NS		+ in 2 studies; <> in 21 studies
Florou, 2014**	51	mxdC LC (limited SCLC)	Smoking status	Non-smoker—during treatment (vs smoker)	Retrospective	1	215	NS		+ in 1 study
Florou, 2014**	51	mxdC LC (limited SCLC)	Smoking status	Ex-smoker—at or after diagnosis (vs current smoker)	Prospective	1	284	NS		+ in 1 study
Florou, 2014**	51	mxdC LC (early NSCLC)	Smoking status	Ex-smoker—quit smoking before diagnosis (vs current smoker or never smoked)	Prospective	1	543	NS		<> in 1 study;
Florou, 2014**	51	mxdC LC (early NSCLC/limited SCLC)	Smoking status	Never smoked (vs ex- or current smoker)	Prospective	2	284–238	NS		<> in 1 study; + in 1 study
Parsons, 2010	695	NSCLC (mainly I–IIIA)	Smoking status	Continued smoking after diagnosis (vs quit smoking) in NSCLC	Mixed	4	93–311	HR	<> (unadj); − (adj, *n*=1)	
Parsons, 2010	695	SCLC (mainly limited)	Smoking status	Continued smoking after diagnosis (vs quit smoking) in SCLC	Retrospective	2	70–611	HR	−	
Carter, 2014	5362	NSCLC (III–IV)	Smoking status	Less/no smoking status	Retrospective	9	NS	NS		+ in 6 studies; <> in 3 studies
Healthcare provider and system										
Slatore, 2010**	621	LC	Insurance status	Medicaid (vs private, non-Medicaid, or other funded)	NS	4	3702–13,469	HR/OR		− in 4 studies
Slatore, 2010**	621	LC (NSCLC)	Insurance status	Commercial or other insurance (vs private insurance)	NS	2	336–1403	HR/OR		<> in 2 studies
Slatore, 2010**	621	LC	Insurance status	Medicaid/Medicare (vs Medicare)	NS	2	3094–26,073	HR		− in 2 studies
Slatore, 2010**	621	LC (NSCLC)	Insurance status	Medicaid/Medicare (vs Medicare)	NS	1	3,094	HR		− in 1 study
Slatore, 2010**	621	LC	Insurance status	Medicare HMO (vs Medicare Fee for Service)	NS	1	10,229	HR		<> in 1 study
Prades, 2015**	5807	mxdC (NSCLC)	MDT	MDT patient management	Prospective	1	NS	Survival rate		+ in 1 study
von Meyenfeldt, 2012**	414	LC	Procedural volume	High hospital annual volume of surgical resections (vs low volume)—post-operative mortality	NS	11 (10 in MR)	987–90,088	OR	+	+ in 6 studies; <> in 5 studies
von Meyenfeldt, 2012**	414	LC	Procedural volume	High hospital annual volume of surgical resections (vs low volume)—overall survival	NS	8 (7 in MR)	1097–40,754	HR	<>	+ in 5 studies; − in 1 study; <> in 2 studies
von Meyenfeldt, 2012**	414	LC	Procedural volume	High annual surgeon procedural volume (vs low volume)—post-operative mortality	NS	2	4841–24,092	HR	<>	+ in 2 studies
von Meyenfeldt, 2012**	414	LC	Surgeon	Surgeon specialty: general thoracic surgeon (vs general surgeon)—post-operative mortality	NS	3	19,745–86,538	HR	+	+ in 1 study; <> in 2 studies
von Meyenfeldt, 2012**	414	LC	Surgeon	Surgeon specialty: general thoracic surgeon (vs general surgeon)—overall survival	NS	2	1097–19,745	OR	<>	+ in 1 study; <> in 1 studies
von Meyenfeldt, 2012**	414	LC	Surgeon	Surgeon specialty: cardiothoracic surgeon (vs general surgeon)—post-operative mortality	NS	3	19,745–86,538	OR	+	+ in 1 study; <> in 2 studies
von Meyenfeldt, 2012**	414	LC	Surgeon	Surgeon specialty: cardiothoracic surgeon (vs general surgeon)—overall survival	NS	2	1097–19,745	HR		+ in 1 study; <> in 1 studies
Olsson 2009	722	LC	Timeliness of care	Shorter intervals to diagnosis or treatment	Mixed	15	NS	NS		− in 4 studies; <> in 8 studies; + in 3 studies
Neal, 2015	8441	mxdC (LC)	Timeliness of care	Shorter diagnostic interval: time from first seen in primary care to diagnosis	Mixed	4	122–378	NS		<> in 3 studies; + in 1 study
Neal, 2015	8441	mxdC (NSCLC)	Timeliness of care	Shorter treatment interval: time from first seen in primary care to treatment	Retrospective	2	415–495	NS		<> in 1 study; − in 1 study
Neal, 2015	8441	mxdC (NSCLC)	Timeliness of care	Shorter patient interval: time from symptom onset to first seen in primary	Retrospective	2	122–7358	NS		<> in 1 study; − in 1 study
Neal, 2015	8441	mxdC (LC)	Timeliness of care	Shorter time from symptom onset to diagnosis	Retrospective	1	NS (total 566)	NS		+ in 1 study
Neal, 2015	8441	mxdC (LC)	Timeliness of care	Shorter time from symptom onset to treatment	Retrospective	1	NS (total 103)	NS		<> in 1 study
Neal, 2015	8441	mxdC (NSCLC)	Timeliness of care	Shorter time from symptom onset to being seen in specialist care	Retrospective	1	NS (total 415)	NS		<> in 1 study
Clinical characteristics or routinely assessed biological variables										
Christopoulos, 2013**	5789	LC	TB	Active tuberculosis, TB (vs no TB)	Retrospective	5	NS	Median survival		− 1 in study; ? in 4 studies (no comparative data)
Buttigliero, 2011	494	mxdC LC	VitD level	Low serum vitamin D level	Prospective	2	294–447	HR		<> in 2 studies
Ashworth, 2014**	105	NSCLC (oligometastatic)	Stage	IB (vs IA)	Prospective	20	6–262	HR	<>	
Ashworth, 2014**	105	NSCLC (oligometastatic)	Stage	IIA (vs IA)	Prospective	20	6–262	HR	<>	
Ashworth, 2014**	105	NSCLC (oligometastatic)	Stage	IIB (vs IA)	Prospective	20	6–262	HR	− (unadj)	
Ashworth, 2014**	105	NSCLC (oligometastatic)	Stage	IIIA (vs IA)	Prospective	20	6–262	HR	− (unadj)	
Ashworth, 2014**	105	NSCLC (oligometastatic)	Stage	IIIB (vs IA)	Prospective	20	6–262	HR	− (unadj)	
Ashworth, 2013**	237	NSCLC (oligometastatic)	Stage	Stage I (vs III or IV)	Mixed	5	NS	NS		+ in 1 studies; <> in 4 studies
Aboshi, 2014**	182	NSCLC (late)	Stage	Percentage of patients in study with stage IV disease < 80 (vs ≥ 80)	Prospective	13	39–1217	OR	<>	
Salah, 2012**	467	NSCLC (solitary met)	Stage	Intra-thoracic stage III (vs stage II or I)	Retrospective	36	NS	HR	−	
Deghaidy 2005**	923	LC (NSCLC)	Stage	Stage I vs II (in NSCLC)	Mixed	10	23–226	RR	+	+ in 5 studies; <> in 5 studies
Deghaidy 2005**	923	LC (SCLC)	Stage	Stage I vs II (in SCLC)	Mixed	4	24–295	RR	+	+ in 3 studies; <> in 1 study
Deghaidy 2005**	923	LC (NSCLC)	Stage	Stage I vs III (in NSCLC)	Mixed	7	33–1342	RR	+	+ in 4 studies; <> in 3 studies
Deghaidy 2005**	923	LC (SCLC)	Stage	Stage I vs III (in SCLC)	Mixed	3	27–92	RR	+	+ in 2 studies; <> in 1 study
Deghaidy 2005**	923	LC (NSCLC)	Stage	Stage II vs III (in NSCLC)	Mixed	7	57–961	RR	+	+ in 3 studies; <> in 4 studies
Deghaidy 2005**	923	LC (SCLC)	Stage	Stage II vs III (in SCLC)	Mixed	3	25–95	RR	+	<> in 3 studies
Deghaidy 2005**	923	LC (NSCLC)	Stage	Stage III vs IV (in NSCLC)	Mixed	6	21–2198	RR	+	+ in 3 studies; <> in 3 studies
Deghaidy 2005**	923	LC (SCLC)	Stage	Stage III vs IV (in SCLC)	Mixed	2	22–319	RR	<>	<> in 2 studies
Carter, 2014	5362	NSCLC (III–IV)	Stage	Less advanced stage, mainly IIIB (vs IV)	Retrospective	37	NS	NS		+ in 21 studies; <> in 16 studies
Behera, 2016**	5815	NSCLC (I)	Stage	Adenocarcinoma in situ, AIS (vs minimally invasive adenocarcinoma, MIA)	NS	11	8–110	Survival rate	<>	
Berghmans, 2011	8434	NSCLC (III)	Stage	Less advanced stage	NS	NS	NS	NS		+ in 6 studies; <> NS
Brundage, 2002	1051	NSCLC (early)	Stage	Less advanced stage	NS	103	31–593	NS		+ in 68 studies; <> in 35 studies
Berghmans, 2011	8434	NSCLC (III)	T volume	Tumour volume	NS	NS	NS	NS		+ in 2 studies; <> NS
Yu, 2015**	5489	NSCLC (unresectable)	GTV	Small gross tumour volume, GTV < 112 cm3vs (vs large GTV >= 112cm3)	NS	5	32–115	HR	+	
Prognostic factors classified as ‘other’										
Ashworth, 2014	105	NSCLC (oligometastatic)	Surgical treatment	Surgical primary LC treatment (vs non-surgical)	Prospective	20	6–262	HR	+ (unadj)	
Ashworth, 2013	237	NSCLC (oligometastatic)	Surgery	Type of thoracic resection: lobectomy (vs pneumonectomy)	Mixed	2	NS	NS		+ in 1 studies; <> in 1 studies
Berghmans, 2011	8434	NSCLC (III)	Surgery	R0 (complete resection)	NS	NS	NS	NS		+ in 4 studies; <> NS
Brundage, 2002	1051	NSCLC (early)	Surgery	Surgical procedure (no further details)	NS	15	43–593	NS		+ in 4 studies; <> in 11 studies

*Three reviews which evaluated ‘any’ prognostic factor included a minimum of 50 factors: Ashworth, 2013 (237); Berghmans, 2011 (8434); and Brundage, 2002 (1051). Beghmans, 2011, (which included 39 studies) provided a list of factors found to be significant in multivariate analysis, but did not indicate the number of studies that had evaluated each factor; the description of each factor was also minimal. For Berghmans, 2011 (8434), only factors found to be significant by included studies (reporting multivariate analysis) were listed with minimal descriptors; the total number of studies which evaluated each factor was not reported. Brundage, 2002 (1051), included separate analysis for studies that investigated prognostic factors in resected NSCLC. The description of the prognostic factors were minimal and are reported verbatim here (not stated what the comparator was)

**The actual results for these studies are presented in Additional file 8 (Appendix H)

***Narrative synthesis reported by the review

*REV ID* review unique identification number, *BMI* body mass index, *GTV* gross tumour volume, *HR* hazard ratio, *LC* lung cancer, *MR*-meta regression, *MDT* multidisciplinary team, *mxdC* mixed cancer, *NS* not stated, *NSCLC* non-small cell lung cancer, *OR* odds ratio, *PS* performance status, *RR* relative risk, *SCLC* small cell lung cancer, *TB* tuberculosis, *vs* versus

#### Patient characteristics

Four patient-related modifiable factors were investigated: BMI, performance status (PS), quality of life (QoL), and smoking status. These were evaluated in seven reviews, five of which reported a narrative synthesis, one a meta-analysis, and one a regression-analysis. Two of the reviews (1051, 8434) reporting narrative syntheses aimed to evaluate any prognostic factor associated with survival and therefore included both a large number of factors and studies (Tabl 3). One of these reviews (8434) only reported the total number of included studies that identified these factors as significant in their multivariate analysis, without providing the denominators.

Less weight loss or normal BMI at diagnosis was found to be associated with significantly better survival in advanced NSCLC, according to 11/21 studies in one review (5632) and 6 studies in another (which did not report the denominators) (8434). Four reviews investigated the effect of PS (by various measures of wellbeing and activities of daily life) in advanced or metastatic NSCLC. Better PS was found to be significantly associated with survival in 36/47 studies in one review (5362) and 13 in another (8434), whilst it was only found to be a significant factor in 4/13 studies in a third review (1051). The final review did not find it to be a significant influencing factor associated with survival extension, but this analysis was based on first-line chemotherapy trials where the majority of patients had a good PS (182). Three reviews evaluated pre-treatment health-related QoL. Two reviews found that better QoL or fewer symptoms were significantly associated with increased survival in patients with late stage NSCLC, according to all six included studies (5362) or one study (8434). The third review was both poorly conducted and reported, but also found that pre-treatment (baseline) global quality of life scores or some aspects of quality of life measures were significant independent predictors of survival in 24/26 lung cancer studies (703).

Five reviews, including one good quality review (695), evaluated the effect of smoking status. Two reviews showed that quitting smoking at or after diagnosis was associated with better survival than continued smoking in limited SCLC or early NSCLC, but these findings were only significant in SCLC (695, 51). Not smoking was also found to be associated with significantly better survival than smoking in three reviews (8434, 5362, 51), whilst a fourth only reported significant findings in 2/23 studies (1051). One review reported significant findings associated with ‘less/no smoking status’ and the outcome of advanced NSCLC in multivariate analysis of 6/9 studies (5362); the other reported better survival in patients with limited SCLC who did not smoke during chemo-radiotherapy treatment in one study, although another study reported that neither smoking status at time of diagnosis nor pack years smoked had a significant impact on survival for limited SCLC (8434); a third study found that smoking status both before and after surgery was significantly associated with survival in early NSCLC (51).

#### Healthcare provider and system

Six modifiable factors that were categorised as healthcare provider and system were included: insurance status, multidisciplinary team (MDT) patient management, surgeon, procedural volume, and timeliness of care. These were investigated in five reviews, most of which used a narrative synthesis.

One review from the USA investigated the association between insurance status and survival in patients with NSCLC (621). Patients with Medicaid or no insurance had worse survival, higher incidence rates, and later stage at diagnosis than those with private or Medicare insurance, and were also less likely to undergo curative procedures than those with private, Medicare, or other funded insurance in 6/9 studies.

One review investigated the impact of MDTs on patient outcomes (5807). This was a poor quality systematic review that considered any cancer type. Patient care being managed by an MDT was identified as a significant factor associated with improved survival in NSCLC, according to a single study.

Two reviews evaluated the association between time to diagnosis or treatment, and survival. Both reviews presented a narrative synthesis based on vote counting. The patient journey from symptom onset to diagnosis or treatment initiation can be broken down into multiple different intervals [32]. One review (722) that investigated the effects of timeliness of care for lung cancer as a whole did not differentiate between the diagnostic and treatment interval, whilst the second review (8441), which evaluated any cancer type, but reported separate data for NSCLC, did distinguish between interval types (outlined in Table 4). In the review limited to lung cancer, shorter intervals were associated with significantly improved survival in three out of eighteen studies, and reduced survival in four; eight studies did not report significant findings (722). The second review reported that shorter intervals between either symptom onset or being seen in primary care, and diagnosis were associated with improved survival in NSCLC, according to a single study (in 1/1 and 1/4 studies, respectively) (8441). However, shorter intervals between symptom onset and first being seen in primary care, and being seen in primary care and starting treatment, were associated with reduced survival, according to one out of 2 studies. The discordant finings reported by both these reviews are likely to be due to the ‘waiting time paradox’ [33–35], where patients with rapidly growing or metastatic tumours are more likely to experience symptoms that draw attention to the underlying cancer, but have poor outcomes.

One review evaluated the effect of both surgeon specialty and volume of lung resections for cancer in a hospital or by a surgeon on post-operative mortality or overall survival (414). Hospitals performing a high-volume of resections for lung cancer had significantly better outcomes, in terms of post-operative mortality, than low-volume hospitals, but there was no significant difference between the two for overall survival. Surgeon procedural volume showed no significant effect on post-operative mortality, but general surgeons had significantly higher mortality risks than general thoracic or cardiothoracic surgeons.

#### Clinical characteristics or routinely assessed biological variables

Two factors categorised as clinical or routinely assessed biological variables were included: tuberculosis (TB) and serum vitamin D level. Each factor was only evaluated by a single review using a narrative synthesis. According to a very poorly conducted and reported review, the median survival in patients with active TB was 4 months compared with 8 months in patients without TB in one study (5789). Low serum vitamin D was not found to be associated with survival in NSCLC, according to both relevant studies in one good quality review (494).

#### Tumour characteristics

Nine reviews evaluated stage, and two investigated tumour volume as prognostic factors for survival. Three reviews evaluated stage along with other factors in regression analyses. In one review, the regression analyses were undertaken using IPD obtained from the authors of the primary studies (105), and in another, IPD were extracted from the published studies (467).

Tumour stage was identified as a significant factor associated with overall survival on univariate, but not multivariate analysis in two of the reviews (105, 467), and not a significant factor in the multivariate regression analysis undertaken within the third review (182). A further review conducted a series of eight meta-analyses comparing stages I versus II, I versus III, II versus III, and III versus IV, in both NSCLC and SCLC. There was a significant reduction in the relative risk of death for earlier staged tumours when compared with later stages in six meta-analyses (923). One review evaluated the prognostic differences between adenocarcinoma in situ and minimally invasive adenocarcinoma using a meta-analysis, and found no significant differences in survival rates between the two groups (5815). The remaining four reviews included a narrative synthesis. Three found that less advanced stage (mainly IIIB) was associated with significantly better survival than stage IV in the majority of included studies in three reviews (29, 8434, 1051), whilst the fourth found that stage I oligometastatic NSCLC was associated with significant better survival than stage III–IV in 1/5 studies (237). One review investigated the association between gross tumour volume and the prognosis of patients with unresectable NSCLC after radiotherapy treatment. The overall survival was significantly less in patients with a large (≥ 112 cm^3^), compared to small (< 112 cm^3^), gross tumour volume (5489). A second review noted that tumour volume was identified as a significant factor for stage III NSCLC in two studies of multivariate analysis (8434).

## Discussion

This paper provides an example of the development and application of a pragmatic and innovative approach for reviewing an extensive and complicated body of research evidence to inform decision-making. The review aimed to identify prognostic factors associated with lung cancer survival. It focused on the findings of modifiable factors, but also provided an overview of the evidence relating to any prognostic factor, as well as a useful resource of all the reviews and meta-analyses of broader prognostic research questions. This was achieved using a three-staged reviewing approach, which included an initial mapping review of lung cancer prognostic research, based on titles and abstracts, followed by an overview of systematic reviews evaluating factors that independently contribute to lung cancer survival. A compressive *prognostic factor* coding scheme was developed as part of the mapping review, from which a list of modifiable factors was drawn, based on input from key stakeholders. The overview of reviews, which was based on full text papers, aimed to provide a description of the available reviews and their overall findings. It provided both a summary of the prognostic factors found to be associated with survival and a means of identifying the better quality and more recent reviews. It also allowed us to assess whether there was consistency between the overall findings of multiple reviews assessing the same prognostic factors. The quality of included reviews was assessed using the eight essential criteria for a systematic review [10, 11]. A more in-depth evaluation was conducted of reviews that considered one or more modifiable factors. This included further data extraction including the magnitude of the effect and quality appraisal using the AMSTAR checklist [30].

The reviewing approach was successful in providing a systematic and transparent description of the nature and coverage of an exceptionally broad prognostic research field relating to lung cancer survival. It included an overview of more than 80 individual prognostic factors associated with survival, with a special focus on 15 modifiable prognostic factors, such as critical patient characteristics relevant to public health interventions and healthcare provider characteristics relevant for organisational and systemic healthcare redesign.

A well-conducted systematic review provides an essential evidence resource for informing decision-making. However, as highlighted by Ioannidis et al. and identified by our own review, there is a massive production of unnecessary, misleading, and conflicted systematic reviews [36]. A frequent problem faced by decision-makers is the difficulty in interpreting the findings of multiple reviews reporting inconsistent results and conclusions [21, 37]. A well-conducted overview of reviews provides an objective assessment of whether the conclusions of individual reviews are consistent or not, as well highlighting the most relevant and high quality reviews. The identification of consistent findings across multiple high-quality systematic reviews also provides more confidence in the value of a prognostic factor. The current reviewing approach was developed with this in mind.

Systematic mapping reviews and overview of reviews can be viewed as rapid review approaches. The combined stepped approach has far ranging applications for present and future healthcare policy priorities with a complicated epidemiology (e.g. other cancers and chronic or degenerative conditions), where a review of wide-ranging systematic reviews is needed in a relatively short timeframe. Systematic mapping reviews are generally used to structure a broad research area, whilst systematic reviews tend to focus on gathering evidence to answer a more focused research question. Both aim to identify and summarise the evidence base in an objective, repeatable, and transparent way. Where there is a broad evidence base, prioritising evidence from systematic reviews limits unnecessary duplication, minimises resources needed to screen and summarise primary level evidence, and reduces potential bias and/or error, which could be incurred by reviewing primary evidence rapidly [38]. The advantage of using a staged approach is that it provides a valuable description of the available research. The inclusive nature of the mapping review enabled us to identify both individual prognostic factors and reviews that evaluated prognostic factors of specific interest, which would have been excluded from a more focused review. The staged approach also enabled us to reconsider reviews that evaluated prognostic factors deemed to be important and modifiable by the stakeholders, but did not meet the inclusion criteria for the overview of reviews, in a transparent and objective way.

The initial mapping review (stage 1) and the overview of reviews (stages 2–3) represent two consecutively funded studies, each with a 9-month duration (1July 2015–31 March 2016 and 1 July 2016–31 March 2017). The breadth and volume of the evidence base meant that a conventional systematic review of primary studies was not feasible within the overall timeframe. Furthermore, although the primary focus was to identify individual candidate prognostic factors (in prognostic factor research), these are sometimes investigated within, or alongside, other prognostic research studies. Our staged reviewing approach, utilising existing reviews, enabled us to complete both studies on time, whilst maintaining the necessary breadth of the evidence review. However, completing the entire review over 18 months still proved very challenging. This was compounded by the fact that the review was conducted by multiple reviewers working part-time, most of whom worked only on the mapping review (stage 1) or overview of reviews (stage 2–3). In order to maintain consistency, there were weekly team meetings, but on occasion, individual reviewers had to suspend work until queries could be addressed at the next meeting, or re-check work to ensure it was correct. The volume of relevant systematic reviews was far more than expected, and the fact that most of these were fairly recent and poor quality meant that the time available for a more in-depth review of modifying factors (stage 3) was limited. The diversity and complexity of the evidence base also made the review work challenging and time consuming. The main challenge here, as well as the large number of reviews, was that existing reviews varied in terms of the methods used for searching and synthesising the evidence. Furthermore, the primary studies within each review also varied in terms of sample size, population and lung cancer types, methods used to identify prognostic factors, the way in which the factors were measured (as a continuous or binary measure), the thresholds used to categorise the prognostic factors, and the type of variables that were adjusted for in the analysis. In order to expedite the review, we increased the number of reviewers working on it. This also brought its challenges, as it required everyone to implement the complicated inclusion criteria and coding schemes, with much checking required to achieve consistency.

The rapid review approach was based on a narrative synthesis and utilised the overall findings and conclusions of the included reviews [31]. The magnitude of effect of prognostic factors was only considered for the modifiable factors. However, the findings of most reviews were based on either the statistical significance of included studies or ‘vote counting’, neither of which is capable of accurately evaluating the association with survival. A review of reviews generally relies on the appraisal and data extraction of previous reviews rather than going back to the primary sources [31]. Alternatively, existing reviews can be used as a reference source for identifying or selecting primary studies [31]. A potential next stage of the rapid review approach presented here could include utilising the results of the searches, or data extraction, from a specified subset of included systematic reviews to inform a new synthesis of primary studies. This would enable a more in-depth evaluation of and ensure that any new pooled analysis is not biased by double counting of included studies, which could occur in a meta-analysis of meta-analyses [39].

In capturing the breadth of the literature, we inevitably lost depth and detail. Limiting inclusion to systematic reviews means some modifiable factors investigated by primary studies but not covered by a systematic review may have been missed. No findings were reported for 12/27 (44%) of the potentially modifiable factors identified from the mapping review. Potential modifiable factors may have been evaluated as part of a non-prognostic factor research question, published as a non-English language publication or conference abstract, or not deemed to be a systematic review. Some studies retrieved in full were found, on inspection, not to meet the inclusion criteria.

The challenge of defining modifiable factors was a potential limitation of our review. The decision on what constitutes a modifiable factor is fairly subjective, and in order to avoid excluding any potentially important modifiable factors, we chose to consider all those identified by either stakeholder. However, it could be argued that the list should have been limited to those that are modifiable once cancer has been diagnosed, thus excluding features of extensive disease which could, in theory, be modified but are unlikely to change patient outcomes. The list used in our review was based on limited input from only two clinicians. A more robust approach would have utilised expert consensus methodology, for example, using a Delphi survey; however, this was not feasible within the confines of the current project. Another potential limitation of our review is the timing of the searches, which were conducted at the end of 2015. However, our report was sufficient to meet the needs of our stakeholders, and since we present the review here as a methodological example, we did not update the searches.

An important limitation of the included reviews, and the available evidence base for the prognostic factors to inform clinical practice, was the limited data presented on histological subtypes. Many reviews focused on the evolution of lung cancer as a whole and only reported limited data on specific subtypes. Notably, three important and potentially useful modifiable factors were only considered by systematic reviews that evaluated lung cancer as a whole. Lung cancer is a heterogeneous disease, with different cell types growing and spreading differently. The feasibility of having any impact on the prognosis of lung cancer is dependent on tumour growth rate and spread of the disease, and as such, research studies lumping lung cancer types can be non-informative for implementation in clinical practice. Even lumping all NSCLC subtypes in one group can be non-informative as shown in systematic review of the natural history and growth rates of NSCLC, which found that the data regarding the distribution of tumour volume doubling time showed a wide spectrum of growth rates [40]. However, we also acknowledge that the broad remit of our review and the large number of included reviews meant that we have also reported data according to the broader categories of lung cancer types. Better reporting of the findings relating to histological subtypes is required, within both systematic reviews and primary studies.

Our review shows the large volume of systematic reviews that have already been conducted, indicating the need for an overview of current reviews, before yet another systematic review of primary studies is considered. Our review also shows that despite the enthusiasm for conducting systematic reviews of prognostic factors for lung cancer, very few focus on modifiable factors. A recent comprehensive analysis of global lung cancer research conducted over a decade (2004–2013) found that commitment to lung cancer research has fallen in most countries apart from China, and shows that it has no correlation with lung cancer burden [41]. Our findings corroborate this in that most of the reviews we identified were from China, yet interestingly, only one of these reviews evaluated a potentially modifiable factor (gross tumour volume), which could be argued to be a feature of extensive disease rather than a modifiable factor. Our review also highlights the limitations, in both volume and quality, of the primary studies evaluating modifiable factors.

The review identifies important gaps in the evidence base and potential areas for future, more in-depth, focused systematic reviews. Most of the included reviews focused on prognostic factors for NSCLC, and more reviews are likely to be needed for prognostic factors relating to SCLC. However, better reporting of histological subtypes in primary studies is required, in order to assist subsequent inclusion in future reviews. Further research is also needed to define modifiable factors. Future systematic reviews are likely needed for the 15 potentially modifiable factors for which no findings are reported in the current review.

## Conclusions

The study provides an example of the successful development and application of a novel approach to review the extensive and complicated research on prognostic factors to inform decision-making. It enabled us to develop a summary of the evidence base that is directly relevant to the stakeholders in an objective, repeatable, and transparent way. It also provides essential information for future research. However, it also required extensive planning, management, and ongoing reviewer training, which was time-consuming.

The review identified a large volume of published systematic reviews and meta-analyses of prognostic and predictive factors for lung cancer survival. These provide evidence for a long list of prognostic factors, but interestingly, few were evaluated by multiple reviews. Where multiple reviews did evaluate the same prognostic factors, their findings, on the whole, were fairly consistent. The review identified several potentially modifiable factors for lung cancer survival, which could contribute to evidence-based initiatives to improve lung cancer survival.

## Additional files

Additional file 1: Appendix A. Search strategy. (DOCX 17 kb)

Additional file 2: Appendix B. Results of mapping review. **Table B1**. Summary of studies included in mapping review and their status in stage 2, overview of reviews. (DOCX 52 kb)

Additional file 3: Appendix C. Description of included reviews in stage 2, overview of reviews. **Table C1**. Summary of included reviews. **Table C2**. Reference details of included studies. (DOCX 93 kb)

Additional file 4: Appendix D. Results of reviews in stage 2, overview of reviews. **Table D1**. ‘New’ biomarkers or biological factors not used in routine practice. **Table D2**. Tumour characteristics. **Table D3**. Metabolic criteria. **Table D4**. Clinical characteristics or routinely assessed biological variables. **Table D5**. Patient characteristics. **Table D6**. Healthcare provider and system. **Table D7**. Prognostic factors classified as ‘other’. (DOCX 96 kb)

Additional file 5: Appendix E. Description and results of reviews that aimed to evaluate any prognostic factor. **Table E1**. Summary of included reviews that considered the inclusion of any prognostic factor. **Table E2**. Modifiable prognostic factors evaluated by reviews that investigated ‘any’ prognostic factor associated with survival. (DOCX 22 kb)

Additional file 6: Appendix F. List of modifiable prognostic factors. **Table F1**: Modifiable prognostic factors evaluated by included reviews. (DOCX 14 kb)

Additional file 7: Appendix G. Quality appraisal of reviews investigating modifiable factors using a modified AMSTAR checklist. AMSTAR questions. **Table G1**. Quality appraisal of included reviews assessing modifiable factors. (DOCX 18 kb)

Additional file 8: Appendix H. Summary of magnitude of effect for modifiable factors. **Table H1**. Summary effect estimates based on pooled data. **Table H2**. Summary effect estimates from individual studies included in reviews reporting narrative synthesis. (DOCX 23 kb)
