# Supplementary material for: Pragmatic methods for reviewing exceptionally large bodies of evidence: systematic mapping review and overview of systematic reviews using lung cancer survival as an exemplar
Source: Syst Rev. 2019 Jul 16;8:171. doi: 10.1186/s13643-019-1087-4 (PMC6631880; doi:10.1186/s13643-019-1087-4)
Supplement: Supplementary file 1 — Appendix A. Search strategy. (DOCX 17 kb) [file 13643_2019_1087_MOESM1_ESM.docx]

APPENDIX A: SEARCH STRATEGY

|  | **Lung Cancer terms** |
| --- | --- |
| 1 | exp Lung Neoplasms/ |
| 2 | (lung$ adj5 (cancer$ or carcin$ or tumor$ or tumour$ or neoplasm$)).tw. |
| 3 | ((carcinoma or neoplasia or neoplasm$ or adenocarcinoma or cancer$ or tumor$ or tumour$ or malignan$) adj3 lung$).tw. |
| 4 | Or/1-3 |
|  | **Prognostic factors terms** |
| 5 | prognostic methods.mp. |
| 6 | predictive factors.mp. |
| 7 | (prognos$ adj10 (relapse$ or recurrence$ or survival$ or death$ or mortality or progress$ or disease free$)).ti,ab. |
| 8 | (predict$ adj10 (relapse$ or recurrence$ or survival$ or death$ or mortality or progress$ or disease free$)).ti,ab. |
| 9 | (neural network$ adj10 (relapse$ or recurrence$ or survival$ or death$ or mortality or progress$ or disease free$)).ti,ab. |
| 10 | survival rate/ |
| 11 | exp prognosis/and (relapse$ or recurrence$ or survival$ or death$ or mortality or progress$ or disease free$).ti,ab. |
| 12 | disease free survival/ |
| 13 | mortality/ |
| 14 | recurrence/ |
| 15 | neural networks computer/and (relapse$ or recurrence$ or survival$ or death$ or mortality or progress$ or disease free$).ti,ab. |
| 16 | exp models statistical/and (relapse$ or recurrence$ or survival$ or death$ or mortality or rogress$ or disease free$).ti,ab. |
| 17 | algorithms/and (relapse$ or recurrence$ or survival$ or death$ or mortality or progress$ or disease free$).ti,ab |
| 18 | (algorithm$ adj10 (relapse$ or recurrence$ or survival$ or death$ or mortality or progress$ or disease free$)).ti,ab. |
| 19 | exp survival analysis/ |
| 20 | nomogram$.mp. |
| 21 | ((marker$ or biomarker$) adj10 (prognos$ or predict$)).mp. |
| 22 | ((Incidence$ or diagnosed$ or first episode$) adj10 (prognos$ or predict$)).mp. |
| 23 | ((Course$ or natural history$ or long term$) adj10 (prognos$ or predict$)).mp. |
| 24 | Or/5-23 |
|  | **Socio-economic factors terms** |
| 25 | Social Class/ or Socio-economic Factors/ |
| 26 | Socio-economic status.ti,ab. |
| 27 | Education/ or exp Education, Continuing |
| 28 | Income/cl,sn |
| 29 | Exp Health Status/sn,td |
| 30 | Exp Poverty/pc, sn, td |
| 31 | Exp Social Class/ |
| 32 | Socio-economic position.ti,ab. |
| 33 | Inequalities.ti,ab. |
| 34 | Exp Social Environment/td |
| 35 | Social Factors.ti,ab. |
| 36 | Income.ti,ab. |
| 37 | Exp Residence Characteristics/cl, sn |
| 38 | Social Class.ti,ab. |
| 39 | Education.ti,ab. |
| 40 | Exp Health Status Disparities/ |
| 41 | Inequities.ti,ab. |
| 42 | Deprivation.ti,ab. |
| 43 | Equity.ti,ab. |
| 44 | Inequity.ti,ab. |
| 45 | Insurance status.ti,ab. |
| 46 | Or/25-45 |
| 47 | (progno$ or predict$ or relapse$ or recurrance$ or survival$ or death$ or mortality$ or progress$ or disease free$ stage$ or treatment$).ti,ab. |
| 48 | 46 and 47 |
|  | **Systematic review terms** |
| 49 | meta-analysis.pt |
| 50 | meta-analysis/ or systematic review/ or meta-analysis as topic/ or "meta analysis (topic)"/ or "systematic review (topic)"/ or exp technology assessment, biomedical/ |
| 51 | ((systematic* adj3 (review* or overview*)) or (methodologic* adj3 (review* or overview*))).ti,ab. |
| 52 | ((quantitative adj3 (review* or overview* or synthes*)) or (research adj3 (integrati* or overview*))).ti,ab. |
| 53 | ((integrative adj3 (review* or overview*)) or (collaborative adj3 (review* or overview*)) or (pool* adj3 analy*)).ti,ab. |
| 54 | (data synthes* or data extraction* or data abstraction*).ti,ab. |
| 55 | (handsearch* or hand search*).ti,ab. |
| 56 | (mantel haenszel or peto or der simonian or dersimonian or fixed effect* or latin square*).ti,ab. |
| 57 | (met analy* or metanaly* or technology assessment* or HTA or HTAs or technology overview* or technology appraisal*).ti,ab. |
| 58 | (meta regression* or metaregression*).ti,ab. |
| 59 | (meta-analy* or metaanaly* or systematic review* or biomedical technology assessment* or bio-medical technology assessment*).mp,hw. |
| 60 | (medline or cochrane or pubmed or medlars or embase or cinahl).ti,ab,hw. |
| 61 | (cochrane or (health adj2 technology assessment) or evidence report).jw. |
| 62 | (outcomes research or relative effectiveness).ti,ab. |
| 63 | Or/49-62 |
| 64 | 4 and 63 |
| 65 | 24 or 48 |
| 66 | 64 and 65 |
|  | **Limits** |
| 67 | (animal or cell line$ or vitro or invitro or rat or rats or mouse or mice).ti,ab. |
| 68 | letter.pt. |
| 69 | comment.pt. |
| 70 | Or/67-69 |
| 71 | 66 not 70 |
| 72 | Limit 71 to year 1990-Current |
