# Supplementary material for: Pragmatic methods for reviewing exceptionally large bodies of evidence: systematic mapping review and overview of systematic reviews using lung cancer survival as an exemplar
Source: Syst Rev. 2019 Jul 16;8:171. doi: 10.1186/s13643-019-1087-4 (PMC6631880; doi:10.1186/s13643-019-1087-4)
Supplement: Supplementary file 2 — Appendix B. Results of mapping review. Table B1. Summary of studies included in mapping review and their status in stage 2, overview of reviews. (DOCX 52 kb) [file 13643_2019_1087_MOESM2_ESM.docx]

**APPENDIX B: RESULTS OF MAPPING REVIEW**

**TABLE B1: Summary of studies included in mapping review and their status in stage 2, overview of reviews**

| **Author, year** | **Ref ID** | **Prognostic factors evaluated** | **Lung cancer** | **Treatment** | **No. of PFs investigated** | **Theme** | **Status at stage 2 based on full articles (and inclusion in overview of reviews)** |
| --- | --- | --- | --- | --- | --- | --- | --- |
| Abdel Rahmen | 55 | Skin rash | mxdC LC | Cetuximab | S | 4 |  |
| Aboshi, 2014 | 182 | age, histology | NSCLC (adv) |  | S (2) | 2,4 | INCLUDE |
| Albain, 1990 | 1244 | LDH, stage (ext dis), PS | SCLC |  | M | 2 | MA only |
| Albain, 1991 | 1239 | PS, gen, age (<70 years), ethnicity, LDH | NSCLC |  | M | 2 | MA only |
| Albain, 2009 | 730 | Stage, ethnicity (African-American) | mxdC LC |  | M | 2 | MA only |
| Al-Saleh, 2012 | 6981 | histology | NSCLC (adv) | Platinum-based CTX (pemetrexed) | S | 4 |  |
| Ashworth, 2013 | 237 | treatment of the primary tumour, N-stage, DFI (> 6mths) | NSCLC (met) | included pts who had received surgery (+/-RT) or RT for primary T | M | 2 | Theme 1,2; INCLUDE |
| Ashworth, 2014 | 105 | Any PF (node, sync mets) | NSCLC (met) | included pts who had received surgery (+/-RT) or RT for primary T | M | 1, 2 | Theme 1,2,3; INCLUDE |
| Askoxylakis, 2010 | 674 | None | mxdC LC |  | NA | 1 |  |
| Bai, 2014 | 156 | S100A4 | LC |  | S | 2 | Full text unavailable |
| Ball, 2010 | 7346 | histology | LC (non-met) | Accelerated or hyperfractionated RT | S | 4 |  |
| Bao, 2014 | 6081 | stage, T size | LC (early) | segmentectomy vs lobectomy | S (2) | 4 |  |
| Barni, 2013 | 6463 | Ethnicity (Asian) | NSCLC (adv mut) | EGFR TKIs (gefitinib and erlotinib) | S | 4 |  |
| Behera, 2014 | 5815 | stage (AIS and MIA) | ADC |  | S | 2 | INCLUDE |
| Bennani-Baiti, 2009 | 7498 | CRP | mxdC LC |  | S | 2 | Conference abstract |
| Berghmans, 2006 | 886 | TTF-1 | NSCLC |  | S | 2 | INCLUDE |
| Berghmans, 2008 | 805 | SUVMax (FDG-PET) | NSCLC |  | S | 2,4 | Theme 2 only; INCLUDE |
| Berghmans, 2011 | 8434 | Any [SUVMax (FDG-PET), biomarkers, PS, stage (IIIA, IIIB), age, weight loss, response to treatment, characteristics describing the locoregional extension of T] | NSCLC (III) |  | M | 2 | INCLUDE |
| Bianchi, 2007 | 811 | gene signatures (10-gene predictive model) | LADC (I) |  | M | 3 |  |
| Biaoxue, 2012 | 6836 | gen, smoking status, histology, skin rash | NSCLC | EGFR TKI (gefitinib) | M | 4 |  |
| Botling, 2013 | 344 | multiple genes (including CADM1) | NSCLC |  | M | 2,3 | MA only |
| Brambilla, 2010 | 7354 | histology, lymphocytic invasion status | NSCLC | platinum based CXT | S (2) | 2,3,4 | Conference abstract |
| Breen, 2008 | 791 | ERCC1 | NSCLC (surR) |  | S | 2,4 | INCLUDE |
| Bremnes, 2002 | 1061 | E-cadherin | LC |  | S | 2 | Did not meet review criteria |
| Bria, 2010 | 7355 | gen, smoking status, EGFR subtypes | NSCLC (adv mut) | EGFR TKI (gefitinib) | M | 2,4 | Conference abstract |
| Bria, 2011 | 496 | gen, smoking status | NSCLC (adv mut) | EGFR TKI | S (2) | 2,4 | Theme 4 only |
| Brown, 2013 | 271 | EGFR | NSCLC (adv) | CXT | S | 1,2,4 | Treatment effectiveness only |
| Brundage, 2002 | 1051 | Any PF (169 investigated) | NSCLC (III) |  | M | 2 | INCLUDE |
| Burdett, 2014 | 5940 | age, gender, PS, histology, stage | NSCLC (serR) | pre-op CTX | M | 2,4 | Theme 4 only |
| Burdett, 2015 | 5554 | age, gen, histology, stage, PS | NSCLC (serR) | adj CTX | M | 4 |  |
| Buttigliero, 2011 | 494 | VitD level, VDR | LC |  | S (2) | 2 | INCLUDE |
| Campbell, 2010 | 663 | EGFR, smoking status, histology | NSCLC | EGFR TKI (gefitinib) | M | 2,4 | Theme 4 only |
| Cao, 2015 | 5674 | T size, T loc, histology, GGO | NSCLC | sublobar resections versus lobectomy | M | 4 |  |
| Carlson, 2009 | 720 | EGFR | NSCLC (mut) | EGFR TKIs (gefitinib and erlotinib) | S | 2,4 | Theme 2 only; INCLUDE |
| Caro, 2001 | 1086 | Anaemia | LC |  | S | 2 | LC only |
| Carter, 2014 | 5362 | Non-genetic factors | NSCLC |  | M | 2,4 | INCLUDE |
| Chaimani, 2011 | 6449 | EGFR gene gain, WT tumours, ethnicity (Asian), stage | NSCLC (untreated) |  | M | 2 | Conference abstract |
| Chang, 2015 | 8431 | Cav-1 | NSCLC |  | M | 3,4 |  |
| Chang, 2014 | 6039 | FGR gene amplification | MxdC-LC |  | S(2) | 2 | LC only |
| Chen, 2010 | 635 | ERCC1 | NSCLC (adv) | platinum-based CTX | S | 2,4 | INCLUDE |
| Chen, 2013 | 257 | SOX-2 | NSCLC |  | S | 2 | INCLUDE |
| Chen, 2013 | 313 | stage, gen, ethnicity (Asian), PS, smoking status, histology, EGFR, previous response to CTX | NSCLC (adv) | EGFR TKIs (gefitinib and erlotinib) | M | 4 |  |
| Chen, 2013 | 6663 | microRNA-SNPs | LC |  | M | 2 | Theme 2,3 |
| Chen, 2014 | 86 | Cav-1, histology | NSCLC |  | S (2) | 2 | INCLUDE |
| Chen, 2015 | 54 | Ethnicity (Asian) | NSCLC | Bevacizumab | S | 4 |  |
| Chen, 2015 | 5566 | ILD | NSCLC | CTX | S | 4 |  |
| Chen, 2015 | 5619 | Stage | NSCLC (early) | adj CTX without RT | S | 4 |  |
| Chen, 2015 | 5672 | K-RAS and/or PIK3CA | NSCLC | EGFR TKIs | S (2) | 4 |  |
| Chen, 2015 | 5751 | EZH2 | mxdC NSCLC |  | S | 2 | INCLUDE |
| Chida, 2008 | 773 | psychosocial factors (stress-related) | mxdC LC |  | M | 1,2 | LC only |
| Choma, 2001 | 1085 | Aneuploidy | NSCLC (surR) |  | S | 2 | INCLUDE |
| Christopoulos, 2013 | 5789 | TB | LC | CTX plus anti-TB treatment | S | 2 | LC only; INCLUDE (evaluating a relevant modifiable factor) |
| Chung, 2014 | 206 | Any biomarker | mxdC NSCLC |  | M | 1,2 | Did not meet review criteria |
| Clausen, 2014 | 5748 | SUV Max (FDG-PET) | mxdC NSCLC | RT | S | 2 | Conference abstract |
| Collins, 2013 | 6201 | sarcopenia | LC | CXT | S | 4 |  |
| Cui, 2012 | 451 | XRCC1 | LC | CXT | S | 2 | LC only |
| Dahabreh, 2011 | 535 | EGFR | NSCLC | EGFR TKIs | S | 4 |  |
| Dai, 2015 | 5508 | FOXM1 | mxdC NSCLC |  | S | 2 | INCLUDE |
| de Geus-Oei, 2007 | 817 | SUVMax (FDG-PET) | NSCLC |  | S | 2,4 | INCLUDE |
| Deghaidy, 2005 | 923 | age, stage (tumour and grade) | LC |  | M | 1,2,3 | Theme 2 only; INCLUDE |
| Delmotte, 2002 | 1042 | VEGF | LC |  | S | 2 | Non-English Language |
| Deng, 2015 | 5645 | BMM | NSCLC (surR) |  | S | 2 | INCLUDE |
| Des Guetz, 2012 | 5904 | EGFR, WT tumour | NSCLC | EGFR TKIs | S (2) | 2,4 | Theme 4 only |
| Di Maio, 2010 | 686 | gen, PS, histology, stage, previous treatment, previous response (previous use of platinum and response to first-line CXT) | NSCLC (adv) |  | M | 2,3 | MA only |
| Dimou, 2014 | 88 | MET, histology (ADC) | NSCLC (surR) |  | S (2) | 2 | INCLUDE |
| Ding, 2013 | 6220 | ethnicity (Asian), gen, age, PS, smoking history, histology, EGFR subtypes (Del19/L858R) | NSCLC (adv mut) | EGFR TKIs | M | 2,4 | Conference abstract |
| Ding, 2014 | 6188 | EGFR subtype (T790M) | NSCLC | EGFR TKIs | S | 4 |  |
| Dong, 2015 | 5679 | SUVmax (FDG-PET) | NSCLC (early) |  | S | 4 |  |
| Ellis, 2010 | 7352 | histology, biomarker testing (EGFR, K-RAS, ERCC1, BRCA, TUBB3I, P53, EML4/ALK) | NSCLC | Targeted therapy | M | 2,4 | INCLUDE |
| Ellis, 2015 | 5698 | EGFR | NSCLC (adv) | EGFR inhibitors | S | 4 |  |
| Fan, 2008 | 777 | Survivin | NSCLC (surR mainly) |  | S | 2 | INCLUDE |
| Fan, 2012 | 460 | Stage (I, IA) | NSCLC (I) | Sublobectomy versus lobectomy | S | 4 |  |
| Fan, 2014 | 5974 | EGFR | NSCLC (met) | EGFR-TKI therapy | S | 4 |  |
| Florou, 2014 | 51 | Smoking status | mxdC NSCLC |  | S | 2,4 | INCLUDE |
| Fu, 2014 | 170 | VEGF, stage, histology | NSCLC | RT | M | 2,4 | Full text unavailable |
| Gao, 2013 | 6223 | Sync mets, histology, ipsilateral adrenal mets | NSCLC (met surR) |  | M | 4 |  |
| Garassino, 2009 | 731 | K-RAS, smoking status | NSCLC (adv) |  | S (2) | 4 |  |
| Gkogkou, 2014 | 5026 | Necrosis, apoptotic index | NSCLC |  | S (2) | 2 | Not a SR |
| Gong, 2012 | 6996 | RRM1 | NSCLC (adv) | gemcitabine-containing CTX | S | 4 |  |
| Goulart, 2009 | 7499 | QoL | NSCLC (adv) | CTX | S | 1,4 |  |
| Griffiths, 2011 | 7135 | None | LC |  | NA | 1 |  |
| Grills, 2011 | 537 | VDAC1, gene signatures (6-gene signature) | NSCLC (early) |  | M | 3 |  |
| Guha, 2011 | 7013 | Occupation-painter, smoking status | LC |  | S (2) | 2 | Risk factor, not prognostic factor |
| Guo, 2014 | 134 | MET, ethnicity (Asian), histology | NSCLC (surR) |  | M | 2 | INCLUDE |
| Gupta, 2010 | 610 | Albumin | LC |  | S | 2,4 | Theme 2 only; INCLUDE |
| Gyorffy, 2013 | 220 | ERCC1, RAD51, CDKN2A, OPN, EZH2, ANXA3, ADAM28 | LC |  | M | 3 |  |
| Hamada, 2005 | 939 | stage, histology, ethnicity (Japanese) | NSCLC (surR) | Adj platinum-based CTX (tegafur-uracil) | Y | 4 |  |
| Hasegawa, 2015 | 39 | Smoking status | NSCLC (mut) | EGFR-TKI therapy vs platinum-based CTX | S | 4 |  |
| Hauser, 2006 | 892 | cachexia, dyspnoea, pain, QoL, PS, anaemia, thrombocytopenia, Albumin (hypoalbuminaemia), alk phos, LDH | mxdC LC |  | M | 2 | LC only |
| He, 2013 | 6625 | microRNA-155 | mxdC LC |  | S | 2 | INCLUDE |
| Hirsch, 2008 | 7515 | Histology | NSCLC |  | S | 2,4 | INCLUDE |
| Hu, 2013 | 286 | VEGFR | LC |  | S | 2 | INCLUDE |
| Huang, 2012 | 454 | cyclin E | LC |  | S | 2 | INCLUDE |
| Huang, 2013 | 343 | Survivin | NSCLC |  | S | 2 | INCLUDE |
| Huang, 2015 | 7 | BIM Polymorphism | NSCLC (adv mut) | EGFR-TKIs | S | 4 |  |
| Huang, 2015 | 95 | VPI | NSCLC (I) |  | S | 2 | INCLUDE |
| Hubner, 2011 | 483 | ERCC1 | NSCLC / SCLC | +/- Platinum-based CTX | S | 2,4 | INCLUDE |
| Huncharek, 1999 | 1133 | K-RAS | NSCLC (surR mainly) |  | S | 2 | INCLUDE |
| Huncharek, 2000 | 1121 | p-53 | NSCLC |  | S | 2 | INCLUDE |
| Huo, 2015 | 5784 | ALDH1 | LC |  | S | 2 | INCLUDE |
| Ibrahim, 2010 | 7225 | EGFR | NSCLC (adv) | EGFR TKIs (gefitinib) | S | 4 |  |
| Im, 2015 | 14 | MTV, TGL, SUVMax (FDG-PET) | NSCLC |  | S (2) | 2 | INCLUDE |
| Jiang, 2012 | 424 | ERCC1 | NSCLC (surR) | CTX | S | 2,4 | INCLUDE |
| Jiang, 2013 | 312 | Cox-2 | NSCLC |  | S | 2 | INCLUDE |
| Jiang, 2014 | 140 | CD44 or CD44 isoforms, CD44s or CD44v6 | NSCLC |  | M | 2 | INCLUDE |
| Jiang, 2014 | 144 | VEGF-C | NSCLC |  | S | 2 | INCLUDE |
| Jiang, 2015 | 5 | Sync T | MPLC (surR) |  | S | 2 | INCLUDE |
| Jiang, 2015 | 4951 | VPI | NSCLC (mut) |  | S | 2 | INCLUDE |
| Jiang, 2015 | 4988 | FGFRI | LSQCC |  | S | 2 | INCLUDE |
| Jin, 2014 | 67 | CRP | NSCLC |  | S | 2 | INCLUDE |
| Jing, 2015 | 5605 | CRP | NSCLC (SurR) |  | S | 2 | INCLUDE |
| Johnson, 2006 | 894 | TR, TTP | mxdC NSCLC |  | M | 4 |  |
| Jun, 2015 | 5563 | FGFR1 | LC |  | S | 2 | INCLUDE |
| Kiagia, 2012 | 6305 | Cachexia | LC |  | S | 2 | Conference abstract |
| Kiely, 2012 | 394 | PS, histology, TTP | NSCLC (adv) | CTX | S (2) | 1,2 | Theme 1 only |
| Kilvaer, 2015 | 5533 | LVD, VEGF-C | NSCLC (met) |  | S (2) | 2 | INCLUDE |
| Knez, 2011 | 525 | MDR1, MRP1, MRP2, MVP, topoisomerase II, ERCC1 | LSQCC | CTX | M | 2 | Theme 2,4; INCLUDE |
| Kong, 2014 | 157 | MDM2 | NSCLC |  | S | 2 | INCLUDE |
| Ku, 2011 | 476 | EGFR | NSCLC (adv) | EGFR TKIs (gefitinib) | S | 4 |  |
| Kuan, 2015 | 5439 | EGFR subtypes (Del19/L858R) | NSCLC (mut adv) | EGFR TKIs | S (2) | 4 |  |
| Labots, 2014 | 6105 | peptide gene profiling | mxdC NSCLC |  | M | 2,4 | Theme 4 only |
| Landre, 2015 | 5565 | EGFR, age | NSCLC | EGFR TKIs | S (2) | 4 |  |
| le Chevalier, 1996 | 1199 | age, gen, stage, histology, PS | NSCLC (adv) | CTX | M | 4 |  |
| Le Pechoux, 2011 | 6394 | Histology type (SCLC / NSCLC) | LC (non-met) | RT | S | 4 |  |
| Lee, 2012 | 5905 | EGFR subtypes | NSCLC (adv) | EGFR TKIs | S | 4 |  |
| Lee, 2014 | 186 | testing approach (EGFR), ethnicity, previous treatment | NSCLC (adv mut) |  | M | 4 |  |
| Lee, 2015 | 60 | EGFR subtypes (Del19 or L858R), smoking status, gen, PS, age, ethnicity, histology | NSCLC (adv) | EGFR TKIs | M | 4 |  |
| Lee, 2015 | 5578 | EGFR subtypes (Del19 or L858R), smoking status, gen, PS, age, ethnicity, histology, stage | NSCLC (III-IV) |  | M | 4 |  |
| Li, 2008 | 787 | PLC | LC (surR) |  | S | 2 | LC only |
| Li, 2012 | 428 | histology | NSCLC (adv) | platinum-based CTX | S | 4 |  |
| Li, 2013 | 272 | HIF1-alpha, HIF-2 alpha | NSCLC (surR) |  | S (2) | 2 | INCLUDE |
| Li, 2013 | 6217 | histology | NSCLC (adv) | anti-angiogenic TKIs | S | 4 |  |
| Li, 2014 | 100 | Histology | NSCLC (adv) | CTX plus targeted therapy | S | 4 |  |
| Li, 2014 | 5993 | EGFR, WT tumour | NSCLC (adv) | CTX plus EGFR TKIs | S (2) | 4 |  |
| Li, 2014 | 6075 | EGFR | NSCLC (adv) | EGFR TKIs | S | 4 |  |
| Li, 2015 | 4976 | mTOR, p-mTOR | NSCLC |  | S (2) | 2 | INCLUDE |
| Li, 2015 | 5436 | ESR1, ESR2 | NSCLC |  | S (2) | 2 | INCLUDE |
| Liang, 2014 | 183 | MMP-7 | NSCLC |  | S | 2 | INCLUDE |
| Liang, 2014 | 6014 | EGFR, ethnicity (Asian), testing approach | NSCLC (adv) | EGFR TKIs | S | 2,4 | Did not report overall survival |
| Liang, 2015 | 5562 | BRCA1, ethnicity (Asian), testing approach | mxdC NSCLC/SCLC | CTX | M | 4 |  |
| Liang, 2015 | 5708 | CXCR4 | LC |  | S | 2 | INCLUDE |
| Liao, 2014 | 76 | CIF (CRP, IL-6, IL-8, IL-10, TNF-alpha) | NSCLC | CXT/RT, surgery | M | 2 | INCLUDE |
| Lim, 2010 | 662 | PLC | NSCLC (sur) | Surgery | S | 2 | INCLUDE |
| Lima, 2011 | 6402 | geographical origin of study | SCLC (ext) | platinum-based CTX | S | 2,4 | Conference abstract |
| Lima, 2013 | 6593 | geographical origin of study | SCLC (ext) | platinum-based CTX | S | 4 |  |
| Liu, 2010 | 612 | HER-2 | LC |  | S | 2 | INCLUDE |
| Liu, 2013 | 239 | TS | NSCLC | pemetrexed-based CXT | S | 2,4 | INCLUDE |
| Liu, 2013 | 332 | skin rash | NSCLC | EGFR TKIs | S | 2,4 | INCLUDE |
| Liu, 2015 | 36 | TS | NSCLC | CXT | S | 2 | INCLUDE |
| Liu, 2015 | 5525 | OPN | NSCLC |  | S | 2 | INCLUDE |
| Liu, 2015 | 5574 | EGFR | NSCLC (met) | EGFR TKIs plus RT (mets) | S | 2,4 | Conference abstract |
| Liu, 2015 | 5677 | CXCR4 | NSCLC |  | S | 2,4 | Theme 2 only; INCLUDE |
| Liu, 2015 | 5715 | CAFs | mxdC LC |  | S | 2 | Conference abstract |
| Liu,2015 | 5680 | SUVMax (FDG-PET), MTV, TLG | NSCLC (surR) |  | S (2) | 2 | INCLUDE |
| Lou-Qian, 2013 | 334 | P16 | NSCLC |  | S | 2 | INCLUDE |
| Luan, 2014 | 171 | Blood transfusion | LC (surR) |  | S | 2 | INCLUDE |
| Luo, 2014 | 107 | CD44, CD44-V6 | NSCLC |  | S (2) | 2 | INCLUDE |
| Luo, 2015 | 58 | LRF, T stage, VPI, LVI | NSCLC (NI) |  | M | 2,4 | Theme 2 only; INCLUDE |
| Luo, 2015 | 5651 | ERbeta | NSCLC |  | S | 2 | INCLUDE |
| Lyman, 2013 | 6300 | Myelosuppression (CTX induced) | mxdC NSCLC (met) | CTX | S | 2 | Conference abstract |
| Ma, 2007 | 844 | stage (pN0, pN1, pN2) | NDSLC (early) | Surgery: sleeve lobectomy (SL) +/- pulmonary artery reconstruction (PAR) vs pneumonectomy (PN) | S | 4 |  |
| Ma, 2012 | 395 | microRNA-21 | NSCLC |  | S | 2 | INCLUDE |
| Ma, 2012 | 401 | CTCs | LC |  | S | 2 | INCLUDE |
| Ma, 2014 | 190 | Serum D-dimer level | LC |  | S | 2 | INCLUDE |
| Ma, 2014 | 6116 | gen, smoking status, EGFR, histology | NSCLC (adv) | EGFR TKIs (erlotinib) | M | 4 |  |
| Ma, 2015 | 5676 | BIM deletion polymorphism | NSCLC (mut) | EGFR TKIs | S | 2,4 | Theme 2 only; INCLUDE |
| Mandrekar, 2006 | 890 | Age, gen, PS, stage, BMI, creatinine level, Hb, WBC, Plt | NSCLC (adv) |  | M | 2,3 | MA only |
| Manser, 2005 | 964 | stage | NSCLC (early) | Surgery | S | 4 |  |
| Mao, 2015 | 64 | Blood instead of tumour tissue to detect EGFR mut | NSCLC | EGFR TKIs | M | 4 |  |
| Marchevsky, 2009 | 7465 | ITC, MM | NSCLC |  | S (2) | 2 | Conference abstract |
| Marchevsky, 2010 | 625 | ITC, MM | NSCLC |  | S (2) | 2,4 | Theme 2 only; INCLUDE |
| Martin, 2003 | 1024 | Bcl-2 | LC |  | S | 2 | INCLUDE |
| Martin, 2004 | 969 | Ki67 | LC |  | S | 2 | INCLUDE |
| Mascaux, 2005 | 961 | RAS, p21 | LC |  | S (2) | 2 | INCLUDE |
| Mascaux, 2006 | 899 | Cox-2 | LC |  | S | 2 | INCLUDE |
| Meert, 2002 | 1045 | EGFR | NSCLC |  | S | 2 | INCLUDE |
| Meert, 2002 | 1050 | MVD count | LC (surR) |  | S | 2 | INCLUDE |
| Meert, 2003 | 1019 | c-erbB-2 [HER-2neu] | LC |  | S | 2 | INCLUDE |
| Mei, 2013 | 319 | Beta-catenin | NSCLC |  | S | 2 | INCLUDE |
| Meng, 2013 | 287 | K-RAS | NSCLC |  | S | 2 | INCLUDE |
| Miao, 2012 | 6341 | GGO | SCLC (surR) |  | S | 2 | INCLUDE |
| Min Tun, 2012 | 6697 | GGO | NSCLC (adv) | EGFR TKIs | S | 4 |  |
| Mitchell, 2012 | 421 | Smoking history | NSCLC (adv) | Any treatment including EGFR TKIs | S | 4 |  |
| Mitsudomi, 2000 | 1110 | P53 | NSCLC (surR) |  | S | 2 | INCLUDE |
| Mo, 2015 | 5602 | T status, N status | NSCLC |  | S (2) | 3,4 |  |
| Mohan, 2009 | 5163 | MDR | LC | CTX | S | 4 |  |
| Mollberg, 2014 | 196 | LVI | NSCLC (I) |  | S | 2 | INCLUDE |
| Montazeri, 2009 | 703 | Health-related quality of life | MxdC-LC |  | S | 1,2 | Theme 2 only; INCLUDE |
| Muller, 2011 | 546 | gene signatures ('60 potential biomarkers'; none significant) | NSCLC |  | M | 3 |  |
| Muralidarin, 2011 | 484 | unplanned circulatory bypass | NSCLC |  | S | 4 |  |
| Murphy, 2011 | 5331 | Previous treatment (platinum, relapsed) | NSCLC | Erlotinib or gefitinib | S | 4 |  |
| Murray, 2010 | 7299 | EGFR, EGFR subtypes, WT tumours | NSCLC | EGFR TKIs (gefitinib and erlotinib) | M | 2,4 | Conference abstract |
| Na, 2014 | 133 | SUV(Max) [pre-RT and post-RT] | NSCLC | RT | S | 2 | INCLUDE |
| Nair, 2009 | 706 | FDG uptake | NSCLC (I) |  | S | 2 | INCLUDE |
| Nakamura, 2005 | 952 | HER-2, histology | NSCLC |  | S (2) | 2 | INCLUDE |
| Nakamura, 2006 | 918 | EGFR | NSCLC |  | S | 2 | INCLUDE |
| Nakamura, 2011 | 481 | gen | NSCLC |  | S | 2 | INCLUDE |
| Neal, 2015 | 8441 | Timeliness of care | MxdC-LC |  | S | 2 | INCLUDE |
| Nie, 2015 | 4956 | BIM deletion polymorphism | NSCLC | EGFR-TKIs | S | 4 |  |
| Oberheim, 2013 | 6310 | histology, EGFR | mxdC LC (met) |  | S (2) | 2 | Conference abstract |
| Olsson, 2009 | 722 | timeliness of care | LC |  | S | 2 | LC only; INCLUDE (evaluating a relevant modifiable factor) |
| Ortega-Parra, 2013 | 6596 | skin rash, vomiting, neutropenia (all CTX induced) | NSCLC | EGFR inhibitors | M | 2,4 | Conference abstract |
| Owonikoko, 2010 | 7332 | refractory or sensitive to previous treatment | SCLC | CTX | S | 4 |  |
| Owonikoko, 2012 | 410 | refractory or sensitive to previous treatment | SCLC |  | S | 4 |  |
| Paesmans, 2010 | 667 | SUVMax (FDG-PET) | NSCLC |  | S | 2 | INCLUDE |
| Paesmans, 2013 | 6233 | SUV, stage (I-III), age, T size, surgery | NSCLC |  | M | 2 | Conference abstract |
| Pallis, 2011 | 482 | Age | NSCL (adv) | CTX | S | 2,4 | MA only |
| Pan, 2015 | 5774 | PD-L1 | NSCLC |  | S | 2 | INCLUDE |
| Parsons, 2010 | 695 | Smoking status | NSCLC (early) |  | S | 2,3 | Theme 1,2; INCLUDE |
| Paz-Ares, 2012 | 5906 | EGFR | NSCLC | EGFR TKIs | S | 2,4 | Conference abstract |
| Paz-Ares, 2014 | 5814 | Age | NSCLC (adv) | CTX: pemetrexed | S | 4 |  |
| Peng, 2012 | 437 | MMP-9 | NSCLC |  | S | 2 | INCLUDE |
| Peng, 2014 | 5929 | OPN (PSO, TTO) | LC |  | S (2) | 2 | INCLUDE |
| Peng, 2015 | 5752 | NLR | NSCLC |  | S | 2,4 | INCLUDE |
| Petrelli, 2012 | 389 | skin rash | NSCLC | EGFR TKIs | S | 2,4 | INCLUDE |
| Petrelli, 2015 | 5700 | LDH | mxdC LC |  | S | 2 | LC only |
| Pignon, 2008 | 775 | PS, stage, histology, gen, age, surgery (type of surgery) | NSCLC | Adj platinum-based CTX (Cisplatin) | M | 4 |  |
| Pilotto, 2014 | 6002 | Ethnicity (Asian) | NSCLC (mut adv) | EGFR TKIs | S | 4 |  |
| Pirker, 2010 | 7339 | p27, p16, cyclin E | NSCLC (SurR) | Adj platinum-based CTX | M | 2,3,4 | Conference abstract |
| Pochesci, 2013 | 6548 | EGFR, ethnicity (Asian) | NSCLC (met) | EGFR TKIs | S (2) | 4 |  |
| Popat, 2014 | 5996 | EGFR subtypes (Del19 and L858R) | NSCLC (mut) | EGFR TKIs | S (2) | 4 |  |
| Prades, 2015 | 5807 | MDT | mxdC LC |  | S | 2 | INCLUDE |
| Pu, 2011 | 6970 | gene signatures (SNPs in inflammation-related genes) | NSCLC |  | M | 3 |  |
| Pujol, 2004 | 993 | CYFRA 21-1 | NSCLC |  | S | 2 | INCLUDE |
| Qi, 2009 | 587 | QoL | NSCLC (adv) |  | S | 2 | MA only |
| Qi, 2012 | 359 | Ethnicity (Asian) | NSCLC | EFGR-TKIs: gefitinib or erlotinib | S | 4 |  |
| Qian, 2010 | 657 | MMP-2 | NSCLC |  | S | 2 | INCLUDE |
| Qin, 2013 | 229 | XPD polymorphism, ethnicity (Asian) | NSCLC | Platinum based CTX | S (2) | 2,4 | INCLUDE |
| Qiu, 2013 | 137 | XPD polymorphisms, ethnicity (Asian) | NSCLC | Platinum based CTX | S (2) | 4 |  |
| Qiu, 2013 | 221 | P-AKT expression | NSCLC |  |  | 2 | INCLUDE |
| Qiu, 2013 | 242 | XRCC3 polymorphism | NSCLC (adv) | Platinum based CTX | S | 2,4 | Theme 2 only; INCLUDE |
| Qiu, 2015 | 5592 | E-cadherin | NSCLC |  | S | 2 | INCLUDE |
| Qu, 2013 | 233 | CD133 | NSCLC |  | S | 2 | INCLUDE |
| Quinton, 2011 | 6781 | histology, EGFR, ERCC1, BRCA, Beta tubulin III, RRM1, K-RAS, or TP-53, EML4/ALK translocations | NSCLC | Targeted therapy | M | 2,4 | INCLUDE |
| Reiman, 2012 | 466 | TUBB3 | NSCLA (surR) | Adj CXT | S | 2,3,4 | MA only |
| Ren, 2013 | 249 | HIF-1alpha | LC |  | S | 2 | INCLUDE |
| Riley, 2015 | 5692 | Any | LC |  | M | 2 | Theme 3 |
| Rossi, 2011 | 6956 | skin rash, vomiting, neutropenia (CTX induced) | NSCLC | EGFR inhibitors | M | 4 |  |
| Roth, 2011 | 477 | ERCC1 | NSCLC | Platinum-based CTX | S | 2,4 | INCLUDE |
| Salah, 2012 | 467 | Age, gen, histology, stage (T and N), synchronous, visceral metastasis, perioperative CTX | NSCLC (SurR met) | metastatectomy | M | 2,4 | Theme 2 only; INCLUDE |
| Sarkar, 2012 | 6686 | Diabetes | LC |  | S | 2 | Conference abstract |
| Saso, 2012 | 427 | PLC | NSCLC (I SurR) |  | S | 2 | INCLUDE |
| Sastry, 2014 | 5876 | stage, node status (TN) and R0 resection, DFI (long), isolated mets | NSCLC (met surR) | Adrenalectomy (vs CXT/RT) for isolated metastasis | M | 4 |  |
| Saunders, 2010 | 7327 | Histology | LC | modified RT | S | 4 |  |
| Schild, 2011 | 6955 | stage (limited vs extensive SCLC), age, PS, gen, number of metastatic sites overall | SCLC | RT (Prophylactic cranial irradiation, PCI) | M | 4 |  |
| Schrijvers, 1994 | 1221 | SES | mxdC LC |  | S | 2 | LC only |
| Sebastian, 2014 | 5782 | EGFR subtypes (Del19/L858R) | NSCLC (adv mut) | EGFR TKIs (afatinib) | S (2) | 4 |  |
| Shao, 2014 | 52 | Bmi-1 | mxdC LC |  | S | 2 | LC only |
| Shao, 2014 | 122 | microRNA-375 | mxdC NSCLC |  | S | 2 | INCLUDE |
| Shao, 2015 | 5618 | SOX-2 | NSCLC |  | S | 2 | INCLUDE |
| Shen, 2013 | 266 | XRCC3 polymorphism | NSCLC (adv) | platinum-based CTX | S | 2,4 | Theme 2 only; INCLUDE |
| Shen, 2014 | 62 | EphA2 | mxdC LC |  | S | 2 | INCLUDE |
| Sheng, 2015 | 6 | histology, no previous treatment | NSCLC (adv) | EGFR plus CTX | S (2) | 4 |  |
| Sheng, 2015 | 5479 | histology, smoking status, gen | NSCLC (adv) | Targeted therapy plus CTX | M | 2,4 | Theme 4 only |
| Shepherd, 2013 | 288 | KRAS, KRAS subtypes (codon), WT tumours | NSCLC (early) | Adj CTX | M | 2,4 | MA only |
| Shrotriya, 2014 | 6124 | CRP | mxdC LC |  | S | 2 | Conference abstract |
| Shukuya, 2011 | 5714 | Histology | NSCLC (mut adv) | EGFR TKIs (gefitinib) | S | 4 |  |
| Siddiqui, 2010 | 666 | gen, ethnicity, MS | NSCLC (non-op) | RT | M | 2 | MA only |
| Slatore, 2010 | 621 | insurance status | LC |  | S | 2 | INCLUDE |
| Soo, 2011 | 520 | Ethnicity (Asian) | NSCLC (adv) | CTX | S | 2,4 | INCLUDE |
| Soria, 2013 | 350 | histology, wt loss | NSCLC (adv) | CTX plus bevacizumab | S (2) | 4 |  |
| Soria,2011 | 6383 | EGFR | LADC (mut) | platinum-based CTX | S | 2,4 | Conference abstract |
| Standfield, 2011 | 7043 | histology | NSCLC (adv) | CTX | S | 2,4 | Theme 4 only |
| Steels, 2001 | 5212 | P53 | LC |  | S | 2 | INCLUDE |
| Sun, 2013 | 6459 | survivin | NSCLC |  | S | 2 | INCLUDE |
| Sun, 2014 | 92 | serum-based proteomic test | NSCLC | EGFR-TKIs | S | 4 |  |
| Sun, 2015 | 5469 | LNR | NSCLC |  | S | 2 | INCLUDE |
| Tan, 2015 | 5517 | PS, EGFR, histology, previous treatment response (induction) | NSCLC (adv) | maintenance treatments | M | 4 |  |
| Tang, 2009 | 7452 | NSE | SCLC |  | S | 2 | Non-English Language |
| Tanvetyanon, 2008 | 799 | sync mets | NSCLC (sync) | Surgery (adrenalectomy) | S | 1,2 | INCLUDE |
| Tanvetyanon, 2013 | 320 | gen, age, stage (N status), T loc (unilateral), histology | NSCLC (sync) | Surgery (sync) | M | 2 | INCLUDE |
| Tanvetyanon, 2015 | 23 | histology (ADC), stage (N status), T size, T loc (bilateral cancers), age, gen | NSCLC (sync) | Surgery (sync) | M | 2,3 | INCLUDE |
| Tian, 2015 | 4978 | MALAT1 | mxdC LC |  | S | 2 | INCLUDE |
| Tong, 2011 | 486 | p16 | NSCLC |  | S | 2 | INCLUDE |
| Toy, 2003 | 1030 | PS | NSCLC | Palliative RT | S | 4 |  |
| Travis, 2011 | 541 | stage | LADC |  | S | 2 | Tumour classification not prognostic factor |
| Trivella, 2007 | 846 | MVD | NSCLC (<IV) |  | S | 2 | INCLUDE |
| Tsao, 2010 | 7359 | KRAS, wild type tumours, histology | NSCLC (surR) | ADJ platinum-based CTX | S | 2,4 | Conference abstract |
| Tsao, 2015 | 5505 | histology (subtype) | LADC (surR) | Adj CTX | S | 4 |  |
| Tsuboi, 2009 | 7486 | age, sex, or histology | NSCLC (IA) | Adj CTX (Tegafur-uracil) | S | 4 |  |
| Tsutani, 2014 | 6059 | LVI | NSCLC (early) |  | S | 2 | Did not meet review criteria |
| Tu, 2014 | 37 | VEGF subtypes (rs833061, rs699947) | LC |  | S (2) | 2 | LC only |
| Tun, 2012 | 6804 | EGFR | NSCLC (adv) | EGFR TKIs (erlotinib) | S | 4 |  |
| Vale, 2015 | 5754 | EGFR status, WT tumour | NSCLC (adv) | EGFR TKIs | S (2) | 4 |  |
| Vansteenkiste, 2004 | 982 | SUVMax (FDG-PET) | LC (NSCLC) |  | S | 2 | INCLUDE |
| Von Meyenfeldt, 2012 | 414 | Procedural volume, surgeon | LC (suR) |  | S(2) | 2 | LC only; INCLUDE (evaluating a relevant modifiable factor) |
| Wang, 2009 | 7384 | EGFR | NSCLC |  | S | 2,4 | Non-English Language |
| Wang, 2011 | 469 | Blood vessel invasion | NSCLC |  | S | 2 | INCLUDE |
| Wang, 2011 | 534 | RASSF1A methylation | NSCLC |  | S | 2 | INCLUDE |
| Wang, 2012 | 356 | LVI | NSCLC |  | S | 2 | INCLUDE |
| Wang, 2012 | 371 | MMP-2 | LC |  | S | 2 | LC only |
| Wang, 2012 | 403 | EGFR, histology (ADC) | NSCLC (adv) | EGFR TKIs (gefitinib) | S (2) | 4 |  |
| Wang, 2012 | 448 | LVD | NSCLC |  | S | 2 | INCLUDE |
| Wang, 2013 | 234 | CTCs | NSCLC |  | S | 2 | INCLUDE |
| Wang, 2013 | 247 | TS | NSCLC (adv) | pemetrexed-containing CTX | S | 2 | INCLUDE |
| Wang, 2013 | 268 | microRNA-21, microRNA-155 | NSCLC |  | S (2) | 2 | INCLUDE |
| Wang, 2014 | 129 | EGFR subtypes (Del19/L858R) | NSCLC (adv) | EGFR TKIs | S (2) | 2,4 | Theme 2 only; INCLUDE |
| Wang, 2014 | 150 | CEA | NSCLC (surR) |  | S | 2 | INCLUDE |
| Wang, 2014 | 151 | CD133 | NSCLC |  | S | 2 | INCLUDE |
| Wang, 2014 | 180 | Blood transfusion | LC (surR) |  | S | 2,4 | INCLUDE |
| Wang, 2014 | 187 | HIF-1alpha | NSCLC |  | S | 2 | INCLUDE |
| Wang, 2014 | 191 | TS | NSCLC | pemetrexed-containing CTX | S | 2,4 | INCLUDE |
| Wang, 2014 | 5514 | T-cadherin | NSCLC |  | S | 2 | INCLUDE |
| Wang, 2015 | 103 | PD-L1 | NSCLC |  | S | 2 | INCLUDE |
| Wang, 2015 | 5537 | microRNA-155 | NSCLC |  | S | 2 | INCLUDE |
| Wao | 326 | No treatment | NSCLC |  | S | 1 |  |
| Wei, 2011 | 499 | ERCC1 polymorphisms | NSCLC (adv) | platinum-based CTX | M | 4 |  |
| Wei, 2015 | 4957 | ALDH1 | LC |  | S | 2 | INCLUDE |
| Wen, 2015 | 5684 | KI67 | NSCLC (early) |  | S | 2 | INCLUDE |
| Werner-Wasik, 1999 | 8268 | weight loss, PS, stage (N status), CTX | NSCLC | RT +/- CTX | M | 2,4 | MA only |
| Wheatley-Price, 2010 | 629 | gen, histology | NSCLC (adv) | CTX | S (2) | 2,4 | MA only |
| Wheatley-Price, 2010 | 692 | gen | SCLC | CTX | S | 2,4 | MA only |
| Wheatley-Price, 2013 | 6228 | age | SCLC | CTX | S | 2,4 | Conference abstract |
| Wilson, 2011 | 6448 | VDAC2 | NSCLC |  | S | 3 |  |
| Wu, 2007 | 848 | EGFR, WT tumour, histology, smoking status | NSCLC | EGFR TKIs (gefitinib) | M | 2,4 | Theme 2 only; INCLUDE |
| Wu, 2011 | 523 | gene signatures (multiple SNPs) | NSCLC | platinum-based CTX | M | 2,3,4 | MA only |
| Wu, 2012 | 390 | E-cadherin | NSCLC |  | S | 2 | INCLUDE |
| Wu, 2012 | 400 | XRCC1 | NSCLC (adv) | platinum-based CTX | S | 2,4 | Theme 2 only; INCLUDE |
| Wu, 2013 | 280 | gene signatures (multiple gene expression and variants) | NSCLC (non-smokers) |  | M | 2,3 | MA only |
| Wu, 2014 | 30 | CD133 | NSCLC |  | S | 2 | INCLUDE |
| Wu, 2014 | 125 | MT1-MMP | mxdC LC |  | S | 2 | INCLUDE |
| Wu, 2015 | 5510 | NF-kappaB | mxdC NSCLC |  | S | 2 | INCLUDE |
| Xia, 2014 | 78 | microRNA polymorphisms | mxdC NSCLC |  | M | 2 | INCLUDE |
| Xia, 2014 | 172 | let-7 | mxdC LC |  | S | 2 | LC only |
| Xie, 2012 | 368 | survivin | NSCLC |  | S | 2 | INCLUDE |
| Xing, 2013 | 5058 | p16 | mxdC NSCLC |  | S | 2 | INCLUDE |
| Xu, 2011 | 6439 | EGFR, gen, smoking, ethnicity (Asian) | NSCLC (adv) | EGFR TKIs | M | 2,4 | Conference abstract |
| Xu, 2013 | 148 | MicroRNA-15 | mxdC NSCLC |  | S (2) | 2 | INCLUDE |
| Xu, 2013 | 269 | ERCC1 polymorphisms | NSCLC (adv) | platinum-based CTX | S (2) | 2,4 | INCLUDE |
| Xu, 2014 | 199 | STAT3, p-STAT3 | NSCLC |  | S (2) | 2 | INCLUDE |
| Xu, 2015 | 5614 | EGFR, WT tumour, smoking status | NSCLC (adv) | EGFR TKIs (erlotinib plus CTX) | M | 2,4 | INCLUDE |
| Xu, 2015 | 5695 | RUNX3 | NSCLC |  | S | 2 | INCLUDE |
| Xue, 2014 | 5037 | CDH13/CDH13 microRNA | NSCLC |  | S (2) | 2 | INCLUDE |
| Yan, 2014 | 192 | NSE | NSCLC |  | S | 2 | INCLUDE |
| Yan, 2014 | 5962 | E-cadherin | NSCLC |  | S | 2 | INCLUDE |
| Yan, 2014 | 6146 | LDH | NSCLC |  | S | 2 | Non-English Language |
| Yang, 2007 | 850 | SOGL | mxdC LC |  | S | 3 |  |
| Yang, 2013 | 318 | BRCA1 | NSCLC | platinum-/toxal- based CTX | S | 2,4 | INCLUDE |
| Yang, 2013 | 338 | microRNA-21, microRNA-155 | NSCLC |  | S (2) | 2 | INCLUDE |
| Yang, 2014 | 19 | E-cadherin | NSCLC |  | S | 2 | INCLUDE |
| Yang, 2014 | 68 | ERCC1 | SCLC | platinum-based CTX | S | 2,4 | INCLUDE |
| Yang, 2014 | 165 | TUBB3 | NSCLC | taxane/vinorebine-based CTX | S | 2,4 | INCLUDE |
| Yang, 2014 | 169 | p-Akt | NSCLC |  | S | 2 | INCLUDE |
| Yang, 2014 | 184 | ERCC1/2 polymorphism | NSCLC | platinum-based CTX | S (2) | 2,4 | INCLUDE |
| Yang, 2014 | 6065 | FGFR1 | NSCLC |  | S | 2 | INCLUDE |
| Yang, 2014 | 6094 | EGFR mut (Del19/L858R) | NSCLC (mut adv) | EGFR TKIs (erlotinib) | S (2) | 4 |  |
| Yang, 2015 | 5761 | smoking status, gen, WT tumour, histology | NSCLC (adv) | EGFR TKIs (erlotinib) | M | 4 |  |
| Yin, 2011 | 526 | ERCC1/2 polymorphism | NSCLC | platinum-based CTX | S (2) | 2,4 | Theme 2 only; INCLUDE |
| Yin, 2015 | 5437 | MicroRNA-200 | mxdC LC |  | S | 2 | Conference abstract |
| Ying, 2015 | 57 | KRAS, WT tumour | NSCLC (adv) | EGFR TKIs | S (2) | 2,4 | INCLUDE |
| Yu, 2004 | 436 | ERCC1 polymorphisms | NSCLC (adv) | platinum-based CTX | S (2) | 2,4 | Treatment effectiveness only |
| Yu, 2015 | 5489 | GTV | NSCLC |  | S | 2 | INCLUDE |
| Zang, 2015 | 5723 | CDKN3, histology (ADC SQCC) | LC |  | S (2) | 2 | Did not meet review criteria |
| Zeng, 2015 | 5601 | IL-17 | NSCLC |  | S | 2 | Theme 1,2 |
| Zeng, 2015 | 5640 | RRM1, ethnicity (Asian) | NSCLC (adv) | CTX (gemcitabine) | S (2) | 2,4 | Theme 2 only; INLCLUDE |
| Zhan, 2009 | 724 | VEGF | LC |  | S | 2 | INCLUDE |
| Zhan, 2013 | 5067 | COX-2 | NSCLC |  | S | 2 | INCLUDE |
| Zhang, 2010 | 7323 | ERCC1 | NSCL (adv) | platinum-based CXT | S | 2,4 | Conference abstract |
| Zhang, 2012 | 420 | TUBB3, ethnicity (Asian) | NSCLC | CTX (paclitaxel/vinorebine) | S (2) | 2,4 | INCLUDE |
| Zhang, 2012 | 442 | cyclin D1 | NSCLC |  | S | 2 | INCLUDE |
| Zhang, 2012 | 446 | survivin | NSCLC |  | S | 2 | INCLUDE |
| Zhang, 2013 | 223 | XRCC3 polymorphism | LC | platinum-based CXT | S | 2,4 | LC only |
| Zhang, 2013 | 346 | Gender | NSCLC (adv) | CRX (Pemetrexed) | S | 4 |  |
| Zhang, 2014 | 11 | EGFR | NSCLC (surR) |  | S | 2 | INCLUDE |
| Zhang, 2014 | 47 | EGFR | NSCLC | EGFR-TKIs | S | 2,4 | INCLUDE |
| Zhang, 2014 | 56 | histology | NSCLC (adv) | Antiangiogenic agents + CTX | S | 2,4 | Theme 4 only |
| Zhang, 2014 | 81 | CTCs | SCLC |  | S | 2 | INCLUDE |
| Zhang, 2014 | 102 | EGRF (del19/ deletion and exon 21 L858R mutation) | NSCLC (adv mut) | EGFR-TKIs | S (2) | 2,4 | Theme 4 only |
| Zhang, 2014 | 113 | EGFR | NSCLC (adv) | CTX | S | 2,4 | INCLUDE |
| Zhang, 2014 | 5035 | IL-17 | mxdC NSCLC |  | S | 2 | INCLUDE |
| Zhang, 2015 | 16 | Smoking status | NSCLC (mut) | EGFR-TKIs | S | 2,4 | Theme 4 only |
| Zhang, 2015 | 121 | Surgical treatment | NSCLC (stage I) | Lobectomy, segmentectomy, wedge resection | S | 4 |  |
| Zhang, 2015 | 4983 | Plt | LC |  | S | 2 | LC only |
| Zhang, 2015 | 5463 | Ethnicity (Asian) | NSCLC | CTX (vinorelbine + cisplatin) | S | 2,4 | Conference abstract |
| Zhang, 2015 | 5572 | DAPK | NSCLC |  | S | 2 | INCLUDE |
| Zhang, 2015 | 5693 | CXCRCR4 | NSCLC |  | S | 2 | INCLUDE |
| Zhang, 2015 | 5707 | LncRNA MALAT | mxdC NSCLC |  | S | 2 | INCLUDE |
| Zhang, 2015 | 5858 | KRAS, WT tumour, testing approach, ethnicity (Asian) | NSCLC (adv mut) | EGFR target therapy | M | 2,4 | Theme 4 only |
| Zhao, 2013 | 243 | NSE | SCLC |  | S | 2 | INCLUDE |
| Zhao, 2013 | 6684 | PTK6 | NSCLC |  | S | 2 | MA only |
| Zhao, 2014 | 69 | IGF1R | NSCLC |  | S | 2 | INCLUDE |
| Zhao, 2014 | 74 | Bcl-2 | NSCLC |  | S | 2 | INCLUDE |
| Zhao, 2014 | 98 | CD44v6 | NSCLC |  | S | 2 | INCLUDE |
| Zhao, 2015 | 5531 | NLR | LC |  | S | 2,4 | Theme 2 only; INCLUDE |
| Zhao, 2015 | 5809 | CXCR4 | mxdC LC |  | S | 2 | LC only |
| Zheng, 2004 | 595 | lymph node micrometastasis (lymphatic invasion status) | NSCLC (I) |  | S | 2 | Non-English Language |
| Zhong, 2015 | 5664 | histology | NSCLC (adv) | CXT (Pemetrexed) | S | 4 |  |
| Zhou, 2013 | 224 | plasma D-dimer | LC |  | S | 2 | LC only |
| Zhou, 2014 | 33 | PLR | mxdC NSCLC |  | S | 2 | INCLUDE |
| Zhou, 2015 | 5673 | histology, PS, EGFR | NSCLC (adv) | Maintenance CTX | M | 4 |  |
| Zhou, 2015 | 5779 | CXCR4 | NSCLC |  | S | 2 | INCLUDE |
| Zhu, 2009 | 7386 | Smoking history, gen, ethnicity (Asian), histology | NSCLC (adv) | EGFR TKIs (erlotinib) | M | 2,4 | Non-English Language |
| Zhu, 2014 | 120 | MicroRNA-21 | mxdC NSCLC |  | S | 2 | INCLUDE |
| Zhu, 2015 | 4969 | TIMP-2 | NSCLC |  | S | 2 | INCLUDE |
| Zhu, 2015 | 5610 | MALAT-1 | mxdC NSCLC |  | S | 2 | INCLUDE |
| Zhuang, 2011 | 489 | p27 | NSCLC |  | S | 2 | INCLUDE |
| Zikos, 2014 | 5031 | Health related quality of life | SCLC |  | S | 2 | Theme 4 |
| Zou, 2015 | 5829 | OPN | NSCLC |  | S | 2 | INCLUDE |

Abbreviations: ADC adenocarcinoma; Adv advanced; AIS adenocarcinoma in situ; ALDH1 aldehyde dehydrogenase 1; Alkphos alkaline phosphatase; ANXA3 annexin A3; Bcl-2 apoptosis regulator B-Cell lymphoma 2; BIM polymorphism Bcl-2-like protein 11 (AKA BIM) Includes BIM gene (deletion) polymorphism; Bmi-1 B-cell-specific moloney leukemia virus insertion site 1; BMM bone marrow micormetastases; BRCA BRCa homolog (tumour suppressor gene BRCA); CADM1 cell adhesion molecule 1; Cav-1 caveolin-1 scaffolding protein tumour suppressor gene; CD133 AKA prominin-1. Glycoprotein encoded by PROM1 gene; CD44 Includes CD44 isoforms, CD44s: standard form; CD44v6: CD44 variant 6cell surface glycoprotein; CDH13 cadherin 13; CDKN cyclin-dependent kinase inhibitor; CEA carcinoembryonic antigen; CIF circulating inflammatory cells; COX-2 cyclooxygenase-2; CRP C-reactive protein; CTCs circulating tumour cells; CTX chemotherapy; CXCR4 C-X-C chemokine receptor 4; CYFRA 21-1 a cytokeratin 19 fragment; DAPK death-associated protein kinase; DFI Disease free interval; E-cadherin epithelial cadherin; EGFR epidermal growth factor receptor; EML4/ALK echinoderm microtubule associated protein like 4 / anaplastic lymphoma receptor tyrosine kinase; EphA2 EPH receptor A2 gene belongs to the ephrin receptor subfamily of the protein-tyrosine kinase family; ERbeta oestrogen receptor beta; ERCC1 excision repair cross-complementation; ESR1 estrogen receptor 1; ESR2 estrogen receptor 2; EZH2 Enhancer of zeste homolog 2; FGFR1 fibroblast growth factor receptor1; FOXM1 forkhead box M1; Gen gender; GGO ground glass-opacity; GTV gross tumour volume; Hb haemoglobin level; HER-2 human epidermal growth factor receptor 2; HIF-1alpha hypoxia inducible factor 1 alpha subunit; IGF1R insulin-like growth factor receptor 1; IL-17 interleukin-17; ILD intestinal lung disease; ITC isolated tumour cells; Ki67 antigen KI-67 protein cellular marker for proliferation; K-RAS Kirsten rat sarcoma viral oncogene homolog; LC lung cancer; LDH serum lactate dehydrogenase; let-7 Let 7 micro rna precursor; LNR lymph node ratio; LRF locoregional failure; LVD lymphatic vascular density; LVI lymphovascular invasion, lymphatic vessel invasion; MALAT1 metastasis associated lung adenocarcinoma transcript 1; MDM2 murine double minute 2 gene; MDR multidrug resistance; MDT multidisciplinary team; MET mesenchymal-epithelial transition; MIA melanoma inhibitory activity; MM micro metastases; MMP matrix metalloproteinase; MPLC multiple primary lung cancers; MRP1 multidrug resistance-associated protein; MT1-MMP membrane-type matrix metalloproteinase; mTOR mammalian target of rapamycin; MVD microvessel density; MVP major vault protein; N Nodal; NF-kappaB nuclear factor-kappaB; NLR neutrophil to Lymphocyte Ratio; NSCLC non small cell lung cancer; NSE neuron-specific enolase; OPN (PSTO, TTO) Osteopontin (OPN) expression. Also includes plasma / serum / pleural effusion osteopontin concentration (PSPO) and tumor tissue osteopontin expression (TTO); P16 p16 promoter hypermethylation / p16 methylation / epigenetic silencing of p16 gene; p-Akt phosphor-Akt; PD-L1 gene expression of programmed cell death 1 (PD-1) and one of its ligands, PD-L1; PIK3CA phosphatidylinositol-4,5-bisphosphate 3-kinase catalytic subunit alpha; PLC pleural lavage cytology; PLR platelet Lymphocyte Ratio. Inflammation parameters; Plt platelet count; p-mTOR phosphorylated mammalian target of rapamycin; PS performance status; PTK6 Expression of protein tyrosine kinase 6; R0 negative resection margins; RAS resistance to audiogenic seizures; RASSF1A methylation Ras association (RalGDS/AF-6) domain family member 1; RRM1 ribonucleotide reductase catalytic subunit M1; S100A4 S100 calcium binding protein A4; SES socioeconomic status; SOGL Similarities of Ordered Gene Lists; SOX-2 SRY (sex determining region Y)-box 2; STAT3 signal transducer and activator of transcription 3; SUV standardized uptake value; SUVMax (FDG-PET) maximum standardized uptake value; MTV, TGL - Metabolic tumour volume, total lesion glycolysis. Volumetric parameters from F-FDG PET; Sync mets Synchronous metastases; Sync T Synchronous tumours; T Tumour; T loc tumour location; T size tumour size; TB tuberculosis; TIMP-2 tissue inhibitor of metalloproteinase-2; TKIs tyrosine kinase inhibitors; TP-53 tumour protein p53; TR tumour response; TS thymidylate synthase; TTF-1 thyroid transcription factor 1; TTP time to tumour progression; TUBB3 Beta tubulin III; VDAC1 voltage dependent anion channel type 1; VDAC2 voltage dependent anion channel type 2; VDR vitamin D receptor; VEGF vascular endothelial growth factor; VitD level Serum vitamin D level; VPI visceral pleural invasion; WBC white blood cell count; WT tumour Wild type tumour; XPD xeroderma pigmentosum group D; XRCC1 x-ray repair cross-complementing group 1
