# Supplementary material for: Pragmatic methods for reviewing exceptionally large bodies of evidence: systematic mapping review and overview of systematic reviews using lung cancer survival as an exemplar
Source: Syst Rev. 2019 Jul 16;8:171. doi: 10.1186/s13643-019-1087-4 (PMC6631880; doi:10.1186/s13643-019-1087-4)
Supplement: Supplementary file 3 — Appendix C. Description of included reviews in stage 2, overview of reviews. Table C1. Summary of included reviews. Table C2. Reference details of included studies. (DOCX 93 kb) [file 13643_2019_1087_MOESM3_ESM.docx]

**APPENDIX C: DESCRIPTION OF INCLUDED REVIEWS IN STAGE 2, OVERVIEW OF REVIEWS**

Criteria used to assess the quality of the included reviews:

ES: explicit search criteria reported;

DB: more than one reference database searched;

EB: used pre-defined eligibility criteria for studies;

RM: used explicit and reproducible methodology;

CH: a systematic presentation of the characteristics of included studies presented;

QA: incorporated an assessment for the validity of the findings of the included studies.

**Table C1: Summary of included reviews**

| **Author, year** | **REV ID** | **Country** | **Search year+** | **ES** | **DB** | **EB** | **RM** | **CH** | **QA** | **PR Theme** | **No PFs** | **LC Type** | **LC subtype**  **(review inclusion)*** | **Histological subtypes of included studies*** | **Treatment** | **Included histology subgroup analysis** | **Synthesis** | **No. Studies** | **Sample Size range** | **Included MVA / UVA** |
| --- | --- | --- | --- | --- | --- | --- | --- | --- | --- | --- | --- | --- | --- | --- | --- | --- | --- | --- | --- | --- |
| Aboshi, 2014 | 182 | Japan | 2012 | Y | N | Y | N | Y | N | 2, 4 | 13 | NSCLC | late | SCC | CTX | Y | S-MR | 65 | NS | NS |
| Ashworth, 2013 | 237 | UK | 2012 | Y | Y | Y | Y | P | N | 1, 2 | Any | NSCLC | met | ADC/SCC/other |  | N | S-N | 49 (23 in analysis of PFs) | NS (total 1793) | Both |
| Ashworth, 2014 | 105 | UK | 2012 | Y | Y | Y | Y | Y | N | 1, 2, 3 | 20 | NSCLC | met | ADC/SCC/other |  | Y | S-MR | 20 | 6-262 | Both |
| Behera, 2016 | 5815 | USA | 2015 | Y | Y | Y | N | Y | N | 2 | 2 | NSCLC | I | ADC | Surgery | Y | S-MA | 19 (11 OS) | 8-110 | NS |
| Berghmans, 2006 | 886 | France | 2005 | Y | N | Y | P | Y | Y | 2 | 1 | NSCLC |  | ADC |  | Y | S-MA | 10 | 50-284 | NS |
| Berghmans, 2008 | 805 | Belgium | 2006 | Y | Y | Y | Y | Y | Y | 2 | 1 | LC | <IV | NSCLC | Any | N | S-MA | 13 | 38-315 | NS |
| Berghmans, 2011 | 8434 | Belgium | 2009 | N | N | Y | N | P | N | 2 | Any | NSCLC | III | NSCLC |  | N | S-N | 39 | 42-2048 | MVA |
| Breen, 2008 | 791 | France | 2007 | Y | N | Y | P | P | N | 2, 4 | 1 | NSCLC | early | NSCLC | Surgery | N | S-N | 9 (7 OS) | 44-760 | NS |
| Brundage, 2002 | 1051 | Canada | 2001 | Y | N | P | N | N | N | 2 | Any | NSCLC |  | NSCLC |  | N | S-N | 887 | 31-1281 | MVA |
| Buttigliero, 2011 | 494 | Italy | 2007 | Y | Y | Y | Y | Y | Y | 2 | 2 | mxdC LC |  | NSCLC |  | N | S-N | 25 (2 LC) | 294-477 | MVA |
| Carlson 2009 | 720 | USA | 2007 | Y | Y | Y | Y | Y | Y | 2 | 3 | NSCLC | WT | NSCLC | EGFR TKIs (gefitinib and erlotinib) | Y | S-MA | 38 | NS | NS |
| Carter, 2014 | 5362 | US | 2010 | Y | Y | Y | P | N | Y | 2, 4 | 12 | NSCLC | III-IV | ADC/other |  | Y | S-N | 54 | NS | MVA |
| Chen 2015 | 5751 | China | 2014 | Y | Y | Y | Y | Y | Y | 2 | 1 | mxdC NSCLC |  | NSCLC |  | Y | S-MA | 3 | 580 | Both |
| Chen, 2010 | 635 | China | 2009 | Y | Y | Y | U | Y | Y | 2, 4 | 1 | NSCLC | late | NSCLC | platinum-based CTX | N | S-MA | 12 | 45 - 200 | NS |
| Chen, 2013 | 257 | China | 2013 | Y | N | Y | P | Y | N | 2 | 1 | NSCLC |  | NSCLC |  | N | S-MA | 8 | 44-758 | NS |
| Chen, 2013 | 6663 | China | 2012 | Y | Y | Y | Y | P | Y | 2,4 | 2 | LC |  | NSCLC/SCLC |  | N | S-MA | 15 (4 OS) | 44-4245 | NS |
| Chen, 2014 | 86 | China | 2013 (IS) | Y | Y | Y | Y | Y | N | 2 | 1 | NSCLC |  | ADC/SCC |  | Y | S-MA | 13 | 43-160 | Both |
| Choma, 2001 | 1085 | France | 1999 | Y | N | Y | P | Y | N | 2 | 1 | NSCLC | surR | ADC/SCC/other | Surgery | N | S-MA | 35 | 44-340 | NS |
| Christopoulos, 2013 | 5789 | Greece | 2013 | Y | Y | Y | Y | N | N | 2 | 1 | LC |  | None | CTX | N | S-N | 17 | 4-56 (NS for 3 studies) | NS |
| Dai, 2015 | 5508 | China | 2015 | Y | N | Y | Y | Y | Y | 2 | 1 | mxdC NSCLC |  | NSCLC |  | N | S-MA | 7 (LC) | 68-201 | NS |
| De Geus-Oui, 2007 | 817 | Netherlands | 2006 | Y | N | Y | N | Y | N | 2, 4 | 1 | NSCLC |  | ADC/SCC/other |  | N | S-N | 17 | 38-315 | NS |
| Deghaidy 2005 | 923 | Egypt | 2004 (IS) | Y | N | Y | Y | N | N | 2 | 5 | LC |  | NSCLC/ ADC/SCC | Surgery | N | S-MA | 13 | 142-16315 | NS |
| Deng, 2015 | 5645 | China | 2011 (IS) | Y | Y | Y | Y | Y | N | 2 | 1 | NSCLC | N0 | ADC/SCC |  | N | S-MA | 10 | 39-821 | MVA |
| Dimou, 2014 | 88 | US | 2014 | Y | N | Y | Y | Y | Y | 2 | 1 | NSCLC |  | ADC | Surgery | Y | S-MA | 9 | 23-906 | NS |
| Ellis, 2010 | 7352 | Canada | 2010 | Y | N | Y | Y | Y | N | 2, 4 | 10 | NSCLC |  | ADC |  | N | S-N | 89 | 79-500 | NS |
| Fan, 2008 | 777 | China | 2007 | Y | N | Y | Y | P | Y | 2 | 1 | NSCLC | SurR | NSCLC/SSC | Surgery | N | S-MA | 10 | 28-219 | NS |
| Florou, 2014 | 51 | Greece | 2013 | Y | Y | Y | Y | Y | N | 2,4 | 1 | mxdC NSCLC/SCLC | Early/limited | NSCLC/SCLC | Surgery | N | S-N | 20 (13 LC; 4 survival only) | 66-2258 | NS |
| Guo, 2014 | 134 | China | 2013 | Y | Y | Y | Y | Y | Y | 2 | 2 | NSCLC |  | ADC/SCC |  | Y | S-MA | 18 | 61-883 | Both |
| Gupta, 2010 | 610 | US | 2010 | Y | N | Y | N | P | N | 2 | 1 | mxdC LC |  | NSCLC/SCLC |  | N | S-N | 59 (10 LC) | 101-150 | MVA |
| He, 2013 | 6625 | China | 2013 | Y | Y | Y | U | Y | Y | 2 | 1 | mxdC LC |  | NSCLC/SCLC |  | N | S-MA | 28 | 30-637 | NS |
| Hirsch, 2008 | 7515 | USA | 2007 | Y | Y | P | P | Y | N | 2,4 | 1 | NSCLC | III-IV | ADC/SCC/LCC/other |  | Y | S-N | 10 (8 OS) | 50-616 | Both |
| Hu, 2013 | 286 | China | 2012 | Y | Y | Y | P | Y | N | 2 | 1 | LC |  | NSCLC/ ADC/SCC |  | N | S-MA | 19 | 41-451 | NS |
| Huang, 2013 | 234 | China | 2013 | Y | Y | Y | Y | Y | N | 2 | 1 | NSCLC |  | ADC/SCC |  | Y | S-MA | 20 | 24-208 | NS |
| Huang, 2013 | 343 | China | 2011 | Y | Y | Y | P | Y | N | 2 | 1 | NSCLC |  | ADC/SCC |  | Y | S-MA | 29 | 43-219 | NS |
| Huang, 2015 | 95 | China | 2014 | Y | Y | Y | Y | Y | Y | 2 | 1 | NSCLC | I-II | NSCLC/ADC | Surgery | N | S-MA | 22 | 73-6048 | NS |
| Huang, 2102 | 454 | China | 2011 | Y | Y | Y | P | Y | N | 2 | 1 | LC |  | NSCLC/ ADC/SCC |  | Y | S-MA | 14 | 62-778 | NS |
| Hubner, 2011 | 483 | UK | 2009 | Y | N | Y | N | Y | N | 2, 4 | 1 | LC |  | NSCLC/SCLC | +/- CTX | Y | S-MA | 23 | 15 - 389 | Both |
| Huncharek, 1999 | 1133 | US | 1997 | N | Y | Y | Y | Y | N | 2 | 1 | NSCLC |  | NSCLC/ADC |  | N | S-MA | 8 | 44-192 | UVA |
| Huncharek, 2000 | 1121 | US | 1997 | N | Y | Y | P | Y | N | 2 | 1 | NSCLC |  | NSCLC |  | N | S-MA | 8 | 54-250 | Both |
| Huo, 2015 | 5784 | China | 2014 | Y | Y | Y | P | Y | N | 2 | 1 | LC |  | NSCLC |  | N | S-MA | 10 | 50-296 | UVA |
| Im, 2015 | 14 | Korea | 2013 | Y | Y | Y | Y | Y | Y | 2 | 2 | NSCLC |  | NSCLC |  | N | S-MA | 13 | 39-529 | UVA |
| Jiang, 2012 | 424 | China | 2011 | Y | Y | Y | Y | Y | N | 2 | 1 | NSCLC |  | ADC |  | N | S-MA | 28 | 11-389 | NS |
| Jiang, 2013 | 312 | China | 2012 | Y | Y | Y | Y | Y | N | 2 | 1 | NSCLC |  | NSCLC/ ADC/SCC |  | Y | S-MA | 19 | 43-259 | UVA |
| Jiang, 2014 | 140 | China | 2014 | Y | Y | Y | Y | Y | N | 2 | 3 | NSCLC |  | NSCLC/ ADC/SCC |  | N | S-MA | 10 | 29-190 | NS |
| Jiang, 2014 | 144 | China | 2013 | Y | Y | Y | Y | Y | N | 2 | 1 | NSCLC |  | P-ADC |  | Y | S-MA | 16 | 49-335 | NS |
| Jiang, 2015 | 5 | China | 2014 | Y | Y | Y | P | P | Y | 2 | 1 | MPLC |  |  | Surgery | Y | S-MA | 22 | 26-234 | NS |
| Jiang, 2015 | 4951 | China | 2014 (IS) | Y | Y | Y | Y | Y | N | 2 | 1 | NSCLC | N0M0 | ADC/SCC/other | Surgery | Y | S-MA | 13 | 142-16315 | NS |
| Jiang, 2015 | 4988 | China | 2014 | Y | Y | Y | Y | Y | N | 2 | 1 | SCC |  | SCC |  | N | S-MA | 13 | 59-628 | NS |
| Jin, 2014 | 67 | China | 2014 | Y | Y | Y | Y | Y | Y | 2 | 1 | NSCLC |  | NSCLC | Surgery | Y | S-MA | 8 | NS | NS |
| Jing, 2015 | 5605 | China | 2014 | Y | Y | Y | N | Y | N | 2 | 1 | NSCLC | SurR | NSCLC | Surgery | N | S-MA | 8 | 79-301 | NS |
| Kilvaer, 2015 | 5533 | Norway | 2014 | Y | N | Y | Y | Y | Y | 2 | 4 | NSCLC |  | ADC |  | Y | S-MA | 30 | 48-335 | UVA |
| Knez, 2011 | 525 | Slovenia | 2010 | Y | N | Y | P | N | N | 2, 4 | 3 | SCLC |  | SCLC | CTX | N | S-N | 20 | 9-248 | Both |
| Kong, 2014 | 157 | China | 2013 | Y | Y | Y | Y | Y | Y | 2 | 1 | NSCLC |  | NSCLC | Surgery | N | S-MA | 7 | 306-1787 | NS |
| Li, 2013 | 272 | China | 2012 | Y | N | Y | N | Y | Y | 2 | 2 | NSCLC |  | NSCLC | Surgery | N | S-MA | 8 | 51-178 | Both |
| Li, 2015 | 4976 | China | 2014 | Y | Y | Y | Y | Y | Y | 2 | 2 | NSCLC |  | ADC/SSC/other |  | Y | S-MA | 10 | 59-276 | Both |
| Li, 2015 | 5436 | China | 2015 | Y | Y | Y | P | Y | Y | 2 | 2 | NSCLC |  | ADC/SSC/other |  | N | S-MA | 10 | 31-178 | Both |
| Liang, 2014 | 183 | China | 2013 | Y | Y | Y | Y | Y | N | 2 | 1 | NSCLC | I-IV | ADC/SCC /LC/other | Surgery in 6 studies | N | S-MA | 7 | 66-452 | Both |
| Liang, 2015 | 5708 | China | 2014 | Y | Y | Y | Y | Y | N | 2 | 1 | LC |  | NSCLC/ SCLC ADC/SCC/other |  | Y | S-MA | 24 (10 LC: 8 OS) | 16-208 | NS |
| Lim, 2010 | 662 | UK | 2008 | Y | Y | Y | Y | Y | N | 2 | 1 | NSCLC |  | None | Surgery | N | S-MR | 11 centres/groups | 36-2950 | MVA |
| Liu 2014 | 76 | China | 2013 (IS) | Y | Y | Y | Y | Y | Y | 2 | 3 | NSCLC |  | NSCLC | Surgery | N | S-MA | 32 | 36-334 | Both |
| Liu, 2010 | 612 | China | 2010 | Y | Y | Y | Y | Y | Y | 2 | 1 | LC |  | NSCLC/SCLC |  | Y | S-MA | 40 | 42-515 | Both |
| Liu, 2013 | 239 | China | 2013 | Y | Y | Y | U | Y | Y | 2, 4 | 1 | NSCLC | I-IV | NSCLC | CTX | N | S-MA | 8 | 28-285 | Both |
| Liu, 2013 | 332 | China | 2012 | Y | Y | Y | Y | Y | N | 2, 4 | 1 | NSCLC |  | NSCLC | EGFR TKIs (gefitinib and erlotinib) | N | S-MA | 33 | 23-2983 | NS |
| Liu, 2015 | 36 | China | 2014 | Y | Y | Y | Y | Y | Y | 2,4 | 1 | NSCLC | IIIB-IV | NSCLC/SCLC |  | Y | S-MA | 24 | 13-193 | UVA |
| Liu, 2015 | 5525 | China | 2015 | Y | Y | Y | Y | Y | Y | 2 | 1 | NSCLC |  | NSCLC |  | N | S-MA | 10 | 55 - 244 | NS |
| Liu, 2015 | 5677 | China | 2014 | Y | Y | Y | Y | P | Y | 2 | 1 | NSCLC | I-II; III; IV | None |  | N | S-MA | 10 | 45-323 | Both |
| Liu, 2016 | 5680 | China | 2015 | Y | P | Y | Y | Y | Y | 2 | 3 | NSCLC | surR | ADC/other |  | Y | S-MA | 36 | 49 - 530 | Both |
| Lou-Quian, 2013 | 334 | China | 2012 | Y | Y | Y | P | Y | N | 2 | 1 | NSCLC |  | ADC |  | Y | S-MA | 25 | 43 - 335 | NS |
| Luan, 2014 | 171 | China | 2013 | Y | Y | Y | Y | Y | Y | 2 | 1 | LC | I-II | ADC/SCC/ LC/other | Surgery | N | S-MA | 18 | 30-731 | Both |
| Luo, 2014 | 107 | China | 2011 | Y | P | Y | Y | Y | N | 2 | 2 | NSCLC |  | NSCLC |  | N | S-MA | 38 | 20-231 | Both |
| Luo, 2015 | 58 | China | 2014 | Y | Y | Y | N | Y | Y | 2 | 3 | NSCLC | <IV | NSCLC | Surgery | N | S-MA | 12 | 30-1402 | NS |
| Luo, 2015 | 5651 | China | 2014 | Y | P | Y | Y | Y | N | 2 | 1 | NSCLC |  | NSCLC |  | N | S-MA | 14 | 58 - 301 | Both |
| Ma, 2012 | 395 | China | 2012 | Y | N | Y | Y | Y | N | 2 | 1 | NSCLC |  | ADC/SCC/other |  | N | S-MA | 8 | 30-631 | Both |
| Ma, 2012 | 401 | China | 2012 | Y | N | Y | Y | Y | N | 2 | 1 | LC |  | NSCLC/SCLC/ ADC/SCC/other |  | Y | S-MA | 16 | 28-208 | Both |
| Ma, 2014 | 190 | China | 2013 | Y | Y | Y | Y | Y | N | 2 | 1 | LC |  | ADC |  | Y | S-MA | 11 | 56-343 | Both |
| Ma, 2015 | 5676 | China | 2014 | Y | Y | Y | Y | Y | N | 2 | 1 | NSCLC |  | None | EGFR-TKIs (gefitinib and erlotinib) | N | S-MA | 6 | 70-239 | NS |
| Marchevsky, 2010 | 625 | USA | 2008 | Y | P | Y | N | Y | N | 2 | 1 | NSCLC | I-II | ADC/SCC/other | Surgery | N | S-MA | 14 | NS | NS |
| Martin, 2003 | 1024 | France | 2002 | Y | N | Y | P | P | Y | 2 | 1 | LC |  | NSCLC/SCLC/ other |  | Y | S-MA | 28 | 23-485 | UVA |
| Martin, 2004 | 969 | France | 2002 | Y | N | Y | P | N | Y | 2 | 1 | LC |  | NSCLC |  | Y | S-MA | 37 | NS | UVA |
| Mascaux, 2005 | 961 | Belgium | 2003 | Y | Y | Y | Y | Y | Y | 2 | 1 | LC |  | NSCLC/ ADC/ SCC |  | Y | S-MA | 43 | 21-355 | NS |
| Mascaux, 2006 | 899 | Belgium | 2005 | Y | N | Y | P | Y | Y | 2 | 1 | LC |  | NSCLC |  | Y | S-MA | 14 | 53-259 | NS |
| Meert, 2002 | 1045 | Belgium | 2001 | Y | Y | Y | Y | Y | Y | 2 | 1 | LC |  | NSCLC |  | Y | S-MA | 16 | 19-505 | NS |
| Meert, 2002 | 1050 | Belgium | 2001 | Y | N | Y | P | Y | Y | 2 | 1 | LC | (surR) | NSCLC | Surgery | N | S-MA | 32 | 15-515 | NS |
| Meert, 2003 | 1019 | Belgium | 2002 | Y | N | Y | P | Y | Y | 2 | 1 | LC |  | NSCLC |  | N | S-MA | 30 | 31-483 | NS |
| Mei, 2013 | 319 | China | 2012 | Y | Y | Y | Y | Y | Y | 2 | 1 | NSCLC |  | ADC |  | Y | S-MA | 12 | 38-522 | Both |
| Meng, 2013 | 287 | China | 2012 | Y | Y | Y | Y | Y | Y | 2 | 1 | NSCLC |  | ADC |  | Y | S-MA | 41 | 41-1118 | NS |
| Miao, 2012 | 6341 | China | 2011 | Y | Y | Y | P | Y | Y | 2 | 1 | ADC | surR | ADC | Surgery | N | S-MA | 13 | 52-359 | Both |
| Mitsudomi, 2000 | 1110 | Japan | 1999 | Y | N | Y | N | Y | N | 2 | 1 | NSCLC | surR | ADC/SCC | Surgery | Y | S-MA | 42 | 39-209 | Both |
| Mollberg, 2014 | 196 | USA | 2012 | Y | P | Y | Y | Y | Y | 2 | 1 | NSCLC | I | NSCLC |  | N | S-MA | 20 | 47 - 1,929 | Both |
| Montazeri, 2009 | 703 | Iran | 2008 | Y | Y | Y | N | Y | N | 2 | 1 | mxdC NSCLC/SCLC |  | NSCLC/SCLC/ LC/other |  | N | S-N | 104 (26 LC) | 30-651 | NS |
| Na, 2014 | 133 | China | 2013 | Y | N | Y | Y | Y | Y | 2 | 1 | NSCLC | <IV | NSCLC | RT | N | S-MA | 13 | 46-132 | Both |
| Nair, 2009 | 706 | USA | 2008 | Y | Y | Y | Y | Y | Y | 2 | 1 | NSCLC | I-II | NSCLC | Surgery | N | S-N | 9 | 36-380 | Both |
| Nakamura, 2005 | 952 | Japan | 2004 | Y | N | Y | Y | Y | N | 2 | 1 | NSCLC |  | ADC/SCC/other | Surgery | N | S-MA | 20 | 42-408 | NS |
| Nakamura, 2006 | 918 | Japan | 2004 | Y | N | Y | U | Y | N | 2 | 1 | NSCLC | IIIB-IV | ADC/SCC/other | Surgery | N | S-MA | 18 | 19-515 | NS |
| Nakamura, 2011 | 481 | Japan | 2009 | Y | N | Y | Y | Y | N | 2 | 1 | NSCLC |  | NSCLC | Surgery | N | S-MA | 39 | 45-19072 | Both |
| Neal, 2015 | 8441 | UK | 2013 | Y | Y | Y | Y | Y | Y | 2 | 15 | mxdC LC |  | NSCLC |  | N | S-N | 209 (20 LC) | 103-566 | NS |
| Olsson 2009 | 722 | USA | 2007 | N | Y | Y | Y | Y | Y | 2 | 1 | LC |  | None |  | N | S-N | 18 | NS | Both |
| Paesmans, 2010 | 667 | Belgium | 2009 | Y | N | Y | Y | Y | Y | 2 | 1 | NSCLC |  | NSCLC/SCLC |  | Y | S-MA | 24 | 19-487 | UVA |
| Pan, 5774 | 5774 | China | 2014 | Y | Y | Y | Y | Y | Y | 2 | 1 | NSCLC |  | None |  | Y | S-MA | 9 | 47-544 | Both |
| Parsons, 2010 | 695 | UK | 2008 | Y | Y | Y | Y | Y | Y | 1, 2 | 1 | LC |  | NSCLC/SCLC |  | N | S-MA | 10 | 61-611 | Both |
| Peng, 2012 | 437 | China | 2011 | Y | Y | Y | Y | Y | N | 2 | 1 | NSCLC |  | ADC/SCC/LCC/ other |  | Y | S-MA | 17 | 37-218 | UVA |
| Peng, 2014 | 5929 | China | 2014 | Y | Y | Y | Y | Y | Y | 2 | 1 | NSCLC |  | ADC/SCC/other |  | N | S-MA | 13 | 53-244 | Both |
| Peng, 2015 | 5752 | China | 2014 | Y | Y | Y | Y | Y | Y | 2,4 | 1 | NSCLC |  | ADC/SCC/other |  | N | S-MA | 12 | 39-388 | Both |
| Petrelli, 2012 | 389 | Italy | 2012 | Y | N | Y | P | Y | N | 2, 4 | 1 | NSCLC |  | NSCLC | EGFR TKIs (gefitinib and erlotinib) | N | S-MA | 24 (7 OS) | 25 - 651 | MVA |
| Prades, 2015 | 5807 | Spain | 2012 | Y | N | Y | Y | Y | N | 2 | 1 | mxdC LC |  | NSCLC |  | N | S-N | 51 | NS | NS |
| Pujol, 2004 | 993 | Germany | 2001 | Y | Y | Y | Y | Y | N | 2 | 1 | NSCLC |  | P-ADC/SCC/other | Surgery | Y | S-MA | NS | NS (total 20063 | MVA |
| Qian, 2010 | 657 | China | 2008 | Y | Y | Y | Y | Y | Y | 2 | 1 | NSCLC | <IV | NSCLC/ADC | Surgery | N | S-MA | 11 | 32-218 | NS |
| Qin, 2013 | 229 | China | 2013 | Y | N | Y | Y | Y | N | 2,4 | 2 | NSCLC | Late | None | Platinum-based CTX | N | S-MA | 24 | 33-632 | NS |
| Qiu, 2013 | 221 | China | 2013 | Y | Y | Y | Y | Y | Y | 2 | 1 | NSCLC |  | NSCLC | Surgery | N | S-MA | 9 | 46-265 | Both |
| Qiu, 2013 | 242 | China | 2013 | Y | Y | Y | Y | Y | N | 2 | 1 | NSCLC |  | NSCLC | platinum-based CTX | N | S-MA | 14 | 62-355 | NS |
| Qiu, 2014 | 5592 | China | 2013 | N | Y | Y | Y | Y | N | 2 | 1 | NSCLC |  | ADC/SCC |  | Y | S-MA | 15 | 50-76 | Both |
| Qu, 2013 | 233 | China | 2013 | Y | Y | Y | Y | Y | N | 2 | 1 | NSCLC | IIIB-IV | NSCLC |  | N | S-MA | 11 | 50-161 | NS |
| Quinton, 2011 | 6781 | Canada | 2011 | Y | P | Y | N | Y | Y | 2, 4 | 5 | NSCLC | IIIB-IV | ADC/SCC |  | N | S-N | 26 | NS | NS |
| Ren, 2013 | 249 | China | 2012 | Y | Y | Y | Y | Y | Y | 2 | 5 | LC |  | SCC/ADC/LCC |  | Y | S-MA | 30 (4 OS) | 29 - 328 | NS |
| Roth, 2011 | 477 | USA | 2010 | Y | Y | Y | Y | Y | Y | 2, 4 | 1 | NSCLC | IIIB-IV | NSCLC | platinum-based CTX | N | S-MA | 11 (8 OS MA) | 40-163 | MVA |
| Salah, 2012 | 467 | Jordan | 2010 (IS) | Y | N | Y | U | Y | N | 2 | 8 | NSCLC | met (isolated) | ADC/SCC/other | Surgery | N | S-MR | 51 | 62 cases | Both |
| Saso, 2012 | 427 | UK | 2011 | Y | Y | Y | Y | P | Y | 2 | 1 | NSCLC | surR | NSCLC |  | N | S-MA | 17 | 65 -2,113 | NS |
| Shao, 2014 | 122 | China | 2014 | Y | Y | Y | Y | Y | Y | 2 | 1 | mxdC NSCLC |  | NSCLC |  | N | S-MA | 16 (3 LC) | 37 - 249 (LC 53 -164) | Both |
| Shao, 2015 | 5618 | China | 2014 | Y | Y | Y | P | Y | U | 2 | 1 | NSCLC |  | NSCLC |  | N | S-MA | 7 | 44 - 758 | Both |
| Shen, 2013 | 266 | China | 2013 | Y | Y | Y | Y | Y | N | 2, 4 | 1 | NSCLC | late | None | platinum-based CTX | N | S-MA | 11 | 62 - 460 | NS |
| Shen, 2014 | 62 | China | 2014 | Y | Y | Y | Y | Y | Y | 2 | 1 | mxdC NSCLC |  | ADC/SCC |  | N | S-MA | 23 (4 NSCLC) | 94-279 | NS |
| Slatore, 2010 | 621 | USA | 2008 | Y | Y | Y | P | Y | Y | 2 | 1 | LC |  | LC/NSCLC |  | N | S-N | 23 | 249 - 693,697 | Both |
| Soo, 2011 | 520 | Singapore | 2010 | Y | N | Y | P | Y | N | 2, 4 | 1 | NSCLC | late | None | CTX | N | S-MA | 191 | NS | NS |
| Steels, 2001 | 5212 | France | 1999 | Y | N | Y | P | P | Y | 2 | 1 | LC |  | NSCLC/ ADC/SCC |  | Y | S-MA | 74 | 14-530 | UVA |
| Sun, 2013 | 6459 | China | 2011 | Y | Y | Y | Y | N | N | 2 | 1 | NSCLC |  | NSCLC |  | N | S-MA | 31 (4 OS) | 43-138 | MVA |
| Sun, 2015 | 5469 | China | 2015 | Y | Y | Y | Y | Y | Y | 2 | 1 | NSCLC |  | NSCLC |  | N | S-MA | 12 | 75 - 11,324 | Both |
| Tanvetyanon, 2008 | 799 | US | 2007 | Y | N | Y | N | P | N | 1, 2 | 1 | NSCLC | met | NSCLC/ADC | Surgery | N | S-MA | 10 | 4-29 | NS |
| Tanvetyanon, 2013 | 320 | US | 2011 | Y | Y | Y | N | Y | Y | 2 | 6 | NSCLC | NSCLC (sync, surR) | NSCLC | Surgery | N | S-MR | 6 | 26-116 | MVA |
| Tanvetyanon, 2015 | 23 | US | 2012 | U | U | Y | N | Y | N | 2, 3 | 1 | NSCLC | sync | ADC/other | Surgery | Y | S-MR | 6 | NS | Both |
| Tian, 2015 | 4978 | China | 2015 | Y | P | Y | P | Y | Y | 2 | 1 | mxdC LC |  | NSCLC |  | N | S-MA | 14 (3 LC) | 19 - 222 | Both |
| Tong, 2011 | 486 | China | 2010 | Y | Y | Y | Y | Y | N | 2 | 1 | NSCLC |  | NSCLC/ ADC/SCC |  | Y | S-MA | 20 | 38 - 219 | Both |
| Trivella, 2007 | 846 | UK | 2001 | Y | NA | Y | N | N | P | 2 | 1 | NSCLC | <IV | NSCLC |  | N | S-MR | 17 | 33-1173 | MVA |
|  |  |  |  |  |  |  |  |  |  |  |  |  |  |  |  |  |  |  |  |  |
| Vansteenkiste, 2004 | 982 | Belgium | 2003 | Y | Y | Y | U | Y | U | 2, 4 | 1 | LC |  | NSCLC |  | N | S-N | 31 | 13 - 153 | Both |
| von Meyenfeldt, 2012 | 414 | Netherlands | 2011 | Y | Y | Y | Y | P | N | 2 | 3 | LC |  |  | Surgery | N | MA | 19 | 987-90088 | MVA |
| Wang 2014 | 180 | China | 2013 | Y | Y | Y | Y | Y | Y | 2, 4 | 1 | LC | surR | NSCLC/SCLC |  | Y | S-MA | 23 | 48-856 | NS |
| Wang 2015 | 5514 | China | 2014 | Y | Y | Y | Y | Y | Y | 2 | 1 | NSCLC |  | ADC/SCC |  | Y | S-MA | 15 | 28-161 | NS |
| Wang, 2011 | 469 | China | 2011 | Y | Y | Y | Y | Y | Y | 2 | 1 | NSCLC | surR | NSCLC |  | N | S-MA | 52 | 35 - 2295 | Both |
| Wang, 2011 | 534 | China | 2010 | Y | Y | Y | Y | Y | Y | 2 | 1 | NSCLC | surR | None |  | N | S-MA | 19 | 50 - 351 | Both |
| Wang, 2012 | 356 | China | 2012 | Y | Y | Y | Y | Y | Y | 2 | 1 | NSCLC | surR | NSCLC |  | Y | S-MA | 53 | 26 - 2295 | Both |
| Wang, 2012 | 448 | China | 2011 | Y | Y | Y | P | Y | Y | 2 | 1 | NSCLC |  | NSCLC/ADC |  | N | S-MA | 12 | 50-237 | NS |
| Wang, 2013 | 247 | China | 2013 | Y | Y | Y | Y | Y | Y | 2 | 1 | NSCLC | IIIA-IV | NSCLC | CTX | N | S-MA | 8 | 24-193 | NS |
| Wang, 2013 | 268 | China | 2012 | Y | Y | Y | Y | Y | P | 2 | 2 | NSCLC |  | None |  | N | S-MA | 16 | 30 -637 | NS |
| Wang, 2014 | 129 | China | 2013 | Y | Y | Y | Y | Y | Y | 2, 4 | 1 | NSCLC | IIIB-IV | NSCLC | EGFR TKIs (gefitinib and erlotinib) | N | S-MA | 22 | 17-57 | NS |
| Wang, 2014 | 150 | China | 2014 | Y | Y | Y | Y | Y | Y | 2 | 1 | NSCLC | <IV | NSCLC | Surgery | Y | S-MA | 16 | 43-1000 | Both |
| Wang, 2014 | 151 | China | 2013 | Y | N | Y | P | Y | N | 2 | 1 | NSCLC |  | NSCLC |  | Y | S-MA | 13 | 29 - 305 | NS |
| Wang, 2014 | 187 | China | 2013 | Y | Y | Y | Y | Y | Y | 2 | 1 | LC | <IV | NSCLC/SCLC | Surgery | Y | S-MA | 13 | 45-178 | NS |
| Wang, 2014 | 191 | China | 2013 | Y | Y | Y | Y | Y | Y | 2, 4 | 1 | NSCLC |  | NSCLC | Pemetrexed-with CTX | N | S-MA | 8 | 24 - 285 | NS |
| Wang, 2015 | 103 | China | 2014 | Y | Y | Y | Y | P | N | 2 | 1 | NSCLC | <IV | NSCLC/ ADC/SCC | Surgery | Y | S-MA | 6 | 109-303 | MVA |
| Wang, 2015 | 5537 | China | 2014 | Y | Y | Y | Y | Y | Y | 2 | 1 | NSCLC | <IV | ADC/SCC | Surgery | N | S-MA | 6 | 37-335 | NS |
| Wei 2015 | 4957 | China | 2014 | Y | Y | Y | Y | Y | N | 2 | 1 | LC |  | NSCLC/ADC |  | Y | S-MA | 11 | 52-282 | NS |
| Wen 2015 | 5684 | China | 2014 | Y | Y | Y | Y | Y | Y | 2 | 1 | NSCLC |  | ADC/SCC |  | Y | S-MA | 32 | 58-4534 | UVA |
| Wu 2007 | 848 | China | 2006 (IS) | Y | Y | Y | Y | Y | Y | 2, 4 | 1 | NSCLC | Early, EGFR | ADC/SCC/other | EGFR TKIs (gefitinib) | N | S-MA | 6 | 52-142 | Both |
| Wu 2012 | 390 | China | 2011 (IS) | Y | N | Y | Y | Y | Y | 2 | 1 | NSCLC |  | NSCLC | Surgery | N | S-MA | 21 | 73-391 | NS |
| Wu 2012 | 400 | China | 2010 | Y | Y | Y | Y | Y | N | 2 | 2 | NSCLC |  | NSCLC | platinum-based CTX | N | S-MA | 13 | 57-200 | NS |
| Wu 2014 | 30 | China | 2014 | Y | Y | Y | Y | Y | N | 2 | 1 | NSCLC |  | ADC/SCC |  | N | S-MA | 23 | 55-305 | Both |
| Wu 2014 | 125 | China | 2014 | Y | Y | Y | Y | Y | N | 2 | 1 | mxdC NSCLC/SCLC |  | NSCLC/SCLC |  | Y | S-MA | 21 | 23-549 | Both |
| Wu 2015 | 5510 | China | 2015 | Y | Y | Y | Y | Y | Y | 2 | 1 | LC |  | NSCLC |  | N | S-MA | 44 | 88-334 | NS |
| Xia 2014 | 78 | China | 2014 | Y | Y | Y | Y | Y | N | 2 | 4 | mxdC LC |  | NSCLC |  | N | S-MA | 19 | NS | NS |
| Xie 2012 | 368 | China | 2011 | Y | Y | Y | N | Y | N | 2 | 2 | NSCLC |  | ADC/SCC/other |  | N | S-MA | 7 | 48-244 | NS |
| Xie, 2016 | 5563 | China | 2015 | Y | Y | Y | Y | Y | N | 2 | 1 | NSCLC |  | SCC |  | Y | S-MA | 18 | 100-628 | MVA |
| Xing 2013 | 5058 | China | 2012 | Y | Y | Y | Y | Y | Y | 2 | 1 | mxdC NSCLC | surR | NSCLC | Surgery | N | S-MA | 11 | 1654 | Both |
| Xu, 2013 | 148 | China | 2013 | Y | Y | Y | Y | Y | Y | 2 | 1 | mxdC LC | <IV | NSCLC/SCLC |  | N | S-MA | 5 | 54-639 | UVA |
| Xu, 2013 | 269 | China | 2013 | Y | Y | Y | Y | Y | Y | 2 | 2 | NSCLC | late | NSCLC | platinum-based CTX | N | S-MA | 39 | 44-3110 | NS |
| Xu, 2014 | 199 | China | 2012 | Y | Y | Y | Y | Y | N | 2 | 1 | NSCLC |  | NSCLC |  | N | S-MA | 17 | 40-222 | NS |
| Xu, 2015 | 5614 | China | 2014 | Y | Y | Y | Y | Y | Y | 2, 4 | 2 | NSCLC | late | NSCLC | CTX | N | S-MA | 9 | 54-1159 | NS |
| Xu, 2015 | 5695 | China | 2014 | Y | Y | Y | Y | Y | N | 2 | 1 | NSCLC |  | NSCLC |  | N | S-MA | 13 | 9-120 | NS |
| Xue, 2014 | 5037 | China | 2014 | Y | Y | Y | Y | Y | Y | 2 | 1 | NSCLC |  | ADC/SCC |  | Y | S-MA | 11 | 28-150 | NS |
| Yan, 2014 | 192 | China | 2013 | Y | Y | Y | Y | Y | N | 2 | 1 | NSCLC |  | NSCLC | CTX | N | S-MA | 8 | NS | NS |
| Yan, 2014 | 5962 | China | 2014 | Y | Y | Y | Y | N | Y | 2 | 1 | NSCLC |  | NSCLC |  | N | S-MA | 14 | NS (total 2395) | NS |
| Yang 2013 | 338 | China | 2012 (IS) | Y | Y | Y | Y | Y | Y | 2 | 1 | NSCLC |  | ADC/SCC/other |  | N | S-MA | 29 | 53-193 | Both |
| Yang, 2013 | 318 | China | 2012 | Y | Y | Y | Y | Y | N | 2, 4 | 1 | NSCLC |  | NSCLC | platinum-based CTX | N | S-MA | 23 | 34-769 | NS |
| Yang, 2014 | 19 | China | 2014 | Y | Y | Y | Y | Y | Y | 2 | 1 | NSCLC |  | NSCLC |  | N | S-MA | 29 | 38-405 | NS |
| Yang, 2014 | 68 | China | 2014 | Y | Y | Y | Y | Y | Y | 2, 4 | 1 | SCLC |  | Limited/extensive stage | platinum-based CTX | Y | S-MA | 9 | 64-323 | NS |
| Yang, 2014 | 165 | China | 2014 | Y | Y | Y | Y | Y | Y | 2, 4 | 1 | NSCLC |  | NSCLC | CTX | N | S-MA | 28 | 19-577 | NS |
| Yang, 2014 | 169 | China | 2013 | Y | Y | Y | Y | Y | Y | 2 | 1 | NSCLC |  | NSCLC | Surgery | N | S-MA | 18 | 34-332 | MVA |
| Yang, 2014 | 184 | China | 2013 | Y | Y | Y | Y | Y | N | 2, 4 | 4 | NSCLC |  | NSCLC | platinum-based CTX | N | S-MA | 46 | 32-640 | NS |
| Yang, 2014 | 6065 | China | 2014 | Y | Y | Y | Y | Y | Y | 2 | 1 | NSCLC |  | SCC |  | Y | S-MA | 6 | 100-264 | NS |
| Yin, 2011 | 526 | US | 2010 | Y | Y | Y | U | Y | U | 2 | 4 | NSCLC |  | NSCLC | platinum-based CTX | N | S-MA | 17 | 33-381 | NS |
| Ying, 2015 | 57 | China | 2014 | Y | Y | Y | Y | Y | U | 2, 4 | 2 | NSCLC |  | NSCLC | EGFR TKIs (gefitinib and erlotinib) | Y | S-MA | 12 | 42-1692 | NS |
| Yu, 2015 | 5489 | China | 2014 | Y | Y | Y | U | Y | Y | 2 | 1 | NSCLC |  | NSCLC | RT | N | S-MA | 10 | 21-173 | NS |
| Zeng, 2015 | 5601 | China | 2013 | Y | Y | Y | Y | Y | N | 1, 2 | 1 | mxdC NSCLC |  | NSCLC |  | N | S-MA | 28 (3LC) | 17-300 (LC: 52-102 | MVA |
| Zeng, 2015 | 5640 | China | 2014 | Y | Y | Y | Y | Y | Y | 2 | 1 | NSCLC | late | NSCLC | CTX | N | S-MA | 12 | 16-95 | NS |
| Zhan, 2009 | 724 | China | 2008 | Y | Y | Y | Y | Y | Y | 2 | 3 | LC |  | NSCLC/ SCLC/ADC |  | Y | S-MA | 51 | 34-335 | NS |
| Zhan, 2013 | 5067 | China | 2013 | Y | Y | Y | Y | Y | N | 2 | 1 | NSCLC |  | ADC |  | Y | S-MA | 16 | 60-253 | MVA |
| Zhang, 2012 | 420 | China | 2011 (IS) | Y | Y | Y | Y | Y | Y | 2, 4 | 1 | NSCLC | IIIB-IV | NSCLC | platinum-based CTX | N | S-MA | 10 | 19-93 | NS |
| Zhang, 2012 | 442 | China | 2011 | Y | Y | Y | Y | Y | Y | 2 | 1 | NSCLC | IIIB-IV | NSCLC/ ADC/SCC |  | Y | S-MA | 24 | 43-390 | NS |
| Zhang, 2012 | 446 | China | 2011 | Y | Y | Y | Y | Y | Y | 2 | 1 | NSCLC | IIIB-IV | NSCLC/ ADC/SCC | Surgery | Y | S-MA | 13 | 43-219 | Both |
| Zhang, 2014 | 11 | China | 2014 | Y | Y | Y | Y | Y | Y | 2 | 1 | NSCLC | <IV | NSCLC /ADC | Surgery | Y | S-MA | 22; 17 in meta-analysis | 53-307 | Both |
| Zhang, 2014 | 47 | China | 2014 | Y | Y | Y | Y | Y | N | 2, 4 | 1 | NSCLC | IIIB-IV | NSCLC | EGFR TKIs (gefitinib and erlotinib) | N | S-MA | 17 | 135-1466 | NS |
| Zhang, 2014 | 81 | China | 2014 | Y | N | Y | N | Y | N | 2 | 1 | SCLC |  | NSCLC |  | N | S-MA | 7 | 30-97 | NS |
| Zhang, 2014 | 113 | China | 2013 | Y | Y | Y | Y | Y | Y | 2, 4 | 1 | NSCLC | IIIB-IV | NSCLC | CTX | N | S-MA | 23 | 54-353 | Both |
| Zhang, 2014 | 5035 | China | 2014 | Y | Y | Y | Y | Y | Y | 2 | 1 | mxdC NSCLC | IIIB-IV | None | Surgery | N | S-MA | 3 | 32-300 | NS |
| Zhang, 2015 | 5572 | China | 2014 | Y | Y | Y | Y | Y | Y | 2 | 1 | NSCLC | <IV | ADC/SCC | Surgery | Y | S-MA | 14 (4 survival) | 28-200 | NS |
| Zhang, 2015 | 5693 | China | 2014 | Y | Y | Y | Y | Y | Y | 2 | 1 | NSCLC |  | NSCLC | Surgery | N | S-MA | 19 | 125-236 | Both |
| Zhang, 2015 | 5707 | China | 2014 | Y | Y | Y | Y | Y | N | 2 | 1 | mxdC NSCLC | <IV | NSCLC | None | N | S-MA | 8 | 50-102 | UVA |
| Zhao 2014 | 98 | China | 2014 (IS) | Y | Y | Y | Y | Y | N | 2 | 1 | NSCLC | met | ADC/SCC |  | Y | S-MA | 10 | 34-190 | NS |
| Zhao, 2013 | 243 | China | 2012 (IS) | Y | Y | Y | Y | P | Y | 2 | 1 | SCLC |  | SCLC | CTX | N | S-MA | 11 | NS | NS |
| Zhao, 2014 | 69 | China | 2014 | Y | Y | Y | Y | Y | Y | 2 | 1 | NSCLC | IIIB-IV | NSCLC | Surgery | N | S-MA | 17 | 39-459 | Both |
| Zhao, 2014 | 74 | China | 2013 | Y | Y | Y | Y | Y | Y | 2 | 1 | NSCLC | IIIB-IV | NSCLC |  | Y | S-MA | 50 | 45-535 | Both |
| Zhao, 2015 | 5531 | China | 2015 | Y | Y | Y | Y | Y | Y | 2 | 1 | LC | Mixed | NSCLC/SCLC |  | Y | S-MA | 22 (21 OS) | 59-1238 | NS |
| Zhou 2015 | 5779 | China | 2014 | Y | Y | Y | Y | Y | Y | 2 | 1 | NSCLC |  | NSCLC |  | N | S-MA | 13 | 34-236 | NS |
| Zhou, 2014 | 33 | China | 2014 | Y | Y | Y | Y | Y | Y | 2 | 1 | mxdC NSCLC |  | NSCLC | CTX | N | S-MA | 26; 3 NSCLC | 94-210 | NS |
| Zhu, 2014 | 120 | China | 2013 | Y | Y | Y | Y | Y | Y | 2 | 1 | mxdC NSCLC | III | NSCLC | Surgery | N | S-MA | 25 (3 NSCLC) | 88-191 | NS |
| Zhu, 2015 | 4969 | China | 2014 | Y | Y | Y | Y | Y | Y | 2 | 1 | NSCLC | IIIB-IV | ADC/SCC |  | N | S-MA | 12; 5 MA | 42-148 | NS |
| Zhu, 2015 | 5610 | China | 2015 | Y | Y | Y | Y | Y | Y | 2 | 1 | mxdC NSCLC |  | NSCLC |  | N | S-MA | 8 (2 NSCLC) | 31-222 | NS |
| Zhuang, 2011 | 489 | China | 2010 | Y | Y | Y | Y | Y | Y | 2 | 1 | NSCLC | <IV | ADC/SCC/other |  | N | S-MA | 11 | 56-405 | NS |
| Zou, 2015 | 5829 | China | 2013 | Y | Y | Y | Y | Y | Y | 2 | 1 | NSCLC | III | NSCLC | Surgery and CTX | N | S-MA | 6 | 67-244 | NS |
| ***Total number of reviews that met the criterion:*** | | | | **201** | **158** | **205** | **150** | **183** | **114** |  | | | | | | | | | | |

+ Where the search dates were not reported this was based on the latest publication year of included studies (IS).

* Histological subtypes of included studies describes the narrow subtypes for which data is available; LC subtype describe the review’s inclusion criteria.

**Berghmans, 2011 [ID 8434]:** MEDLINE was the only database searched (supplemented by scanning the reference lists of relevant studies), and no search terms or dates were provided. However, the review did include a clearly focused question and pre-defined inclusion criteria.

**Tanvetyanon, 2015 [ID 23]:** The literature search and procurement for the individual patient data (IPD) dataset was based on the review by Tanvetyanon, 2013 (review ID 320). The current search was based on Medline only (2000-2012). The authors of publications containing at least 10 studies were contacted for individual-level data. The IPD dataset was described as being updated to January 2014, but included the same number of patients as the 2013 review (n=467). It was not stated if the current dataset was based on the same studies. The number of eligible studies identified, number of IPD datasets obtained, and the number of patients included in each datasets were not stated. The 2013 review identified 10 eligible studies, with IPD available from 6.

**Huncharek, 1999 [ID 1133] and Huncharek, 2000 [ID 1121]:** The literature search included 3 references databases. The search terms were not reported, but the search years covered were given.

**Abbreviations:** ADC adenocarcinoma; CTX chemotherapy; EGFR epidermal growth factor receptor; IS included studies; LC lung cancer; MA meta-analysis; met metastasis; MPLC multiple primary lung cancer; MR meta regression; MVA multivariate analysis; mxdC mixed cancer; NS not stated; N no; No. number; N-S narrative synthesis; NSCLC non small cell lung cancer; OS overall survival; PF prognostic factor; PR Prognostic research; REV ID review unique identification number RT radiotherapy; S synthesis; SCC squamous cell carcinoma; SCLC small cell lung cancer; surR surgical resection; TKIs tyrosine kinase inhibitors; UVA univariate analysis; Y yes

**Table C2: Reference details of included studies**

| **Author, year** | **REV ID** | **Full reference** |
| --- | --- | --- |
| Aboshi, 2014 | 182 | Aboshi M, Kaneko M & Narukawa M (2014): Factors affecting the association between overall survival and progression-free survival in clinical trials of first-line treatment for patients with advanced non-small cell lung cancer. *Journal of Cancer Research & Clinical Oncology* **140**, 839-848. |
| Ashworth, 2013 | 237 | Ashworth A, Rodrigues G, Boldt G & Palma D (2013): Is there an oligometastatic state in non-small cell lung cancer? A systematic review of the literature. *Lung Cancer* **82**, 197-203. |
| Ashworth, 2014 | 105 | Ashworth AB, Senan S, Palma DA, Riquet M, Ahn YC, Ricardi U, Congedo MT, Gomez DR, Wright GM, Melloni G, Milano MT, Sole CV, De Pas TM, Carter DL, Warner AJ & Rodrigues GB (2014): An individual patient data metaanalysis of outcomes and prognostic factors after treatment of oligometastatic non-small-cell lung cancer. *Clinical Lung Cancer* **15**, 346-355. |
| Behera, 2016 | 5815 | Behera M, Owonikoko T, Gal A, Steuer C, Kim S, Pillai RN, Khuri F, Ramalingam S & Sica G (2016): Lung adenocarcinoma staging based on 2011 IASLC/ATS/ERS classification: A pooled analysis of adenocarcinoma in situ (AIS) and minimally invasive adenocarcinoma (MIA). *Clinical Lung Cancer* **17**, e57-64. |
| Berghmans, 2008 | 805 | Berghmans T, Dusart M, Paesmans M, Hossein-Foucher C, Buvat I, Castaigne C, Scherpereel A, Mascaux C, Moreau M, Roelandts M, Alard S, Meert A-P, Patz EF, Jr., Lafitte J-J, Sculier J-P & European Lung Cancer Working Party for the ILCSP (2008): Primary tumor standardized uptake value (SUVmax) measured on fluorodeoxyglucose positron emission tomography (FDG-PET) is of prognostic value for survival in non-small cell lung cancer (NSCLC): a systematic review and meta-analysis (MA) by the European Lung Cancer Working Party for the IASLC Lung Cancer Staging Project. *Journal of Thoracic Oncology: Official Publication of the International Association for the Study of Lung Cancer* **3**, 6-12. |
| Berghmans, 2006 | 886 | Berghmans T, Paesmans M, Mascaux C, Martin B, Meert AP, Haller A, Lafitte JJ & Sculier JP (2006): Thyroid transcription factor 1--a new prognostic factor in lung cancer: a meta-analysis. *Annals of Oncology* **17**, 1673-1676. |
| Berghmans, 2011 | 8434 | Berghmans T, Paesmans M & Sculier JP (2011): Prognostic factors in stage III non-small cell lung cancer: a review of conventional, metabolic and new biological variables. *Therapeutic Advances in Medical Oncology* **3**, 127-138. |
| Breen, 2008 | 791 | Breen D & Barlesi F (2008): The place of excision repair cross complementation 1 (ERCC1) in surgically treated non-small cell lung cancer. *European Journal of Cardio-Thoracic Surgery* **33**, 805-811. |
| Brundage, 2002 | 1051 | Brundage MD, Davies D & Mackillop WJ (2002): Prognostic factors in non-small cell lung cancer: a decade of progress. *Chest* **122**, 1037-1057. |
| Buttigliero, 2011 | 494 | Buttigliero C, Monagheddu C, Petroni P, Saini A, Dogliotti L, Ciccone G & Berruti A (2011): Prognostic role of vitamin d status and efficacy of vitamin D supplementation in cancer patients: a systematic review. *Oncologist* **16**, 1215-1227. |
| Carlson 2009 | 720 | Carlson JJ, Garrison LP, Ramsey SD & Veenstra DL (2009): Epidermal growth factor receptor genomic variation in NSCLC patients receiving tyrosine kinase inhibitor therapy: a systematic review and meta-analysis. *Journal of Cancer Research & Clinical Oncology* **135**, 1483-1493. |
| Carter, 2014 | 5362 | Carter GC, Barrett AM, Kaye JA, Liepa AM, Winfree KB & John WJ (2014) A comprehensive review of nongenetic prognostic and predictive factors influencing the heterogeneity of outcomes in advanced non-small-cell lung cancer In *Cancer Management and Research*, pp. 437-449. |
| Chen 2014 | 86 | Chen D, Shen C, Du H, Zhou Y & Che G (2014): Duplex value of caveolin-1 in non-small cell lung cancer: a meta analysis. *Familial Cancer* **13**, 449-457. |
| Chen, 2015 | 5751 | Chen S, Huang L, Sun K, Wu D, Li M, Zhong B, Chen M & Zhang S (2015): Enhancer of zeste homolog 2 as an independent prognostic marker for cancer: A meta-analysis. *PLoS ONE [Electronic Resource]* **10**. |
| Chen, 2010 | 635 | Chen S, Zhang J, Wang R, Luo X & Chen H (2010): The platinum-based treatments for advanced non-small cell lung cancer, is low/negative ERCC1 expression better than high/positive ERCC1 expression? A meta-analysis. *Lung Cancer* **70**, 63-70. |
| Chen, 2013 | 257 | Chen Y, Huang Y, Huang Y, Chen J, Wang S & Zhou J (2013a): The prognostic value of SOX2 expression in non-small cell lung cancer: a meta-analysis. *PLoS ONE [Electronic Resource]* **8**, e71140. |
| Chen, 2013 | 6663 | Chen Z, Xu L, Ye X, Shen S, Li Z, Niu X & Lu S (2013b): Polymorphisms of microRNA Sequences or Binding Sites and Lung Cancer: A Meta-Analysis and Systematic Review. *PLoS ONE [Electronic Resource]* **8**. |
| Choma, 2001 | 1085 | Choma D, Daures JP, Quantin X & Pujol JL (2001): Aneuploidy and prognosis of non-small-cell lung cancer: a meta-analysis of published data. *British Journal of Cancer* **85**, 14-22. |
| Christopoulos, 2013 | 5789 | Christopoulos AI (2013): Clinical Characteristics and Treatment Responses of Tuberculosis Complicating Lung Cancer: A Systematic Review. *International Journal of Life Science and Medical Research* **3**. |
| Dai, 2015 | 5508 | Dai J, Yang L, Wang J, Xiao Y & Ruan Q (2015): Prognostic Value of FOXM1 in Patients with Malignant Solid Tumor: A Meta-Analysis and System Review. *Disease Markers* **2015**. |
| De Geus-Oui, 2007 | 817 | de Geus-Oei L-F, van der Heijden HFM, Corstens FHM & Oyen WJG (2007): Predictive and prognostic value of FDG-PET in nonsmall-cell lung cancer: a systematic review. *Cancer* **110**, 1654-1664. |
| Deghaidy 2005 | 923 | Deghaidy AA, Nofal LM, Abd-Elmoneium SE & Mahdy NH (2005): Meta-analysis of survival models of lung cancer. *Journal of the Egyptian Public Health Association* **80**, 77-126. |
| Deng, 2015 | 5645 | Deng XF, Liu QX, Zhou D, Min JX & Dai JG (2015): Bone marrow micrometastasis is associated with both disease recurrence and poor survival in surgical patients with node-negative non-small-cell lung cancer: A meta-analysis. *Interactive Cardiovascular and Thoracic Surgery* **21**, 21-27. |
| Dimou, 2014 | 88 | Dimou A, Non L, Chae YK, Tester WJ & Syrigos KN (2014): MET gene copy number predicts worse overall survival in patients with non-small cell lung cancer (NSCLC); a systematic review and meta-analysis. *PLoS ONE [Electronic Resource]* **9**, e107677. |
| Ellis, 2010 | 7352 | Ellis PM, Blais N, Soulieres D, Ionescu DN, Liu G, Melosky B, Reiman T, Shepherd FA, Tsao M & Leighl NB (2010): A systematic review and consensus recommendations on the use of biomarkers in the treatment of non-small cell lung carcinoma (NSCLC). *Annals of Oncology* **21**, viii131. |
| Fan, 2008 | 777 | Fan J, Wang L, Jiang G-N, He W-X & Ding J-A (2008): The role of survivin on overall survival of non-small cell lung cancer, a meta-analysis of published literatures. *Lung Cancer* **61**, 91-96. |
| Florou, 2014 | 51 | Florou AN, Gkiozos ICH, Tsagouli SK, Souliotis KN & Syrigos KN (2014): Clinical significance of smoking cessation in subjects with cancer: a 30-year review. *Respiratory Care* **59**, 1924-1936. |
| Guo, 2014 | 134 | Guo B, Cen H, Tan X, Liu W & Ke Q (2014): Prognostic value of MET gene copy number and protein expression in patients with surgically resected non-small cell lung cancer: a meta-analysis of published literatures. *PLoS ONE [Electronic Resource]* **9**, e99399. |
| Gupta, 2010 | 610 | Gupta D & Lis CG (2010): Pretreatment serum albumin as a predictor of cancer survival: a systematic review of the epidemiological literature. *Nutrition Journal* **9**, 69. |
| He, 2013 | 6625 | He J, Zhang W, Zhang F, Wu Y, Zhu X, He X & Zhao Y (2013): Prognostic role of microRNA-155 in various carcinomas: Results from a meta-analysis. *Disease Markers* **34**, 379-386. |
| Hirsch, 2008 | 7515 | Hirsch FR, Spreafico A, Novello S, Wood MD, Simms L & Papotti M (2008): The prognostic and predictive role of histology in advanced non-small cell lung cancer: A literature review. *Journal of Thoracic Oncology* **3**, 1468-1481. |
| Hu, 2013 | 286 | Hu P, Liu W, Wang L, Yang M & Du J (2013): High circulating VEGF level predicts poor overall survival in lung cancer. *Journal of Cancer Research & Clinical Oncology* **139**, 1157-1167. |
| Huang, 2015 | 95 | Huang H, Wang T, Hu B & Pan C (2015): Visceral pleural invasion remains a size-independent prognostic factor in stage I non-small cell lung cancer. *Annals of Thoracic Surgery* **99**, 1130-1139. |
| Huang, 2013 | 234 | Huang J, Wang J, Wang K, Xu J, Huang J & Zhang T (2013a): Prognostic significance of circulating tumor cells in non-small-cell lung cancer patients: a meta-analysis.[Erratum appears in PLoS One. 2014;9(1). doi:10.1371/annotation/6633ed7f-a10c-4f6d-9d1d-9c1245822eb7 Note: Huang, Jianwei [corrected to Wang, Jianwei]]. *PLoS ONE [Electronic Resource]* **8**, e78070. |
| Huang, 2012 | 454 | Huang L-n, Wang D-s, Chen Y-q, Li W, Hu F-d, Gong B-l, Zhao C-L & Jia W (2012): Meta-analysis for cyclin E in lung cancer survival. *Clinica Chimica Acta* **413**, 663-668. |
| Huang, 2013 | 343 | Huang L-N, Wang D-S, Chen Y-Q, Zhao C-L, Gong B-L, Jiang A-B, Jia W & Hu F-D (2013b): Expression of survivin and patients survival in non-small cell lung cancer: a meta-analysis of the published studies. *Molecular Biology Reports* **40**, 917-924. |
| Hubner, 2011 | 483 | Hubner RA, Riley RD, Billingham LJ & Popat S (2011): Excision repair cross-complementation group 1 (ERCC1) status and lung cancer outcomes: a meta-analysis of published studies and recommendations. *PLoS ONE [Electronic Resource]* **6**, e25164. |
| Huncharek, 2000 | 1121 | Huncharek M, Kupelnick B, Geschwind JF & Caubet JF (2000): Prognostic significance of p53 mutations in non-small cell lung cancer: a meta-analysis of 829 cases from eight published studies. *Cancer Letters* **153**, 219-226. |
| Huncharek, 1999 | 1133 | Huncharek M, Muscat J & Geschwind JF (1999): K-ras oncogene mutation as a prognostic marker in non-small cell lung cancer: a combined analysis of 881 cases. *Carcinogenesis* **20**, 1507-1510. |
| Huo, 2015 | 5784 | Huo W, Du M, Pan X, Zhu X & Li Z (2015): Prognostic value of ALDH1 expression in lung cancer: A meta-analysis. *International Journal of Clinical and Experimental Medicine* **8**, 2045-2051. |
| Im, 2015 | 14 | Im H-J, Pak K, Cheon GJ, Kang KW, Kim S-J, Kim I-J, Chung J-K, Kim EE & Lee DS (2015): Prognostic value of volumetric parameters of (18)F-FDG PET in non-small-cell lung cancer: a meta-analysis. *European Journal of Nuclear Medicine & Molecular Imaging* **42**, 241-251. |
| Jiang, 2014 | 144 | Jiang H, Shao W & Zhao W (2014a): VEGF-C in non-small cell lung cancer: meta-analysis. *Clinica Chimica Acta* **427**, 94-99. |
| Jiang, 2013 | 312 | Jiang H, Wang J & Zhao W (2013): Cox-2 in non-small cell lung cancer: a meta-analysis. *Clinica Chimica Acta* **419**, 26-32. |
| Jiang, 2014 | 140 | Jiang H, Zhao W & Shao W (2014b): Prognostic value of CD44 and CD44v6 expression in patients with non-small cell lung cancer: meta-analysis. *Tumour Biology* **35**, 7383-7389. |
| Jiang, 2012 | 424 | Jiang J, Liang X, Zhou X, Huang R, Chu Z & Zhan Q (2012): ERCC1 expression as a prognostic and predictive factor in patients with non-small cell lung cancer: a meta-analysis. *Molecular Biology Reports* **39**, 6933-6942. |
| Jiang, 2015 | 5 | Jiang L, He J, Shi X, Shen J, Liang W, Yang C & He J (2015a): Prognosis of synchronous and metachronous multiple primary lung cancers: systematic review and meta-analysis. *Lung Cancer* **87**, 303-310. |
| Jiang, 2015 | 4951 | Jiang L, Liang WH, Shen JF, Chen XF, Shi XS, He JX & Yang CL (2015b): The Impact of Visceral Pleural Invasion in Node-Negative Non-small Cell Lung Cancer A Systematic Review and Meta-analysis. *Chest* **148**, 903-911. |
| Jiang, 2015 | 4988 | Jiang T, Gao GH, Fan GX, Li M & Zhou CC (2015c): FGFR1 amplification in lung squamous cell carcinoma: A systematic review with meta-analysis. *Lung Cancer* **87**, 1-7. |
| Jin, 2014 | 67 | Jin Y, Sun Y, Shi X, Zhao J, Shi L & Yu X (2014): Prognostic value of circulating C-reactive protein levels in patients with non-small cell lung cancer: a systematic review with meta-analysis. *Journal of Cancer Research & Therapeutics* **10 Suppl**, C160-166. |
| Jing, 2015 | 5605 | Jing X, Huang C, Zhou H, Li C, Fan L, Chen J, Zhang G, Liu Y, Cui Z, Qi D & Ma J (2015): Association between serum C-reactive protein value and prognosis of patients with non-small cell lung cancer: A meta-analysis. *International Journal of Clinical and Experimental Medicine* **8**, 10633-10639. |
| Kilvaer, 2015 | 5533 | Kilvaer TK, Paulsen EE, Hald SM, Wilsgaard T, Bremnes RM, Busund LT & Donnem T (2015): Lymphangiogenic markers and their impact on nodal metastasis and survival in non-small cell lung cancer - A structured review with meta-analysis. *PLoS ONE [Electronic Resource]* **10**. |
| Knez, 2011 | 525 | Knez L, Sodja E, Kern I, Kosnik M & Cufer T (2011): Predictive value of multidrug resistance proteins, topoisomerases II and ERCC1 in small cell lung cancer: a systematic review. *Lung Cancer* **72**, 271-279. |
| Kong, 2014 | 157 | Kong Q, Li P, Tian Q & Ha M-W (2014): Role of MDM2 T309G polymorphism in susceptibility and prognosis of nonsmall cell lung cancer: a meta-analysis. *Genetic Testing & Molecular Biomarkers* **18**, 357-365. |
| Li, 2013 | 272 | Li C, Lu H-J, Na F-F, Deng L, Xue J-X, Wang J-W, Wang Y-Q, Li Q-L & Lu Y (2013): Prognostic role of hypoxic inducible factor expression in non-small cell lung cancer: a meta-analysis. *Asian Pacific Journal of Cancer Prevention: Apjcp* **14**, 3607-3612. |
| Li, 2015 | 4976 | Li L, Liu D, Qiu ZX, Zhao S, Zhang L & Li WM (2015a): The Prognostic Role of mTOR and P-mTOR for Survival in Non-Small Cell Lung Cancer: A Systematic Review and Meta-Analysis. *PLoS ONE [Electronic Resource]* **10**. |
| Li, 2015 | 5436 | Li W, Tse LA & Wang F (2015b): Prognostic value of estrogen receptors mRNA expression in non-small cell lung cancer: A systematic review and meta-analysis. *Steroids* **104**, 129-136. |
| Liang, 2015 | 5708 | Liang JX, Gao W, Liang Y & Zhou XM (2015): Chemokine receptor CXCR4 expression and lung cancer prognosis: A meta-analysis. *International Journal of Clinical and Experimental Medicine* **8**, 5163-5174. |
| Liang, 2014 | 183 | Liang Y, Guo S & Zhou Q (2014): Prognostic value of matrix metalloproteinase-7 expression in patients with non-small cell lung cancer. *Tumour Biology* **35**, 3717-3724. |
| Liao 2014 | 76 | Liao C, Yu Z, Guo W, Liu Q, Wu Y, Li Y & Bai L (2014): Prognostic value of circulating inflammatory factors in non-small cell lung cancer: a systematic review and meta-analysis. *Cancer Biomarkers: Section A of Disease Markers* **14**, 469-481. |
| Lim, 2010 | 662 | Lim E, Clough R, Goldstraw P, Edmonds L, Aokage K, Yoshida J, Nagai K, Shintani Y, Ohta M, Okumura M, Iwasaki T, Yasumitsu T, Okada M, Mimura T, Tsubota N, Nakagawa T, Okumura N, Satoh Y, Okumura S, Nakagawa K, Higashiyama M, Kodama K, Riquet M, Vicidomini G, Santini M, Kotoulas C, Hsu J-Y, Chen C-Y & International Pleural Lavage Cytology C (2010): Impact of positive pleural lavage cytology on survival in patients having lung resection for non-small-cell lung cancer: An international individual patient data meta-analysis. *Journal of Thoracic & Cardiovascular Surgery* **139**, 1441-1446. |
| Liu 2013 | 332 | Liu H-b, Wu Y, Lv T-f, Yao Y-w, Xiao Y-y, Yuan D-m & Song Y (2013a): Skin rash could predict the response to EGFR tyrosine kinase inhibitor and the prognosis for patients with non-small cell lung cancer: a systematic review and meta-analysis. *PLoS ONE [Electronic Resource]* **8**, e55128. |
| Liu, 2015 | 5680 | Liu J, Dong M, Sun X & Xing L (2015a): Prognostic value of 18F-FDG PET uptake in surgically resected non-small cell lung cancer: A systemic review and metaanalysis. *Journal of Nuclear Medicine* **56**. |
| Liu, 2015 | 5677 | Liu K, Bao C, Yao N, Miao C, Varlotto J, Sun Q & Sun X (2015b): Expression of CXCR4 and non-small cell lung cancer prognosis: A meta-analysis. *International Journal of Clinical and Experimental Medicine* **8**, 7435-7445. |
| Liu, 2010 | 612 | Liu L, Shao X, Gao W, Bai J, Wang R, Huang P, Yin Y, Liu P & Shu Y (2010): The role of human epidermal growth factor receptor 2 as a prognostic factor in lung cancer: a meta-analysis of published data. *Journal of Thoracic Oncology: Official Publication of the International Association for the Study of Lung Cancer* **5**, 1922-1932. |
| Liu, 2015 | 36 | Liu Q, Yu Z, Xiang Y, Wu N, Wu L, Xu B, Wang L, Yang P, Li Y & Bai L (2015c): Prognostic and predictive significance of thymidylate synthase protein expression in non-small cell lung cancer: a systematic review and meta-analysis. *Cancer Biomarkers: Section A of Disease Markers* **15**, 65-78. |
| Liu, 2015 | 5525 | Liu Y, Gu X, Lin Q, Tian T, Shao L, Yuan C, Zhang B & Fan K (2015d): Prognostic significance of osteopontin in patients with non-small cell lung cancer: Results from a meta-analysis. *International Journal of Clinical and Experimental Medicine* **8**, 12765-12773. |
| Liu, 2013 | 239 | Liu Y, Yin T-J, Zhou R, Zhou S, Fan L & Zhang R-G (2013b): Expression of thymidylate synthase predicts clinical outcomes of pemetrexed-containing chemotherapy for non-small-cell lung cancer: a systemic review and meta-analysis. *Cancer Chemotherapy & Pharmacology* **72**, 1125-1132. |
| Lou-Quian, 2013 | 334 | Lou-Qian Z, Rong Y, Ming L, Xin Y, Feng J & Lin X (2013): The prognostic value of epigenetic silencing of p16 gene in NSCLC patients: a systematic review and meta-analysis. *PLoS ONE [Electronic Resource]* **8**, e54970. |
| Luan, 2014 | 171 | Luan H, Ye F, Wu L, Zhou Y & Jiang J (2014): Perioperative blood transfusion adversely affects prognosis after resection of lung cancer: a systematic review and a meta-analysis. *BMC Surgery* **14**, 34. |
| Luo, 2015 | 58 | Luo H, Qiao L, Liang N & Zhang J (2015a): Risk factors for recurrence in patients with resected N1 non-small cell lung cancer - a systematic review and meta-analysis. *Journal of Buon* **20**, 791-799. |
| Luo, 2014 | 107 | Luo Z, Wu R-R, Lv L, Li P, Zhang L-Y, Hao Q-L & Li W (2014): Prognostic value of CD44 expression in non-small cell lung cancer: a systematic review. *International Journal of Clinical & Experimental Pathology* **7**, 3632-3646. |
| Luo, 2015 | 5651 | Luo Z, Wu R, Jiang Y, Qiu Z, Chen W & Li W (2015b): Overexpression of estrogen receptor beta is a prognostic marker in non-small cell lung cancer: A meta-analysis. *International Journal of Clinical and Experimental Medicine* **8**, 8686-8697. |
| Ma, 2015 | 5676 | Ma JY, Yan HJ & Gu W (2015): Association between BIM deletion polymorphism and clinical outcome of EGFR-mutated NSCLC patient with EGFR-TKI therapy: A meta-analysis. *Journal of Cancer Research and Therapeutics* **11**, 397-402. |
| Ma, 2012 | 395 | Ma X-L, Liu L, Liu X-X, Li Y, Deng L, Xiao Z-L, Liu Y-T, Shi H-S & Wei Y-q (2012a): Prognostic role of microRNA-21 in non-small cell lung cancer: a meta-analysis. *Asian Pacific Journal of Cancer Prevention: Apjcp* **13**, 2329-2334. |
| Ma, 2012 | 401 | Ma X-L, Xiao Z-L, Liu L, Liu X-X, Nie W, Li P, Chen N-Y & Wei Y-Q (2012b): Meta-analysis of circulating tumor cells as a prognostic marker in lung cancer. *Asian Pacific Journal of Cancer Prevention: Apjcp* **13**, 1137-1144. |
| Ma, 2014 | 190 | Ma X, Li Y, Zhang J, Huang J & Liu L (2014): Prognostic role of D-dimer in patients with lung cancer: a meta-analysis. *Tumour Biology* **35**, 2103-2109. |
| Marchevsky, 2010 | 625 | Marchevsky AM, Gupta R, Kusuanco D, Mirocha J & McKenna RJ, Jr. (2010): The presence of isolated tumor cells and micrometastases in the intrathoracic lymph nodes of patients with lung cancer is not associated with decreased survival. *Human Pathology* **41**, 1536-1543. |
| Martin, 2003 | 1024 | Martin B, Paesmans M, Berghmans T, Branle F, Ghisdal L, Mascaux C, Meert AP, Steels E, Vallot F, Verdebout JM, Lafitte JJ & Sculier JP (2003): Role of Bcl-2 as a prognostic factor for survival in lung cancer: a systematic review of the literature with meta-analysis. *British Journal of Cancer* **89**, 55-64. |
| Martin, 2004 | 969 | Martin B, Paesmans M, Mascaux C, Berghmans T, Lothaire P, Meert AP, Lafitte JJ & Sculier JP (2004): Ki-67 expression and patients survival in lung cancer: systematic review of the literature with meta-analysis. *British Journal of Cancer* **91**, 2018-2025. |
| Mascaux, 2005 | 961 | Mascaux C, Iannino N, Martin B, Paesmans M, Berghmans T, Dusart M, Haller A, Lothaire P, Meert AP, Noel S, Lafitte JJ & Sculier JP (2005): The role of RAS oncogene in survival of patients with lung cancer: a systematic review of the literature with meta-analysis. *British Journal of Cancer* **92**, 131-139. |
| Mascaux, 2006 | 899 | Mascaux C, Martin B, Paesmans M, Berghmans T, Dusart M, Haller A, Lothaire P, Meert AP, Lafitte JJ & Sculier JP (2006): Has Cox-2 a prognostic role in non-small-cell lung cancer? A systematic review of the literature with meta-analysis of the survival results. *British Journal of Cancer* **95**, 139-145. |
| Meert, 2002 | 1045 | Meert AP, Martin B, Delmotte P, Berghmans T, Lafitte JJ, Mascaux C, Paesmans M, Steels E, Verdebout JM & Sculier JP (2002a): The role of EGF-R expression on patient survival in lung cancer: a systematic review with meta-analysis. *European Respiratory Journal* **20**, 975-981. |
| Meert, 2002 | 1050 | Meert AP, Martin B, Paesmans M, Berghmans T, Mascaux C, Verdebout JM, Delmotte P, Lafitte JJ & Sculier JP (2003): The role of HER-2/neu expression on the survival of patients with lung cancer: a systematic review of the literature. *British Journal of Cancer* **89**, 959-965. |
| Meert, 2003 | 1019 | Meert AP, Paesmans M, Martin B, Delmotte P, Berghmans T, Verdebout JM, Lafitte JJ, Mascaux C & Sculier JP (2002b): The role of microvessel density on the survival of patients with lung cancer: a systematic review of the literature with meta-analysis. *British Journal of Cancer* **87**, 694-701. |
| Mei, 2013 | 319 | Mei XD, Su H, Song J & Dong L (2013): Prognostic significance of beta-catenin expression in patients with non-small cell lung cancer: a meta-analysis. *Bioscience Trends* **7**, 42-49. |
| Meng, 2013 | 287 | Meng D, Yuan M, Li X, Chen L, Yang J, Zhao X, Ma W & Xin J (2013): Prognostic value of K-RAS mutations in patients with non-small cell lung cancer: a systematic review with meta-analysis. *Lung Cancer* **81**, 1-10. |
| Miao, 2012 | 6341 | Miao XH, Yao YW, Yuan DM, Lv YL, Zhan P, Lv TF, Liu HB & Song Y (2012): Prognostic value of the ratio of ground glass opacity on computed tomography in small lung adenocarcinoma: A meta-analysis. *Journal of Thoracic Disease* **4**, 265-271. |
| Mitsudomi, 2000 | 1110 | Mitsudomi T, Hamajima N, Ogawa M & Takahashi T (2000): Prognostic significance of p53 alterations in patients with non-small cell lung cancer: a meta-analysis. *Clinical Cancer Research* **6**, 4055-4063. |
| Mollberg, 2014 | 196 | Mollberg NM, Bennette C, Howell E, Backhus L, Devine B & Ferguson MK (2014): Lymphovascular invasion as a prognostic indicator in stage I non-small cell lung cancer: a systematic review and meta-analysis. *Annals of Thoracic Surgery* **97**, 965-971. |
| Montazeri, 2009 | 703 | Montazeri A (2009): Quality of life data as prognostic indicators of survival in cancer patients: an overview of the literature from 1982 to 2008. *Health & Quality of Life Outcomes* **7**, 102. |
| Na, 2014 | 133 | Na F, Wang J, Li C, Deng L, Xue J & Lu Y (2014): Primary tumor standardized uptake value measured on F18-Fluorodeoxyglucose positron emission tomography is of prediction value for survival and local control in non-small-cell lung cancer receiving radiotherapy: meta-analysis. *Journal of Thoracic Oncology: Official Publication of the International Association for the Study of Lung Cancer* **9**, 834-842. |
| Nair, 2009 | 706 | Nair VS, Krupitskaya Y & Gould MK (2009): Positron emission tomography 18F-fluorodeoxyglucose uptake and prognosis in patients with surgically treated, stage I non-small cell lung cancer: a systematic review. *Journal of Thoracic Oncology: Official Publication of the International Association for the Study of Lung Cancer* **4**, 1473-1479. |
| Nakamura, 2011 | 481 | Nakamura H, Ando K, Shinmyo T, Morita K, Mochizuki A, Kurimoto N & Tatsunami S (2011): Female gender is an independent prognostic factor in non-small-cell lung cancer: a meta-analysis. *Annals of Thoracic & Cardiovascular Surgery* **17**, 469-480. |
| Nakamura, 2005 | 952 | Nakamura H, Kawasaki N, Taguchi M & Kabasawa K (2005): Association of HER-2 overexpression with prognosis in nonsmall cell lung carcinoma: a metaanalysis. *Cancer* **103**, 1865-1873. |
| Nakamura, 2006 | 918 | Nakamura H, Kawasaki N, Taguchi M & Kabasawa K (2006): Survival impact of epidermal growth factor receptor overexpression in patients with non-small cell lung cancer: a meta-analysis. *Thorax* **61**, 140-145. |
| Neal, 2015 | 8441 | Neal RD, Tharmanathan P, France B, Din NU, Cotton S, Fallon-Ferguson J, Hamilton W, Hendry A, Hendry M, Lewis R, Macleod U, Mitchell ED, Pickett M, Rai T, Shaw K, Stuart N, Torring ML, Wilkinson C, Williams B, Williams N & Emery J (2015): Is increased time to diagnosis and treatment in symptomatic cancer associated with poorer outcomes? Systematic review. *British Journal of Cancer*, S92-S107. |
| Olsson 2009 | 722 | Olsson JK, Schultz EM & Gould MK (2009): Timeliness of care in patients with lung cancer: a systematic review. *Thorax* **64**, 749-756. |
| Paesmans, 2010 | 667 | Paesmans M, Berghmans T, Dusart M, Garcia C, Hossein-Foucher C, Lafitte J-J, Mascaux C, Meert A-P, Roelandts M, Scherpereel A, Terrones Munoz V, Sculier J-P, European Lung Cancer Working P & on behalf of the ILCSP (2010): Primary tumor standardized uptake value measured on fluorodeoxyglucose positron emission tomography is of prognostic value for survival in non-small cell lung cancer: update of a systematic review and meta-analysis by the European Lung Cancer Working Party for the International Association for the Study of Lung Cancer Staging Project. *Journal of Thoracic Oncology: Official Publication of the International Association for the Study of Lung Cancer* **5**, 612-619. |
| Pan, 5774 | 5774 | Pan ZK, Ye F, Wu X, An HX & Wu JX (2015): Clinicopathological and prognostic significance of programmed cell death ligand1 (PD-L1) expression in patients with non-small cell lung cancer: A meta-analysis. *Journal of Thoracic Disease* **7**, 462-470. |
| Parsons, 2010 | 695 | Parsons A, Daley A, Begh R & Aveyard P (2010): Influence of smoking cessation after diagnosis of early stage lung cancer on prognosis: systematic review of observational studies with meta-analysis. *BMJ* **340**, b5569. |
| Peng, 2014 | 5929 | Peng B, Wang YH, Huang Z, Feng SJ & Wang YS (2014): Prognostic significance of Osteopontin in patients with lung cancer: A meta-analysis. *International Journal of Clinical and Experimental Medicine* **7**, 4616-4626. |
| Peng, 2015 | 5752 | Peng B, Wang YH, Liu YM & Ma LX (2015): Prognostic significance of the neutrophil to lymphocyte ratio in patients with non-small cell lung cancer: A systemic review and meta-analysis. *International Journal of Clinical and Experimental Medicine* **8**, 3098-3106. |
| Peng, 2012 | 437 | Peng W-J, Zhang J-Q, Wang B-X, Pan H-F, Lu M-M & Wang J (2012): Prognostic value of matrix metalloproteinase 9 expression in patients with non-small cell lung cancer. *Clinica Chimica Acta* **413**, 1121-1126. |
| Petrelli, 2012 | 389 | Petrelli F, Borgonovo K, Cabiddu M, Lonati V & Barni S (2012): Relationship between skin rash and outcome in non-small-cell lung cancer patients treated with anti-EGFR tyrosine kinase inhi |
| Prades, 2015 | 5807 | Prades J, Remue E, van Hoof E & Borras JM (2015): Is it worth reorganising cancer services on the basis of multidisciplinary teams (MDTs)? A systematic review of the objectives and organisation of MDTs and their impact on patient outcomes. *Health Policy* **119**, 464-474. |
| Pujol, 2004 | 993 | Pujol JL, Molinier O, Ebert W, Daures JP, Barlesi F, Buccheri G, Paesmans M, Quoix E, Moro-Sibilot D, Szturmowicz M, Brechot JM, Muley T & Grenier J (2004): CYFRA 21-1 is a prognostic determinant in non-small-cell lung cancer: results of a meta-analysis in 2063 patients. *British Journal of Cancer* **90**, 2097-2105. |
| Qian, 2010 | 657 | Qian Q, Wang Q, Zhan P, Peng L, Wei S-Z, Shi Y & Song Y (2010): The role of matrix metalloproteinase 2 on the survival of patients with non-small cell lung cancer: a systematic review with meta-analysis. *Cancer Investigation* **28**, 661-669. |
| Qin, 2013 | 229 | Qin Q, Zhang C, Yang X, Zhu H, Yang B, Cai J, Cheng H, Ma J, Lu J, Zhan L, Liu J, Liu Z, Xu L & Sun X (2013): Polymorphisms in XPD gene could predict clinical outcome of platinum-based chemotherapy for non-small cell lung cancer patients: a meta-analysis of 24 studies. *PLoS ONE [Electronic Resource]* **8**, e79864. |
| Qiu, 2013 | 242 | Qiu M, Xu L, Yang X, Ding X, Hu J, Jiang F, Xu L & Yin R (2013a): XRCC3 Thr241Met is associated with response to platinum-based chemotherapy but not survival in advanced non-small cell lung cancer. *PLoS ONE [Electronic Resource]* **8**, e77005. |
| Qiu, 2013 | 221 | Qiu Q, Zhi-Xin, Zhang K, Qiu X-S, Zhou M & Li W-M (2013b): The prognostic value of phosphorylated AKT expression in non-small cell lung cancer: a meta-analysis. *PLoS ONE [Electronic Resource]* **8**, e81451. |
| Qiu, 2015 | 5592 | Qiu ZX, Zhao S, Li L & Li WM (2015): Prognostic value and clinicopathological significance of epithelial cadherin expression in non-small cell lung cancer. *Thoracic Cancer* **6**, 589-596. |
| Qu, 2013 | 233 | Qu H, Li R, Liu Z, Zhang J & Luo R (2013): Prognostic value of cancer stem cell marker CD133 expression in non-small cell lung cancer: a systematic review. *International Journal of Clinical & Experimental Pathology* **6**, 2644-2650. |
| Quinton, 2011 | 6781 | Quinton C & Ellis PM (2011): An evidence-based approach to the use of predictive biomarkers in the treatment of non- small cell lung cancer (NSCLC). *Cancers* **3**, 3506-3524. |
| Ren, 2013 | 249 | Ren W, Mi D, Yang K, Cao N, Tian J, Li Z & Ma B (2013): The expression of hypoxia-inducible factor-1alpha and its clinical significance in lung cancer: a systematic review and meta-analysis. *Swiss Medical Weekly* **143**, w13855. |
| Roth, 2011 | 477 | Roth JA & Carlson JJ (2011): Prognostic role of ERCC1 in advanced non-small-cell lung cancer: a systematic review and meta-analysis. *Clinical Lung Cancer* **12**, 393-401. |
| Salah, 2012 | 467 | Salah S, Tanvetyanon T & Abbasi S (2012): Metastatectomy for extra-cranial extra-adrenal non-small cell lung cancer solitary metastases: systematic review and analysis of reported cases. *Lung Cancer* **75**, 9-14. |
| Saso, 2012 | 427 | Saso S, Rao C, Ashrafian H, Ghaem-Maghami S, Darzi A & Athanasiou T (2012): Positive pre-resection pleural lavage cytology is associated with increased risk of lung cancer recurrence in patients undergoing surgical resection: a meta-analysis of 4450 patients. *Thorax* **67**, 526-532. |
| Shao, 2015 | 5618 | Shao W, Chen H & He J (2015): The role of SOX-2 on the survival of patients with non-small cell lung cancer. *Journal of Thoracic Disease* **7**, 1113-1118. |
| Shao, 2014 | 122 | Shao Y, Geng Y, Gu W, Huang J, Ning Z & Pei H (2014): Prognostic significance of microRNA-375 downregulation in solid tumors: a meta-analysis. *Disease Markers* **2014**, 626185. |
| Shen, 2014 | 62 | Shen W, Xi H, Zhang K, Cui J, Li J, Wang N, Wei B & Chen L (2014): Prognostic role of EphA2 in various human carcinomas: a meta-analysis of 23 related studies. *Growth Factors* **32**, 247-253. |
| Shen, 2013 | 266 | Shen X-y, Lu F-z, Wu Y, Zhao L-t & Lin Z-f (2013): XRCC3 Thr241Met polymorphism and clinical outcomes of NSCLC patients receiving platinum-based chemotherapy: a systematic review and meta-analysis. *PLoS ONE [Electronic Resource]* **8**, e69553. |
| Slatore, 2010 | 621 | Slatore CG, Au DH, Gould MK & American Thoracic Society Disparities in Healthcare G (2010): An official American Thoracic Society systematic review: insurance status and disparities in lung cancer practices and outcomes. *American Journal of Respiratory & Critical Care Medicine* **182**, 1195-1205. |
| Soo, 2011 | 520 | Soo RA, Loh M, Mok TS, Ou S-HI, Cho B-C, Yeo W-L, Tenen DG & Soong R (2011): Ethnic differences in survival outcome in patients with advanced stage non-small cell lung cancer: results of a meta-analysis of randomized controlled trials. *Journal of Thoracic Oncology: Official Publication of the International Association for the Study of Lung Cancer* **6**, 1030-1038. |
| Steels, 2001 | 5212 | Steels E, Paesmans B, Berghmans T, Branle F, Lemaitre F, Mascaux C, Meert AP, Vallot F, Lafitte JJ & Sculier JP (2001): Role of p53 as a prognostic factor for survival in lung cancer: a systematic review of the literature with a meta-analysis. *European Respiratory Journal* **18**, 705-719. |
| Sun, 2013 | 6459 | Sun G, Hu W, Lu Y, Wang Y, Zhai H & Cui D (2013): Correlation between survivin expression and clinicopathological characteristics in patients with non-small cell lung cancer. *International Medical Journal* **20**, 571-578. |
| Sun, 2015 | 5469 | Sun G, Xue L, Wang M & Zhao X (2015): Lymph node ratio is a prognostic factor for non-small cell lung cancer. *Oncotarget* **6**, 33912-33918. |
| Tanvetyanon, 2015 | 23 | Tanvetyanon T, Finley DJ, Fabian T, Riquet M, Voltolini L, Kocaturk C, Bryant A & Robinson L (2015): Prognostic nomogram to predict survival after surgery for synchronous multiple lung cancers in multiple lobes. *Journal of Thoracic Oncology: Official Publication of the International Association for the Study of Lung Cancer* **10**, 338-345. |
| Tanvetyanon, 2013 | 320 | Tanvetyanon T, Finley DJ, Fabian T, Riquet M, Voltolini L, Kocaturk C, Fulp WJ, Cerfolio RJ, Park BJ & Robinson LA (2013): Prognostic factors for survival after complete resections of synchronous lung cancers in multiple lobes: pooled analysis based on individual patient data. *Annals of Oncology* **24**, 889-894. |
| Tanvetyanon, 2008 | 799 | Tanvetyanon T, Robinson LA, Schell MJ, Strong VE, Kapoor R, Coit DG & Bepler G (2008): Outcomes of adrenalectomy for isolated synchronous versus metachronous adrenal metastases in non-small-cell lung cancer: a systematic review and pooled analysis. *Journal of Clinical Oncology* **26**, 1142-1147. |
| Tian, 2015 | 4978 | Tian XL & Xu GX (2015): Clinical value of lncRNA MALAT1 as a prognostic marker in human cancer: systematic review and meta-analysis. *BMJ Open* **5**. |
| Tong, 2011 | 486 | Tong J, Sun X, Cheng H, Zhao D, Ma J, Zhen Q, Cao Y, Zhu H & Bai J (2011): Expression of p16 in non-small cell lung cancer and its prognostic significance: a meta-analysis of published literatures. *Lung Cancer* **74**, 155-163. |
| Trivella, 2007 | 846 | Trivella M, Pezzella F, Pastorino U, Harris AL, Altman DG & Prognosis In Lung Cancer Collaborative Study G (2007): Microvessel density as a prognostic factor in non-small-cell lung carcinoma: a meta-analysis of individual patient data.[Erratum appears in Lancet Oncol. 2007 Aug;8(8):670]. *Lancet Oncology* **8**, 488-499. |
| Vansteenkiste, 2004 | 982 | Vansteenkiste J, Fischer BM, Dooms C & Mortensen J (2004): Positron-emission tomography in prognostic and therapeutic assessment of lung cancer: systematic review. *Lancet Oncology* **5**, 531-540. |
| von Meyenfeldt, 2012 | 414 | von Meyenfeldt EM, Gooiker GA, van Gijn W, Post PN, van de Velde CJH, Tollenaar RAEM, Klomp HM & Wouters MWJM (2012): The relationship between volume or surgeon specialty and outcome in the surgical treatment of lung cancer: a systematic review and meta-analysis. *Journal of Thoracic Oncology: Official Publication of the International Association for the Study of Lung Cancer* **7**, 1170-1178. |
| Wang 2015 | 103 | Wang A, Wang HY, Liu Y, Zhao MC, Zhang HJ, Lu ZY, Fang YC, Chen XF & Liu GT (2015a): The prognostic value of PD-L1 expression for non-small cell lung cancer patients: a meta-analysis. *European Journal of Surgical Oncology* **41**, 450-456. |
| Wang 2015 | 5537 | Wang F, Zhou J, Zhang Y, Wang Y, Cheng L, Bai Y & Ma H (2015b): The value of microRNA-155 as a prognostic factor for survival in non-small cell lung cancer: A meta-analysis. *PLoS ONE [Electronic Resource]* **10**. |
| Wang, 2014 | 129 | Wang H, Huang J, Yu X, Han S, Yan X, Sun S & Zhu X (2014a): Different efficacy of EGFR tyrosine kinase inhibitors and prognosis in patients with subtypes of EGFR-mutated advanced non-small cell lung cancer: a meta-analysis.[Erratum appears in J Cancer Res Clin Oncol. 2014 Nov;140(11):1911]. *Journal of Cancer Research & Clinical Oncology* **140**, 1901-1909. |
| Wang, 2011 | 469 | Wang J, Chen J, Chen X, Wang B, Li K & Bi J (2011a): Blood vessel invasion as a strong independent prognostic indicator in non-small cell lung cancer: a systematic review and meta-analysis. *PLoS ONE [Electronic Resource]* **6**, e28844. |
| Wang, 2012 | 448 | Wang J, Li K, Wang B & Bi J (2012a): Lymphatic microvessel density as a prognostic factor in non-small cell lung carcinoma: a meta-analysis of the literature. *Molecular Biology Reports* **39**, 5331-5338. |
| Wang, 2011 | 534 | Wang J, Wang B, Chen X & Bi J (2011b): The prognostic value of RASSF1A promoter hypermethylation in non-small cell lung carcinoma: a systematic review and meta-analysis. *Carcinogenesis* **32**, 411-416. |
| Wang, 2012 | 356 | Wang J, Wang B, Zhao W, Guo Y, Chen H, Chu H, Liang X & Bi J (2012b): Clinical significance and role of lymphatic vessel invasion as a major prognostic implication in non-small cell lung cancer: a meta-analysis. *PLoS ONE [Electronic Resource]* **7**, e52704. |
| Wang, 2014 | 191 | Wang L, Wang R, Pan Y, Sun Y, Zhang J & Chen H (2014b): The pemetrexed-containing treatments in the non-small cell lung cancer is -/low thymidylate synthase expression better than +/high thymidylate synthase expression: a meta-analysis. *BMC Cancer* **14**, 205. |
| Wang, 2014 | 187 | Wang Q, Hu D-f, Rui Y, Jiang A-b, Liu Z-l & Huang L-n (2014c): Prognosis value of HIF-1alpha expression in patients with non-small cell lung cancer. *Gene* **541**, 69-74. |
| Wang, 2013 | 247 | Wang T, Chuan Pan C, Rui Yu J, Long Y, Hong Cai X, De Yin X, Qiong Hao L & Li Luo L (2013a): Association between TYMS expression and efficacy of pemetrexed-based chemotherapy in advanced non-small cell lung cancer: a meta-analysis. *PLoS ONE [Electronic Resource]* **8**, e74284. |
| Wang, 2014 | 180 | Wang T, Luo L, Huang H, Yu J, Pan C, Cai X, Hu B & Yin X (2014d): Perioperative blood transfusion is associated with worse clinical outcomes in resected lung cancer. *Annals of Thoracic Surgery* **97**, 1827-1837. |
| Wang, 2014 | 151 | Wang W, Chen Y, Deng J, Zhou J, Zhou Y, Wang S & Zhou J (2014e): The prognostic value of CD133 expression in non-small cell lung cancer: a meta-analysis. *Tumour Biology* **35**, 9769-9775. |
| Wang, 2014 | 150 | Wang X-B, Li J & Han Y (2014f): Prognostic significance of preoperative serum carcinoembryonic antigen in non-small cell lung cancer: a meta-analysis. *Tumour Biology* **35**, 10105-10110. |
| Wang, 2013 | 268 | Wang Y, Li J, Tong L, Zhang J, Zhai A, Xu K, Wei L & Chu M (2013b): The prognostic value of miR-21 and miR-155 in non-small-cell lung cancer: a meta-analysis. *Japanese Journal of Clinical Oncology* **43**, 813-820. |
| Wang, 2015 | 5514 | Wang Z, Wang B, Guo H, Shi G & Hong X (2015c): Clinicopathological significance and potential drug target of T-cadherin in NSCLC. *Drug Design, Development and Therapy* **9**, 207-216. |
| Wei 2015 | 4957 | Wei D, Peng JJ, Gao H, Zhang T, Tan Y & Hu YH (2015): ALDH1 Expression and the Prognosis of Lung Cancer: A Systematic Review and Meta-Analysis. *Heart Lung and Circulation* **24**, 780-788. |
| Wen 2015 | 5684 | Wen S, Zhou W, Li CM, Hu J, Hu XM, Chen P, Shao GL & Guo WH (2015): Ki-67 as a prognostic marker in early-stage non-small cell lung cancer in Asian patients: A meta-analysis of published studies involving 32 studies. *BMC Cancer* **15**. |
| Wu 2015 | 85510 | Wu D, Wu P, Zhao L, Huang L, Zhang Z, Zhao S & Huang J (2015): NF-kappaB expression and outcomes in solid tumors: A systematic review and meta-analysis. *Medicine (United States)* **94**. |
| Wu 2014 | 30 | Wu H, Qi X-w, Yan G-n, Zhang Q-b, Xu C & Bian X-w (2014a): Is CD133 expression a prognostic biomarker of non-small-cell lung cancer? A systematic review and meta-analysis. *PLoS ONE [Electronic Resource]* **9**, e100168. |
| Wu 2012 | 400 | Wu J, Liu J, Zhou Y, Ying J, Zou H, Guo S, Wang L, Zhao N, Hu J, Lu D, Jin L, Li Q & Wang J-C (2012a): Predictive value of XRCC1 gene polymorphisms on platinum-based chemotherapy in advanced non-small cell lung cancer patients: a systematic review and meta-analysis. *Clinical Cancer Research* **18**, 3972-3981. |
| Wu 2014 | 125 | Wu K-P, Li Q, Lin F-X, Li J, Wu L-M, Li W & Yang Q-Z (2014b): MT1-MMP is not a good prognosticator of cancer survival: evidence from 11 studies. *Tumour Biology* **35**, 12489-12495. |
| Wu 2007 | 848 | Wu Y-L, Zhong W-Z, Li L-Y, Zhang X-T, Zhang L, Zhou C-C, Liu W, Jiang B, Mu X-L, Lin J-Y, Zhou Q, Xu C-R, Wang Z, Zhang G-C & Mok T (2007): Epidermal growth factor receptor mutations and their correlation with gefitinib therapy in patients with non-small cell lung cancer: a meta-analysis based on updated individual patient data from six medical centers in mainland China. *Journal of Thoracic Oncology: Official Publication of the International Association for the Study of Lung Cancer* **2**, 430-439. |
| Wu 2012 | 390 | Wu Y, Liu H-B, Ding M, Liu J-N, Zhan P, Fu X-S & Lu G (2012b): The impact of E-cadherin expression on non-small cell lung cancer survival: a meta-analysis. *Molecular Biology Reports* **39**, 9621-9628. |
| Xia 2014 | 78 | Xia L, Ren Y, Fang X, Yin Z, Li X, Wu W, Guan P & Zhou B (2014): Prognostic role of common microRNA polymorphisms in cancers: evidence from a meta-analysis. *PLoS ONE [Electronic Resource]* **9**, e106799. |
| Xie 2016 | 5563 | Xie F-J, Lu H-Y, Zheng Q-Q, Qin J, Gao Y, Zhang Y-P, Hu X & Mao W-M (2016): The clinical pathological characteristics and prognosis of FGFR1 gene amplification in non-small-cell lung cancer: a meta-analysis. *OncoTargets and Therapy* **9**. |
| Xie, 2012 | 368 | Xie Y-L, An L, Jiang H & Wang J (2012): Nuclear survivin expression is associated with a poor prognosis in Caucasian non-small cell lung cancer patients. *Clinica Chimica Acta* **414**, 41-43. |
| Xing 2013 | 5058 | Xing XB, Cai WB, Luo L, Liu LS, Shi HJ & Chen MH (2013): The Prognostic Value of p16 Hypermethylation in Cancer: A Meta-Analysis. *PLoS ONE [Electronic Resource]* **8**. |
| Xu, 2015 | 5614 | Xu JL, Jin B, Ren ZH, Lou YQ, Zhou ZR, Yang QZ & Han BH (2015a): Chemotherapy plus erlotinib versus chemotherapy alone for treating advanced non-small cell lung cancer: A meta-analysis. *PLoS ONE [Electronic Resource]* **10**. |
| Xu, 2015 | 5695 | Xu L, Lan H, Su Y, Li J & Wan J (2015b): Clinicopathological significance and potential drug target of RUNX3 in non-small cell lung cancer: A meta-analysis. *Drug Design, Development and Therapy* **9**, 2855-2865. |
| Xu, 2013 | 269 | Xu T-P, Shen H, Liu L-X & Shu Y-Q (2013a): Association of ERCC1-C118T and -C8092A polymorphisms with lung cancer risk and survival of advanced-stage non-small cell lung cancer patients receiving platinum-based chemotherapy: a pooled analysis based on 39 reports. *Gene* **526**, 265-274. |
| Xu, 2013 | 148 | Xu T-P, Zhu C-H, Zhang J, Xia R, Wu F-L, Han L, Shen H, Liu L-X & Shu Y-Q (2013b): MicroRNA-155 expression has prognostic value in patients with non-small cell lung cancer and digestive system carcinomas. *Asian Pacific Journal of Cancer Prevention: Apjcp* **14**, 7085-7090. |
| Xu, 2014 | 199 | Xu YH & Lu S (2014): A meta-analysis of STAT3 and phospho-STAT3 expression and survival of patients with non-small-cell lung cancer. *European Journal of Surgical Oncology* **40**, 311-317. |
| Xue, 2014 | 5037 | Xue RL, Yang CL, Zhao F & Li DJ (2014): Prognostic significance of CDH13 hypermethylation and mRNA in NSCLC. *OncoTargets and Therapy* **7**, 1987-1996. |
| Yan, 2014 | 5962 | Yan B, Zhang W, Jiang LY, Qin WX & Wang X (2014a): Reduced E-cadherin expression is a prognostic biomarker of non-small cell lung cancer: A meta-analysis based on 2395 subjects. *International Journal of Clinical and Experimental Medicine* **7**, 4352-4356. |
| Yan, 2014 | 192 | Yan H-J, Tan Y & Gu W (2014b): Neuron specific enolase and prognosis of non-small cell lung cancer: a systematic review and meta-analysis. *Journal of B U On* **19**, 153-156. |
| Yang 2013 | 338 | Yang M, Shen H, Qiu C, Ni Y, Wang L, Dong W, Liao Y & Du J (2013a): High expression of miR-21 and miR-155 predicts recurrence and unfavourable survival in non-small cell lung cancer. *European Journal of Cancer* **49**, 604-615. |
| Yang, 2014 | 6065 | Yang W, Yao YW, Zeng JL, Liang WJ, Wang L, Bai CQ, Liu CH & Song Y (2014a): Prognostic value of FGFR1 gene copy number in patients with non-small cell lung cancer: A meta-analysis. *Journal of Thoracic Disease* **6**, 803-809. |
| Yang, 2014 | 19 | Yang Y-L, Chen M-W & Xian L (2014b): Prognostic and clinicopathological significance of downregulated E-cadherin expression in patients with non-small cell lung cancer (NSCLC): a meta-analysis. *PLoS ONE [Electronic Resource]* **9**, e99763. |
| Yang, 2014 | 165 | Yang Y-L, Luo X-P & Xian L (2014c): The prognostic role of the class III beta-tubulin in non-small cell lung cancer (NSCLC) patients receiving the taxane/vinorebine-based chemotherapy: a meta-analysis. *PLoS ONE [Electronic Resource]* **9**, e93997. |
| Yang, 2014 | 169 | Yang Y, Luo J, Zhai X, Fu Z, Tang Z, Liu L, Chen M & Zhu Y (2014d): Prognostic value of phospho-Akt in patients with non-small cell lung carcinoma: a meta-analysis. *International Journal of Cancer* **135**, 1417-1424. |
| Yang, 2014 | 68 | Yang Y, Luo X, Yang N, Feng R & Xian L (2014e): The prognostic value of excision repair cross-complementation group 1 (ERCC1) in patients with small cell lung cancer (SCLC) receiving platinum-based chemotherapy: evidence from meta-analysis. *PLoS ONE [Electronic Resource]* **9**, e111651. |
| Yang, 2014 | 184 | Yang Y & Xian L (2014): The association between the ERCC1/2 polymorphisms and the clinical outcomes of the platinum-based chemotherapy in non-small cell lung cancer (NSCLC): a systematic review and meta-analysis. *Tumour Biology* **35**, 2905-2921. |
| Yang, 2013 | 318 | Yang Y, Xie Y & Xian L (2013b): Breast cancer susceptibility gene 1 (BRCA1) predict clinical outcome in platinum- and toxal-based chemotherapy in non-small-cell lung cancer (NSCLC) patients: a system review and meta-analysis. *Journal of Experimental & Clinical Cancer Research* **32**, 15. |
| Yin, 2011 | 526 | Yin M, Yan J, Voutsina A, Tibaldi C, Christiani DC, Heist RS, Rosell R, Booton R & Wei Q (2011): No evidence of an association of ERCC1 and ERCC2 polymorphisms with clinical outcomes of platinum-based chemotherapies in non-small cell lung cancer: a meta-analysis. *Lung Cancer* **72**, 370-377. |
| Ying, 2015 | 57 | Ying M, Zhu X, Chen K, Sha Z & Chen L (2015): Should KRAS mutation still be used as a routine predictor of response to EGFR-TKIs in advanced non-small-cell lung cancer? A revaluation based on meta-analysis. *Journal of Cancer Research & Clinical Oncology* **141**, 1427-1439. |
| Yu, 2015 | 5489 | Yu Y, Guan H, Xing LG & Xiang YB (2015): Role of Gross Tumor Volume in the Prognosis of Non-small Cell Lung Cancer Treated with 3D Conformal Radiotherapy: A Meta-analysis. *Clinical Therapeutics* **37**, 2256-2266. |
| Zeng, 2015 | 5640 | Zeng C, Fan W & Zhang X (2015a): RRM1 expression is associated with the outcome of gemcitabine-based treatment of non-small cell lung cancer patients-a short report. *Cellular Oncology* **38**, 319-325. |
| Zeng, 2015 | 5601 | Zeng Y, Zhang Q, Wang H, Lu M, Kong H, Zhang Y & Shi H (2015b): Prognostic significance of interleukin-17 in solid tumors: A meta-analysis. *International Journal of Clinical and Experimental Medicine* **8**, 10515-10536. |
| Zhan, 2009 | 5067 | Zhan P, Qian Q & Yu LK (2013): Prognostic value of COX-2 expression in patients with non-small cell lung cancer: a systematic review and meta-analysis. *Journal of Thoracic Disease* **5**, 40-47. |
| Zhan, 2013 | 724 | Zhan P, Wang J, Lv X-j, Wang Q, Qiu L-x, Lin X-q, Yu L-k & Song Y (2009): Prognostic value of vascular endothelial growth factor expression in patients with lung cancer: a systematic review with meta-analysis. *Journal of Thoracic Oncology: Official Publication of the International Association for the Study of Lung Cancer* **4**, 1094-1103. |
| Zhang, 2015 | 5693 | Zhang C, Li J, Han Y & Jiang J (2015a): Ameta-analysis for CXCRCR4 as a prognostic marker and potential drug target in non-small cell lung cancer. *Drug Design, Development and Therapy* **9**, 3267-3278. |
| Zhang, 2012 | 420 | Zhang H-L, Ruan L, Zheng L-M, Whyte D, Tzeng C-M & Zhou X-W (2012a): Association between class III beta-tubulin expression and response to paclitaxel/vinorebine-based chemotherapy for non-small cell lung cancer: a meta-analysis. *Lung Cancer* **77**, 9-15. |
| Zhang, 2014 | 81 | Zhang J, Wang H-T & Li B-G (2014a): Prognostic significance of circulating tumor cells in small--cell lung cancer patients: a meta-analysis. *Asian Pacific Journal of Cancer Prevention: Apjcp* **15**, 8429-8433. |
| Zhang, 2015 | 5572 | Zhang J, Yu XL, Zheng GF & Zhao F (2015b): DAPK promoter methylation status correlates with tumor metastasis and poor prognosis in patients with non-small cell lung cancer. *Cancer Biomarkers* **15**, 609-617. |
| Zhang, 2015 | 5707 | Zhang J, Zhang B, Wang T & Wang H (2015c): LncRNA MALAT1 overexpression is an unfavorable prognostic factor in human cancer: Evidence from a meta-analysis. *International Journal of Clinical and Experimental Medicine* **8**, 5499-5505. |
| Zhang, 2012 | 442 | Zhang LQ, Jiang F, Xu L, Wang J, Bai JL, Yin R, Wu YQ & Meng LJ (2012b): The role of cyclin D1 expression and patient's survival in non-small-cell lung cancer: a systematic review with meta-analysis. *Clinical Lung Cancer* **13**, 188-195. |
| Zhang, 2012 | 446 | Zhang LQ, Wang J, Jiang F, Xu L, Liu FY & Yin R (2012c): Prognostic value of survivin in patients with non-small cell lung carcinoma: a systematic review with meta-analysis. *PLoS ONE [Electronic Resource]* **7**, e34100. |
| Zhang, 2014 | 113 | Zhang Q, Dai H-H, Dong H-Y, Sun C-T, Yang Z & Han J-Q (2014b): EGFR mutations and clinical outcomes of chemotherapy for advanced non-small cell lung cancer: a meta-analysis. *Lung Cancer* **85**, 339-345. |
| Zhang, 2014 | 47 | Zhang W-Q, Li T & Li H (2014c): Efficacy of EGFR tyrosine kinase inhibitors in non-small-cell lung cancer patients with/without EGFR-mutation: evidence based on recent phase III randomized trials. *Medical Science Monitor* **20**, 2666-2676. |
| Zhang, 2014 | 5035 | Zhang X, Weng WH, Xu W, Wang YL, Yu WJ, Tang X, Ma LF, Pan QH, Wang JY & Sun FY (2014d): Prognostic significance of interleukin 17 in cancer: a meta-analysis. *International Journal of Clinical and Experimental Medicine* **7**, 3258-3269. |
| Zhang, 2014 | 11 | Zhang Z, Wang T, Zhang J, Cai X, Pan C, Long Y, Chen J, Zhou C & Yin X (2014e): Prognostic value of epidermal growth factor receptor mutations in resected non-small cell lung cancer: a systematic review with meta-analysis. *PLoS ONE [Electronic Resource]* **9**, e106053. |
| Zhao 2015 | 553 | Zhao QT, Yang Y, Xu S, Zhang XP, Wang HE, Zhang H, Wang ZK, Yuan Z & Duan GC (2015): Prognostic role of neutrophil to lymphocyte ratio in lung cancers: A meta-analysis including 7,054 patients. *OncoTargets and Therapy* **8**, 2731-2738. |
| Zhao, 2014 | 98 | Zhao S, He J-L, Qiu Z-X, Chen N-Y, Luo Z, Chen B-J & Li W-M (2014a): Prognostic value of CD44 variant exon 6 expression in non-small cell lung cancer: a meta-analysis. *Asian Pacific Journal of Cancer Prevention: Apjcp* **15**, 6761-6766. |
| Zhao, 2014 | 69 | Zhao S, Qiu Z, He J, Li L & Li W (2014b): Insulin-like growth factor receptor 1 (IGF1R) expression and survival in non-small cell lung cancer patients: a meta-analysis. *International Journal of Clinical & Experimental Pathology* **7**, 6694-6704. |
| Zhao, 2013 | 243 | Zhao W-X & Luo J-f (2013): Serum neuron-specific enolase levels were associated with the prognosis of small cell lung cancer: a meta-analysis. *Tumour Biology* **34**, 3245-3248. |
| Zhao, 2014 | 74 | Zhao X-D, He Y-Y, Gao J, Zhao C, Zhang L-L, Tian J-Y & Chen H-L (2014c): High expression of Bcl-2 protein predicts favorable outcome in non-small cell lung cancer: evidence from a systematic review and meta-analysis. *Asian Pacific Journal of Cancer Prevention: Apjcp* **15**, 8861-8869. |
| Zhou 2014 | 33 | Zhou X, Du Y, Huang Z, Xu J, Qiu T, Wang J, Wang T, Zhu W & Liu P (2014): Prognostic value of PLR in various cancers: a meta-analysis. *PLoS ONE [Electronic Resource]* **9**, e101119. |
| Zhou, 2015 | 5779 | Zhou XM, He L, Hou G, Jiang B, Wang YH & Zhao L (2015): Clinicopathological significance of CXCR4 in non-small cell lung cancer. *Drug Design, Development and Therapy* **9**, 1349-1358. |
| Zhu, 2015 | 5610 | Zhu L, Liu J, Ma S & Zhang S (2015a): Long Noncoding RNA MALAT-1 Can Predict Metastasis and a Poor Prognosis: a Meta-Analysis. *Pathology and Oncology Research* **21**, 1259-1264. |
| Zhu, 2015 | 4969 | Zhu L, Yu H, Liu SY, Xiao XS, Dong WH, Chen YN, Xu W & Zhu T (2015b): Prognostic Value of Tissue Inhibitor of Metalloproteinase-2 Expression in Patients with Non-Small Cell Lung Cancer: A Systematic Review and Meta-Analysis. *PLoS ONE [Electronic Resource]* **10**. |
| Zhu, 2014 | 120 | Zhu W & Xu B (2014): MicroRNA-21 identified as predictor of cancer outcome: a meta-analysis. *PLoS ONE [Electronic Resource]* **9**, e103373. |
| Zhuang, 2011 | 489 | Zhuang Y, Yin H-T, Yin X-L, Wang J & Zhang D-P (2011): High p27 expression is associated with a better prognosis in East Asian non-small cell lung cancer patients. *Clinica Chimica Acta* **412**, 2228-2231. |
| Zou, 2015 | 5829 | Zou XL, Wang C, Liu K, Nie W & Ding ZY (2015): Prognostic significance of osteopontin expression in non-small-cell lung cancer: A meta-analysis. *Molecular and Clinical Oncology* **3**, 633-638. |
