# Supplementary material for: Pragmatic methods for reviewing exceptionally large bodies of evidence: systematic mapping review and overview of systematic reviews using lung cancer survival as an exemplar
Source: Syst Rev. 2019 Jul 16;8:171. doi: 10.1186/s13643-019-1087-4 (PMC6631880; doi:10.1186/s13643-019-1087-4)
Supplement: Supplementary file 4 — Appendix D. Results of reviews in stage 2, overview of reviews. Table D1. ‘New’ biomarkers or biological factors not used in routine practice. Table D2. Tumour characteristics. Table D3. Metabolic criteria. Table D4. Clinical characteristics or routinely assessed biological variables. Table D5. Patient characteristics. Table D6. Healthcare provider and system. Table D7. Prognostic factors classified as ‘other’. (DOCX 96 kb) [file 13643_2019_1087_MOESM4_ESM.docx]

**APPENDIX D: RESULTS OF REVIEWS IN STAGE 2, OVERVIEW OF REVIEWS**

**Table D1: ‘New’ biomarkers or biological factors not used in routine practice**

| **Author, year** | **REV ID** | **LC Type** | **LC Subgroup** | **PF Cat** | **PF** | **PF description** | **Measure** | **Study design** | **evaluable studies** | **Sample size range** | **Summary Measure** | **Pooled results** | **Narrative synthesis** |
| --- | --- | --- | --- | --- | --- | --- | --- | --- | --- | --- | --- | --- | --- |
| Huo, 2015 | 5784 | LC |  | B | ALDH1 | Positive ALDH1 expression | Binary | NS | 5 | 50-296 | RR | **-** | <> in 4 studies (NSCLC);  + in 1 study (LC) |
| Wei 2015 | 4957 | LC | None | B | ALDH1 | Aldehyde dehydrogenase 1 expression (vs non expression) | Binary | NS | 6 | 52-282 | RR | **-** | - in six studies |
| Choma, 2001 | 1085 | NSCLC | surR | B | Aneuploidy | Presence of aneuploid DNA content | Binary | Mixed |  | 44-340 | OR | **-** |  |
| Martin, 2003 | 1024 | LC | (NSCLC) | B | Bcl-2 | Bcl-2 positive expression in NSCLC | Binary | Retrospective | 18 | 27-485 | HR | **+** |  |
| Martin, 2003 | 1024 | LC | (SCLC) | B | Bcl-2 | Bcl-2 positive expression in SCLC | Binary | Retrospective | 5 | 38-146 | HR | **+** |  |
| Martin, 2003 | 1024 | LC | (NLHC) | B | Bcl-2 | Bcl-2 positive expression in NLHC | Binary | Retrospective | 3 | 23-43 | HR | **<>** |  |
| Zhao, 2014 | 74 | NSCLC | IIIB-IV | B | Bcl-2 | High/ over (vs low/ no) expression of Bcl-2 protein | Binary | NS | 54 | 45-535 | HR | **+** |  |
| Mei, 2013 | 319 | NSCLC |  | B | Beta-catenin | Reduced Beta-catenin expression | Binary | NS | 12 | 35-522 | HR | **-** |  |
| Ellis, 2010 | 7352 | NSCLC | none | B | bFGF | High levels of bFGF | Binary | Retrospective | 1 | 358 | NS |  | - 1 study |
| Chen, 2014 | 86 | NSCLC |  | B | Cav-1 | Positive Cav-1 expression | Binary | Mixture | 8 | 43-160 | HR | **-** |  |
| Qu, 2013 | 233 | NSCLC | IIIB-IV | B | CD133 | Positive CD133 expression | Binary | NS | 5 | 50-161 | RR | **-** | - in 5 studies |
| Wu 2014 | 30 | NSCLC | None | B | CD133 | Positive CD133 expression | Binary | NS | 14 | 55-305 | OR | **-** | - in 11 studies |
| Jiang, 2014 | 140 | NSCLC |  | B | CD44 | High expression of CD44 or CD44 isoforms (CD44) | Binary | Retrospective | 3 | 29-123 | HR | **<>** |  |
| Jiang, 2014 | 140 | NSCLC |  | B | CD44 | High expression of CD44 standard form, CD44s | Binary | Retrospective | 2 | 77-164 | HR | **-** |  |
| Jiang, 2014 | 140 | NSCLC |  | B | CD44 | High expression of CD44 variant 6, CD44v6 | Binary | Retrospective | 5 | 34-190 | HR | **-** |  |
| Zhao 2014 | 98 | NSCLC | met | B | CD44 | CD44 variant exon expression | Binary | NS | 10 | 34-190 | HR | - | - in 10 studies |
| Xue, 2014 | 5037 | NSCLC | None | B | CDH13 / CDH13 microRNA | CDH13 hypermethylation | Binary | NS | 4 | 78-150 | HR | **-** |  |
| Wang, 2014 | 150 | NSCLC | <IV | B | CEA | Preoperative serum CEA level over expression | Binary | Retrospective | 16 | 43-1000 | HR | - | - in 16 studies |
| Jiang, 2013 | 312 | NSCLC |  | B | COX-2 | High COX-2 expression | Binary | NS | 19 | 43-259 | HR | **-** | - in 12 studies; + in 1 study; <> in 6 studies |
| Mascaux, 2006 | 899 | LC |  | B | COX-2 | COX-2 overexpression [*10/14 eligible studies included in MA]* | Binary | NS | 10 | 60-259 | HR | **<>** | - in 6 studies (1 LC; 1 ADC; 4 NSCLC) [5 evaluable]; <> in 7 studies (2 ADC; 5 NSCLC) [4 evaluable]; + in 1 study (1 ADC) [evaluable] |
| Zhan, 2013 | 5067 | NSCLC | None | B | COX-2 | cyclooxygenase-2 (COX-2) overexpression | Binary | NS | 16 | 60-259 | HR | **<>** |  |
| Jin, 2014 | 67 | NSCLC | None | B | CRP | Elevated Circulating C-reactive protein levels | Binary | NS | 8 | NS | HR | **-** |  |
| Jing, 2015 | 5605 | NSCLC | SurR | B | CRP | CRP - elevated serum level (preoperative) | Binary | Mixed | 5 | 105-301 | RR | **-** |  |
| Liu 2014 | 76 | NSCLC | None | B | CRP | C-reactive protein | Binary | Mixed | 23 | 36-334 | HR | - |  |
| Liang, 2015 | 5708 | LC; NSCLC: SCLC |  | B | CXCR4 | Chemokine receptor (G protein-coupled receptor) CXCR4 expression (vs no expression) | Binary | NS | 8 | 16-208 | HR | **-** |  |
| Liu, 2015 | 5677 | NSCLC | I-II; III; IV | B | CXCR4 | High(over) (vs low) C-X-C Chemokine receptor type 4 (CXCR4) expression | Binary | NS | 9 | 45-323 | HR | **-** |  |
| Zhang, 2015 | 5693 | NSCLC | None | B | CXCR4 | High (vs low) CXCR4 protein expression | Binary | NS | 5 | 125-236 | HR | **-** |  |
| Zhou 2015 | 5779 | NSCLC | None | B | CXCR4 | CXCR4 expression - positive or present | Binary | NS | 4 | 110-236 | HR | **-** |  |
| Zhang, 2012 | 442 | NSCLC | IIIB-IV | B | cyclin D1 | Cyclin D1 expression (vs no expression) | Binary | NS | 24 | 43-390 | HR | **<>** |  |
| Huang, 2102 | 454 | LC |  | B | cyclin E | Positive expression [*Included 14 studies: 2 LC, 12 NSCLC]* | Binary | Retrospective | 14 | 62-778 | HR | **-** |  |
| Huang, 2102 | 454 | LC | (NSCLC) | B | cyclin E | Positive expression | Binary | Retrospective | 12 | 62-778 | HR | **-** |  |
| Pujol, 2004 | 993 | NSCLC | None | B | CYFRA 21-1 | Serum CYFRA 21-1 level at presentation in NSCLC – high vs normal | Binary | Mixed | NS (2063 pts) | 2063 | HR | **-** |  |
| Zhang, 2015 | 5572 | NSCLC | <IV | B | DAPK | Present (vs absent) Promoter methylation of death-associated protein kinase (DAPK) | Binary | NS | 4 | 28-200 | RR | **-** |  |
| Qiu, 2014 | 5592 | NSCLC | None | B | E-cadherin | E-cadherin over expression | Binary | NS | 15 | 50-76 | HR | **+** | - in 7 studies;  <> in 1 study,  + in 7 studies |
| Wu 2012 | 390 | NSCLC | None | B | E-cadherin | Reduced or absent E-Cadherin expression | Binary | Mixed | 13 | 73-391 | HR | **<>** |  |
| Yan, 2014 | 5962 | NSCLC | None | B | E-cadherin | Reduced/low E-Cadherin expression | Binary | NS | 11 | NS | HR | **-** |  |
| Yang, 2014 | 19 | NSCLC | None | B | E-cadherin | reduced E-cadherin expression | Binary | Prospective | 22 | 48-405 | HR | **-** |  |
| Carlson 2009 | 720 | NSCLC | WT | B | EGFR | EGFR mutation | Binary | Mixed | NS | NS | HR | **+** |  |
| Carlson 2009 | 720 | NSCLC | WT | B | EGFR | High protein expression | Binary | Mixed | NS | NS | HR | **+** |  |
| Carlson 2009 | 720 | NSCLC | WT | B | EGFR | High EGFR expression | Binary | Mixed | NS | NS | NS | **<>** |  |
| Ellis, 2010 | 7352 | NSCLC | none | B | EGFR | Positive EGFR gene copy identified by FISH | Binary | Retrospective | 1 | 159 | NS |  | - 1 study |
| Ellis, 2010 | 7352 | NSCLC | none | B | EGFR | Presence of EGFR mutations | Binary | Retrospective | 3 | 204-297 | NS |  | + 2 studies  <> 1 study |
| Meert, 2002 | 1045 | LC | (NSCLC) | B | EGFR | EGF-R overexpression [*14 NSCLC, 1 ADC, 1 SCC*] | Binary | NS | 11 | 19-505 | HR | **<>** | - in 3 studies (1 SCC; 3 evaluable); <> in 12 studies (1 ADC; 7 evaluable); + in 1 study (evaluable). |
| Nakamura, 2006 | 918 | NSCLC | IIIB-IV | B | EGFR | Epidermal growth factor receptor (EGFR) overexpression | Binary | NS | 18 | 19-515 | HR | **<>** |  |
| Quinton, 2011 | 6781 | NSCLC | IIIB-IV | B | EGFR | EGFR gene mutation (vs no mutation) | Binary | Prospective | 14 | NS (total 3259) | HR |  | + in 14 studies |
| Quinton, 2011 | 6781 | NSCLC | IIIB-IV | B | EGFR | EGFR protein expression (vs no expression) | Binary | Prospective | 6 | NS (total 2691) | HR |  | + in 2 studies <> in 4 studies |
| Wu 2007 | 848 | Early NSCLC | EGFR | B | EGFR | Epidermal growth factor receptor mutations | Binary | Retrospective | 2 | 76-142 | HR | **<>** |  |
| Xu, 2015 | 5614 | NSCLC | late | B | EGFR | Presence of EGFR mutant tumours | Binary | Prospective | 2 | 451-1079 | HR | **+** |  |
| Xu, 2015 | 5614 | NSCLC | late | B | EGFR | presence EGFR wild-type tumours present | Binary | Prospective | 2 | 451-1079 | HR | **<>** |  |
| Zhang, 2014 | 11 | NSCLC | <IV | B | EGFR | Epidermal Growth Factor Receptor Mutation status (present or not) | Binary | Mixed | 16 | 53-307 | HR | **<>** |  |
| Zhang, 2014 | 47 | NSCLC | IIIB-IV | B | EGFR | EGFR-Mutation present (vs absent) | Binary | Prospective | 17 | 135-1466 | HR | **<>** |  |
| Zhang, 2014 | 113 | NSCLC | IIIB-IV | B | EGFR | EGFR-Mutation present (vs absent) | Binary | Mixed | 9 | 54-353 | HR | **<>** |  |
| Quinton, 2011 | 6781 | NSCLC | IIIB-IV | B | EGFR subtypes | EGFR copy number identified via FISH (vs qPCR) | Binary | Prospective | 9 | NS (total 2994) | HR |  | + in 2 studies - in 7 studies |
| Wang, 2014 | 129 | NSCLC | IIIB-IV | B | EGFR subtypes | Exon 19 (19del) mutation vs position 858 (21L858R) mutation | Binary | Mixed | 3 | 17-57 | RR | **+** |  |
| Shen, 2014 | 62 | mxdC NSCLC | None | B | EphA2 | EphA2 overexpression (vs low or no expression) | Binary | NS | 4 | 94-279 | HR | **<>** |  |
| Luo, 2015 | 5651 | NSCLC | None | B | ERbeta | Over-expression of Erβ in tumour tissues | Binary | NS | 14 | 58 - 301 | HR | **+ in UVA;**  **<> in MVA** |  |
| Breen, 2008 | 791 | NSCLC | early | B | ERCC1 | ERCC1 positive or high level | Binary | NS | 7 | 44-760 | HR |  | + in 4 studies; <> in 2 studies; <>/+ in 1 study (<> for surgery+CTX, + surgery alone) |
| Chen, 2010 | 635 | NSCLC | late | B | ERCC1 | High/positive ERCC1 expression (CTX treated patients) | Binary | NS | 11 | 45-200 | Median survival | **-** |  |
| Ellis, 2010 | 7352 | NSCLC | none | B | ERCC1 | ERCC1 IHC expression | Binary | Retrospective | 1 | 761 | NS |  | + 1 study |
| Ellis, 2010 | 7352 | NSCLC | none | B | ERCC1 | ERCC1 mRNA high | Binary | Retrospective | 1 | 66 | NS |  | <> 1 study |
| Hubner, 2011 | 483 | LC | NSCLC (no CTX) | B | ERCC1 | Higher ERCC1 expression in NSCLC without systemic therapy | Binary | Retrospective | 6 | NS | HR | **<>** |  |
| Hubner, 2011 | 483 | LC | NSCLC (CTX) | B | ERCC1 | Higher ERCC1 expression in NSCLC with platinum-based treatments | Binary | Retrospective | 13 | NS | HR | **-** |  |
| Hubner, 2011 | 483 | LC | SCLC | B | ERCC1 | Higher ERCC1 expression in SCLC | Binary | Retrospective | 5 | NS | HR | **<>** |  |
| Jiang, 2012 | 424 | NSCLC | (early) | B | ERCC1 | High ERCC1 expression (in surgery alone group; early stage cancer) | Binary | NS | 6 | 51-372 | HR | **+** |  |
| Jiang, 2012 | 424 | NSCLC | (early) | B | ERCC1 | High ERCC1 expression in surgery plus adjuvant CTX treated pts | Binary | NS | 7 | 15-389 | HR | **<>** |  |
| Jiang, 2012 | 424 | NSCLC | (late) | B | ERCC1 | High ERCC1 expression (in palliative CTX; advanced cancer) | Binary | NS | 16 | 21-264 | HR | **-** |  |
| Knez, 2011 | 525 | SCLC |  | B | ERCC1 | High protein expression (PE) or gene expression (GE) of ERCC1 (CTX treated patients) | Binary | Retrospective | 6 | 77-186 | p-value/ narrative |  | - in 1 study (UVA only); <> 5 studies |
| Roth, 2011 | 477 | NSCLC | IIIB-IV | B | ERCC1 | High (vs low) ERCC1 expression level (CTX treated patients) | Binary | Mixed | 8 | 40-163 | HR | **-** |  |
| Yang, 2014 | 68 | SCLC | None | B | ERCC1 | Positive/ high (vs no/low) expression of Excision Repair Cross-Complementation Group 1 (ERCC1) (CTX treated patients) | Binary | NS | 8 | 64-323 | HR | **-** |  |
| Knez, 2011 | 525 | SCLC |  | B | ERCC1 polymorphism | ERCC1 gene polymorphisms or SNPs | Binary | NS | 1 | NS | p-value |  | 262 GG genotype: + 1 study |
| Xu, 2013 | 269 | NSCLC | late | B | ERCC1 polymorphs | ERCC1 C118T [CT or TT genotype] | Binary | NS | 18 | 54-632 | HR | **-** |  |
| Xu, 2013 | 269 | NSCLC | late | B | ERCC1 polymorphs | ERCC1 C8092A polymorphs | Binary | NS | 6 | 90-300 | HR | **<>** |  |
| Yang, 2014 | 184 | NSCLC | None | B | ERCC1/2 polymorphs | ERCC1 C118T [CT or TT] | Categorical | NS | 10 | 62-381 | HR | **-** |  |
| Yang, 2014 | 184 | NSCLC | None | B | ERCC1/2 polymorphs | ERCC1 C8092A [CA or AA] | Categorical | NS | 4 | 119-493 | HR | **-** |  |
| Yang, 2014 | 184 | NSCLC | None | B | ERCC1/2 polymorphs | Presence of the allele ERCC2 Asp312Asn | Categorical | NS | 8 | 62-493 | HR | **-** |  |
| Yang, 2014 | 184 | NSCLC | None | B | ERCC1/2 polymorphs | Presence of the Gln allele ERCC2 Lys751Gln | Binary | NS | 11 | 62-493 | HR | **-** |  |
| Yin, 2011 | 526 | NSCLC | None | B | ERCC1/2 polymorphs | ERCC1 C118T [T/T or C/T] | Categorical | NS | 8 | 62-245 | HR | **<>** |  |
| Yin, 2011 | 526 | NSCLC | None | B | ERCC1/2 polymorphs | ERCC1 C8092A [AA or AC] | Categorical | NS | 4 | NS (total 582) | NS | **<>** |  |
| Yin, 2011 | 526 | NSCLC | None | B | ERCC1/2 polymorphs | ERCC2 Asp312Asn | Categorical | NS | 6 | NS (total 589) | NS | **<>** |  |
| Yin, 2011 | 526 | NSCLC | None | B | ERCC1/2 polymorphs | ERCC2 Lys751Gln | Categorical | NS | 7 | 62-381 | HR | **<>** |  |
| Li, 2015 | 5436 | NSCLC |  | B | ESR1 | Positive ESR1 mRNA expression in univariate analysis | Binary | Mixed | 6 | 31-178 | HR | **+** |  |
| Li, 2015 | 5436 | NSCLC |  | B | ESR1 | Positive ESR1 mRNA expression in MVA | Binary | Mixed | 3 | 31-90 | HR | **+** |  |
| Li, 2015 | 5436 | NSCLC |  | B | ESR2 mRNA | Positive ESR2 mRNA expression | Binary | Mixed | 4 | 66-178 | HR | **<>** |  |
| Chen 2015 | 5751 | mxdC NSCLC | None | B | EZH2 | Enhancer of Zeste Homolog 2 high expression | Binary | NS | 3 | 580 | HR | **-** | - in 3 studies |
| Jiang, 2015 | 4988 | LSCC |  | B | FGFR1 | FGFRI amplification | Binary | NS | 5 | 59-345 | HR | **<>** |  |
| Xie, 2016 | 5563 | NSCLC | None | B | FGFR1 | FGFR1 high amplification (vs not high amplification) | Binary | NS | 13 | 100-628 | HR | **-** |  |
| Yang, 2014 | 6065 | NSCLC | None | B | FGFR1 | FGFR1 amplification (vs non-amplification) | Binary | NS | 6 | 100-264 | HR | **<>** |  |
| Dai, 2015 | 5508 | mxdC NSCLC |  | B | FOXM1 | FOXM1 expression | Binary | NS | 7 | 68-201 | HR | **-** |  |
| Liu, 2010 | 612 | LC |  | B | HER-2 | Human Epidermal Growth Factor Receptor 2 (HER2) overexpression | Binary | NS | 37 | 42-515 | HR | **-** |  |
| Liu, 2010 | 612 | LC |  | B | HER-2 | Human Epidermal Growth Factor Receptor 2 (HER2) overexpression | Binary | NS | 3 | 67-193 | HR | **-** |  |
| Liu, 2010 | 612 | LC |  | B | HER-2 | Human Epidermal Growth Factor Receptor 2 (HER2) overexpression | Binary | NS | 40 | 42-515 | HR |  | - in 13 studies <> in 27 studies |
| Meert, 2003 | 1019 | LC | (NSCLC) | B | HER-2 | C-erbB-2 overexpression [HER-2neu expression] in NSCLC | Binary | NS | 20 | 43-483 | HR | **-** | - in 12 studies (11 evaluable); <> in 16 studies (9 evaluable); + in 1 study (not evaluable). |
| Meert, 2003 | 1019 | LC | (SCLC, ext) | B | HER-2 | c-erbB-2 overexpression [HER-2neu expression] in SCLC | Binary | NS | 1 | 193 | Survival curves |  | - In 1 study |
| Nakamura, 2005 | 952 | NSCLC |  | B | HER-2 | HER-2 overexpression | Binary | NS | 20 | 42-408 | HR | **-** |  |
| Li, 2013 | 272 | NSCLC |  | B | HIF-1alpha | High HIF1-alpha expression | Binary | NS | 7 | 75-178 | HR | **-** |  |
| Ren, 2013 | 249 | LC |  | B | HIF-1alpha | HIF-1alpha over-expression | Binary | NS | **7** | **80 - 328** | OR | **-** |  |
| Ren, 2013 | 249 | LC (SCC, ADC & LCC) |  | B | HIF-1alpha | HIF-1alpha expression in SCC, ADC & LCC | Binary | NS | 1 | 328 | OR |  | - in 1 study |
| Ren, 2013 | 249 | LC (SCC & ADC) |  | B | HIF-1alpha | HIF-1alpha expression in SCC & ADC | Binary | NS | 1 | 170 | OR |  | - in 1 study |
| Ren, 2013 | 249 | LC (SCC, ADC & SCLC) |  | B | HIF-1alpha | HIF-1alpha expression in SCC, ADC & SCLC | Binary | NS | 1 | 75 | OR |  | - in 1 study |
| Ren, 2013 | 249 | LC (NSCLC) |  | B | HIF-1alpha | HIF-1alpha expression in NSCLC | Binary | NS | 1 | 60 | OR |  | <> in 1 study |
| Wang, 2014 | 187 | LC | <IV | B | HIF-1alpha | High (vs low or no) Hypoxia-inducible factor 1@ (HIF-1@) expression | Binary | NS | 13 | 45-178 | HR | **-** |  |
| Li, 2013 | 272 | NSCLC |  | B | HIF-2alpha | High HIF-2 alpha expression | Binary | NS | 3 | 51-140 | HR | **-** |  |
| Ellis, 2010 | 7352 | NSCLC | none | B | ICAM | Low levels of ICAM | Binary | Retrospective | 1 | 160 | NS |  | + 1 study |
| Ellis, 2010 | 7352 | NSCLC | none | B | ICAM-1 | High levels of ICAM-1 | Binary | Retrospective | 1 | 358 | NS |  | - 1 study |
| Zhao, 2014 | 69 | NSCLC | IIIB-IV | B | IGF1R | Positive/ over (vs low/ no) expression of Insulin-like growth factor receptor-1 (IGF1R) | Binary | Mixed | 12 | 39-459 | HR | **-** |  |
| Zeng, 2015 | 5601 | NSCLC | None | C/B | IL-17 | High interleukin-17 (IL17) expression | Binary | NS | 2 | 52-101 | HR | **-** |  |
| Zhang, 2014 | 5035 | mxdC NSCLC | IIIB-IV | C/B | IL-17 | Interleukin 17 expression (vs no expression) | Binary | NS | 18 | 32-300 | HR | **<>** |  |
| Liu 2014 | 76 | NSCLC | None | B | IL-6 | Interleukin-6 high expression | Binary | Mixed | 9 | 1291 | HR | - | - in 9 studies |
| Liu 2014 | 76 | NSCLC | None | B | IL-8 | Interleukin-8 high expression | Binary | NS | 2 | 142 | HR | <> | <> in 2 studies |
| Martin, 2004 | 969 | LC | (NSCLC) | B | Ki67 | Ki-67 overexpression in NSCLC [*MA restricted to NSCLC studies (29/37); 16/29 eligible)]* | Binary | NS | 16 | NS | HR | **-** | - in 10 studies (8 evaluable); <> in 9 studies; |
| Martin, 2004 | 969 | LC |  | B | Ki67 | Ki-67 overexpression [*37 included studies: 29 NSCLC, 1 SCLC, 2 carcinoid tumours, 5 any histology*] | Binary | NS | 37 | NS | HR |  | - in 15 studies (14 NSCLC, 1 carcinoid tumours); <> in 22 studies |
| Martin, 2004 | 969 | LC | (SCLC) | B | Ki67 | Ki-67 overexpression in SCLC | Binary | NS | 1 | NS | HR |  | - in 1 study |
| Wen 2015 | 5684 | NSCLC | None | B | Ki67 | High Ki-67 expression in proliferating cells | Binary | Retrospective | 28 | 58-4534 | HR |  | - |
| Ellis, 2010 | 7352 | NSCLC | none | B | K-RAS | K-RAS mutations - positive/present | Binary | Retrospective | 3 | 184-493 | NS |  | <> 3 studies |
| Huncharek, 1999 | 1133 | NSCLC |  | B | K-RAS | K-RAS mutation-positive | Binary | prospective | 8 | 44-192 | RR | **-** |  |
| Meng, 2013 | 287 | NSCLC |  | B | K-RAS | K-RAS mutation positive | Binary | NS | 41 | 41-1118 | HR | **-** |  |
| Quinton, 2011 | 6781 | NSCLC | ADC/SCC | B | K-RAS | K-RAS mutations (vs no mutation) | Binary | Prospective | 8 | NS (total 2442) | HR |  | - in 8 studies |
| Ying, 2015 | 57 | NSCLC | None | B | K-RAS | K-RAS mutant | Binary | Prospective | 5 | 53-1466 | HR | **-** |  |
| Ying, 2015 | 57 | NSCLC | None | B | K-RAS | K-RAS mutant | Binary | Prospective | 3 | 188-1466 | HR | **-** |  |
| Tian, 2015 | 4978 | mxdC LC | (NSCLC) | B | lncRNA MALAT1 | Raised MALAT1 expression | Binary | NS | 1 NSCLC | 222 | HR | **-** |  |
| Zhang, 2015 | 5707 | mxdC NSCLC | <IV | B | lncRNA MALAT1 | Long non-coding RNAs metastasis-associated lung adenocarcinoma transcript 1 (LncRNA MALAT1) overexpression | Binary | NS | 3 | 50-102 | HR | **-** |  |
| Kilvaer, 2015 | 5533 | NSCLC |  | B | LVD | High LVD | Binary | Retrospective | 8 | 65-215 | HR | **-** |  |
| Wang, 2012 | 448 | NSCLC |  | B | LVD | High LVD | Binary | Retrospective | 10 | 50-237 | HR | **-** | - in 8 studies (8 evaluable); <> in 4 studies (2 evaluable). |
| Zhu, 2015 | 5610 | mxdC NSCLC | None | B | MALAT1 | High/ over (vs low/ no) expression of Metastasis-associated lung adenocarcinoma transcript 1 (MALAT-1) | Binary | NS | 2 | 31-222 | HR | **-** |  |
| Kong, 2014 | 157 | NSCLC | None | B | MDM2 | Presence (vs absence) of Murine Double Minute 2 (MDM2) gene polymorphism (T309G, rs2279744 T>G) | Binary | NS | 4 | 306-1787 | HR | **-** |  |
| Dimou, 2014 | 88 | NSCLC |  | B | MET | High MET gene copy number | Binary | Retrospective | 9 | 23-906 | HR | **-** |  |
| Guo, 2014 | 134 | NSCLC |  | B | MET | High MET gene copy number | Binary | Mixed | 11 | 61-844 | HR | **-** |  |
| Guo, 2014 | 134 | NSCLC |  | B | MET | MET protein overexpression. | Binary | Retrospective | 13 | 61-883 | HR | **-** |  |
| Xia 2014 | 78 | mxdC LC | None | B | microRNA polymorphisms | mir-146a rs 2910164 | Binary | NS | 12 | NS | HR | <> | <> in 12 studies |
| Xia 2014 | 78 | mxdC LC | None | B | microRNA polymorphisms | mir 149 rs2292832 | Binary | NS | 8 | NS | HR | <> | <> in 8 studies |
| Xia 2014 | 78 | mxdC LC | None | B | microRNA polymorphisms | mir-196a2 rs11614913 | Binary | NS | 14 | NS | HR | - | - in 14 studies |
| Xia 2014 | 78 | mxdC LC | None | B | microRNA polymorphisms | and mir-499 rs3746444 | Binary | NS | 7 | NS | HR | <> | <> in seven studies |
| He, 2013 | 6625 | mxdC LC |  | B | microRNA-155 | High microRNA-155 expression | Binary | Mixed | 5 | 55-637 | HR | **<>** | <> in 3 studies (2 NSCLC, 1 SCLC,);  - in 2 study (1 LC, 1 SSC) |
| Wang, 2013 | 268 | NSCLC | None | B | microRNA-155 | Over-expression of miR-155 | Binary | NS | 7 | 37 - 637 | HR | **-** |  |
| Wang, 2015 | 5537 | NSCLC | <IV | B | microRNA-155 | High (vs low) expression of miR-155 | Binary | NS | 6 | 37-335 | HR | **<>** |  |
| Xu, 2013 | 148 | mxdC LC | <IV | B | microRNA-155 | MicroRNA-155 overexpression | Binary | NS | 3 | 54-639 | HR | **-** |  |
| Yang 2013 | 338 | NSCLC | None | B | microRNA-155 | High expression of microRNA-155 | Binary | NS | 23 | 53-193 | HR | - |  |
| Ma, 2012 | 395 | NSCLC |  | B | microRNA-21 | Elevated MicroRNA-21 in tumour | Binary | NS | 4 | 47-631 | HR | **<>** |  |
| Ma, 2012 | 395 | NSCLC |  | B | microRNA-21 | elevated MicroRNA-21 in serum | Binary | NS | 2 | 70-88 | HR | **-** |  |
| Wang, 2013 | 268 | NSCLC | None | B | microRNA-21 | Over-expression of miR-12 | Binary | NS | 9 | 30 - 631 | HR | **-** |  |
| Yang 2013 | 338 | NSCLC | None | B | microRNA-21 | High expression of microRNA-21 | Binary | NS | 23 | 53-193 | HR | - |  |
| Zhu, 2014 | 120 | mxdC NSCLC | III | B | microRNA-21 | Over/high (vs no/low) expression of mir-21 | Binary | Retrospective | 3 | 88-191 | HR | **-** |  |
| Qian, 2010 | 657 | NSCLC | <IV | B | MMP-2 | Matrix metalloproteinase 2 overexpression | Binary | NS | 11 | 32-218 | HR | **-** |  |
| Liang, 2014 | 183 | NSCLC | I-IV | B | MMP-7 | matrix metalloproteinase-7 (MMP-7) overexpression | Binary | NS | 7 | 66-452 | HR | **-** | <> in3 studies - in 3 studies + in 1 study |
| Wu 2014 | 125 | mxdC NSCLC/SCLC | None | B | MT1-MMP | High expression of Mt1-MMP evaluated by immunohistochemistry | Binary | NS | 11 | 23-549 | HR | - | - in 9 studies;  <> in 2 studies |
| Li, 2015 | 4976 | NSCLC |  | B | mTOR | High mTOR expression | Binary | NS | 4 | 134-172 | HR | **<>** |  |
| Meert, 2002 | 1050 | LC (surR) | (NSCLC) | B | MVD | High MVD count, detected using factor VIII [*12 NSCLC, 2 ADC]* | Binary | NS | 7 | 28-408 | HR | **-** | - in 8 studies (7 NSCLC; 1 ADC); <> in 5 studies (4 NSCLC; 1 ADC); ? in 1 study |
| Meert, 2002 | 1050 | LC (surR) | (NSCLC) | B | MVD | High MVD count, detected using CD34 [*8 NSCLC, 2ADC*] | Binary | NS | 9 | 15-515 | HR | **-** | - in 6 studies (4 NSCLC; 2 ADC); <> in 4 studies (4 NSCLC); |
| Meert, 2002 | 1050 | LC (surR) | (NSCLC) | B | MVD | High MVD count, detected using CD31 [*7 NSCLC, 1 ADC*] | Binary | NS | 7 | 44-407 | HR | **-** | - in 5 studies (4 NSCLC; 1 ADC); <> in 3 studies (3 NSCLC); |
| Trivella, 2007 | 846 | NSCLC | <IV | B | MVD | Increased MVD count (identified via Chalkley method - extra microvessel) | Continuous | Mixed | 6 | 64-1173 | HR | **-** |  |
| Trivella, 2007 | 846 | NSCLC | <IV | B | MVD | High MVD count, identified via all vessel method - 10 extra microvessels) | Continuous | Mixed | 13 | 33-407 | HR | **<>** |  |
| Wu 2015 | 5510 | LC |  | B | NF-kappaB | NF-kB overexpression in solid tumours | Binary | NS | 4 | 88-334 | HR | **<>** | - in four studies |
| Yan, 2014 | 192 | NSCLC | None | B | NSE | High (vs low) Serum levels of neuron-specific enolase (NSE) | Binary | NS | 8 | NR | HR | **<>** |  |
| Zhao, 2013 | 243 | SCLC | None | B | NSE | High/ present (vs low/absent) Serum neuron-specific enolase (NSE) levels | Binary | NS | 11 | NS | OR | **-** |  |
| Liu, 2015 | 5525 | NSCLC | None | B | OPN | Elevated serum- and tissue-based OPN levels | Binary | NS | 10 | 55 - 244 | HR | **-** |  |
| Zou, 2015 | 5829 | NSCLC | III | B | OPN | High (vs low) Osteopontin (OPN) expression | Binary | NS | 5 | 67-244 | HR | **-** |  |
| Lou-Quian, 2013 | 334 | NSCLC | None | B | P16 | P16 promoter hypermethylation | Binary | Retrospective | 17 | 44 - 351 | HR | **-** |  |
| Tong, 2011 | 486 | NSCLC | None | B | P16 | Low expression of P16 in primary lung cancer tissues or metastatic tissues | Binary | Retrospective | 20 | 38 - 219 | HR | **-** |  |
| Xing 2013 | 5058 | mxdC NSCLC | surR | B | P16 | p16 hypermethylation presence (vs absence) | Binary | NS | 11 | 1654 | HR | **-** | - in 11 studies |
| Zhuang, 2011 | 489 | NSCLC | <IV | B | p27 | High (vs low) Protein p27 expression | Binary | NS | 11 | 56-405 | HR | **+** |  |
| Ellis, 2010 | 7352 | NSCLC | none | B | p53 | p53 expression status - positive/overexpression | Binary | Retrospective | 2 | 180-445 | NS |  | - 1 study  <> 1 study |
| Ellis, 2010 | 7352 | NSCLC | none | B | p53 | Presence of p53 mutation | Binary | Retrospective | 2 | 183-445 | NS |  | <> 2 studies |
| Huncharek, 2000 | 1121 | NSCLC |  | B | p53 | Presence of p-53 gene mutations – identified using SSCP/DNA | Binary | NS | 8 | 54-250 | RR | **-** |  |
| Mitsudomi, 2000 | 1110 | NSCLC | surR | B | p53 | P53 overexpression | Binary | NS | 30 | 39-209 | Survival rate | **-** |  |
| Mitsudomi, 2000 | 1110 | NSCLC | surR | B | p53 | Presence of P53 mutations | Binary | NS | 12 | 42-144 | Survival rate | **-** |  |
| Steels, 2001 | 5212 | LC | None | B | p53 | P53 abnormalities | Binary | NS | 74 | NS | HR | **-** | - in 30 studies; <> in 42 studies; + in 2 study. |
| Steels, 2001 | 5212 | LC | (NSCLC) | B | p53 | P53 abnormalities in NSCLC | Binary | NS | 67 | 14-530 (for 50 evaluable in MA) | HR |  | - in 28 studies (26 evaluable); <> / – in 39 studies (24 evaluable) |
| Steels, 2001 | 5212 | LC | (NSCLC-SCC) | B | p53 | P53 abnormalities in SSC | Binary | NS | 9 | 21-88 | HR | - | - in 2 studies;  <> in 7 studies |
| Steels, 2001 | 5212 | LC | (NSCLC-ADC) | B | p53 | P53 abnormalities in ADC | Binary | NS | 9 | 42-200 | HR | - | - in 4 studies;  <> in 5 studies |
| Steels, 2001 | 5212 | LC | (SCLC) | B | p53 | P53 abnormalities in SCLC | Binary | NS | 2 | NS | NS |  | <> in 2 studies |
| Qiu, 2013 | 221 | NSCLC | None | B | p-Akt | Phosphorylated AKT expression | Binary | Prospective | 9 | 46-265 | HR | **-** |  |
| Yang, 2014 | 169 | NSCLC | None | B | p-Akt | phospho-Akt overexpression | Binary | Prospective | 18 | 25-332 | HR | **-** |  |
| Wang, 2015 | 103 | NSCLC | <IV | B | PD-L1 | Over/ high (vs no/ low) expression of Programmed Cell Death-ligand 1 (PD-L1) | Binary | NS | 6 | 109-303 | HR | **-** |  |
| Li, 2015 | 4976 | NSCLC |  | B | p-mTOR | High p-mTOR expression | Binary | NS | 7 | 59-276 | HR | **<>** |  |
| Mascaux, 2005 | 961 | LC | (NSCLC) | B | RAS or p21 | RAS mutation or p21 overexpression in NSCLC (included studies were concerning KRAS2 p21 expression) | Binary | NS | 28 | 21-355 | HR | **-** |  |
| Mascaux, 2005 | 961 | LC | (SCLC) | B | RAS or p21 | RAS mutation or p21 overexpression in SCLC | Binary | NS | 1 | 93 | Survival curves |  | <> In 1 study |
| Mascaux, 2005 | 961 | LC |  | B | RAS or p21 | RAS mutation or p21 overexpression in LC [*29/43 eligible studies were evaluable, but MA restricted NSCLC studies (n=28)]* | Binary | NS | 43 | 21-355 | NS |  | - in 9 studies (7 evaluable); <> in 31 studies (21 evaluable); + in 1 study (evaluable); ? In 2 studies. |
| Wang, 2011 | 534 | NSCLC | surR | B | RASSF1A methylation | Presence of RASSF1A promoter hypermethylation (vs absence) | Binary | Retrospective | 17 | 75 - 351 | HR | **-** |  |
| Zeng, 2015 | 5640 | NSCLC | late | B | RRM1 | Low/negative RRM1 expression level (vs high/positive level) | Binary | Mixed | 3 | 60-75 | OR | **+** |  |
| Xu, 2015 | 5695 | NSCLC | None | B | RUNX3 | Presence of RUNX3 hypermethylation | Binary | NS | 3 | 44-101 | HR | **-** |  |
| Chen, 2013 | 257 | NSCLC |  | B | SOX-2 | SOX2 overexpression | Binary | NS |  | 44-758 | HR | **+** |  |
| Shao, 2015 | 5618 | NSCLC | None | B | SOX-2 | SOX-2 overexpression in tumour tissue | Binary | Retrospective | 7 | 44 - 758 | HR | **+** |  |
| Xu, 2014 | 199 | NSCLC | None | B | STAT3 | High STAT3 expression or phospho-STAT3 | Binary | Prospective | 9 | 40-178 | HR | **-** |  |
| Fan, 2008 | 777 | NSCLC | SurR | B | survivin | Positive survivin expression - non location specific | Binary | Retrospective |  | 43-219 | RR | **-** |  |
| Fan, 2008 | 777 | NSCLC | SurR | B | survivin | level of nuclear survivn | Binary | Retrospective |  | 58-141 | RR | **<>** |  |
| Huang, 2013 | 343 | NSCLC |  | B | survivin | High expression of survivin | Binary | NS | 29 | 43-219 | HR | **-** |  |
| Huang, 2013 | 343 | NSCLC |  | B | survivin | High expression of nuclear survivin | Binary | NS | 6 | 48-144 | HR | **-** |  |
| Sun, 2013 | 6459 | NSCLC | None | B | survivin | Positive Survivin expression | Binary | Retrospective | 4 | 100-264 | OR | **-** |  |
| Xie 2012 | 368 | NSCLC | None | B | survivin | High nuclear survivin expression | Binary | NS | 7 | 48-244 | HR | - |  |
| Zhang, 2012 | 446 | NSCLC | IIIB-IV | B | survivin | Survivin (baculoviral inhibitor of apoptosis repeat-containing 5, BIRC5) overexpression | Binary | NS | 28 | 43-219 | HR | **-** |  |
| Wang 2015 | 5514 | NSCLC | None | B | T-cadherin | T-cadherin hypermethylation | Binary | NS | 4 | 28-161 | HR | **-** | - in four studies |
| Zhu, 2015 | 4969 | NSCLC | IIIB-IV | B | TIMP-2 | High (vs low) expression of tissue inhibitor of metalloproteinase (TIMP-2) | Binary | NS | 5 | 42-148 | HR | **+** |  |
| Knez, 2011 | 525 | SCLC |  | B | topoisomerase II | high protein expression (PE) or gene expression (GE) of topoisomerase II | Binary | NS | 2 | 85-92 | p-value |  | - in 1 study; <> in 1 study |
| Liu, 2013 | 239 | NSCLC | I-IV | B | TS | High expression (vs no or low expression) of Thymidylate synthase | Binary | Mixed | 8 | 28-285 | HR | **-** |  |
| Liu, 2015 | 36 | NSCLC | IIIB-IV | B | TS | High level of thymidylate synthase protein expression (vs low, or positive vs negative) | Binary | Mixed | 20 | 13-193 | HR | **-** |  |
| Wang, 2013 | 247 | NSCLC | IIIA-IV | B | TS | Low / negative TYMS expression | Binary | Mixed | 6 | 24-193 | HR | + | + in 6 studies |
| Berghmans, 2006 | 886 | NSCLC |  | B | TTF-1 | Positive TTF-1 expression | Binary | NS | 8 | 50-284 | HR | **<>** | + in 4 studies; - in 1 study; <> in 5 studies. |
| Yang, 2014 | 165 | NSCLC | None | B | TUBB3 | Class III b-Tubulin expression - positive or high level (vs negative or low level) | Binary | NS | 18 | 24-577 | HR | **-** |  |
| Zhang, 2012 | 420 | NSCLC | IIIB-IV | B | TUBB3 | High/ positive (vs low/ negative) Class III β-tubulin gene expression (mRNA level) | Binary | NS | 7 | 19-93 | MR | **-** |  |
| Ellis, 2010 | 7352 | NSCLC | none | B | Tubulin | Positive Tubulin expression | Binary | Retrospective | 1 | 1149 | NS |  | - 1 study |
| Ellis, 2010 | 7352 | NSCLC | none | B | VCAM-1 | High levels of VCAM-1 | Binary | Retrospective | 1 | 358 | NS |  | - 1 study |
| Buttigliero, 2011 | 494 | mxdC LC | (NSCLC) | B | VDR | Presence of VDR polymorphism | Binary | Prospective | 2 | 294-373 | HR |  | <> in 1 study (early);  + in 1 study (adv). |
| Ellis, 2010 | 7352 | NSCLC | none | B | VEGF | High levels of VEGF | Binary | Retrospective | 1 | 358 | NS |  | - 1 study |
| Zhan, 2009 | 724 | LC | None | B | VEGF | VEGF overexpression | Binary | NS | 44 | 42-335 | HR | **-** |  |
| Kilvaer, 2015 | 5533 | NSCLC |  | B | VEGF subtypes | High expression of VEGF-D | Binary | Retrospective | 4 | 48-335 | HR | **<>** |  |
| Zhan, 2009 | 724 | LC | None | B | VEGF subtypes | VEGF-3/flt-1 overexpression | Binary | NS | 4 | 129-180 | HR | **<>** |  |
| Jiang, 2014 | 144 | NSCLC |  | B | VEGF-C | High VEGF-C expression | Binary | Retrospective | 16 | 49-355 | HR | **-** |  |
| Kilvaer, 2015 | 5533 | NSCLC |  | B | VEGF-C | High expression of VEGF-C | Binary | Retrospective | 17 | 48-335 | HR | **-** |  |
| Zhan, 2009 | 724 | LC | None | B | VEGF-C | VEGF-C overexpression | Binary | NS | 8 | 78-335 | HR | **<>** |  |
| Hu, 2013 | 286 | LC |  | B | VEGFR | High circulating VEGFR | Binary | NS | 17 | 41-451 | HR | **-** |  |
| Kilvaer, 2015 | 5533 | NSCLC |  | B | VEGFR | High expression of VEGFR3 | Binary | Retrospective | 5 | 48-335 | HR | **<>** |  |
| Wu 2012 | 400 | NSCLC | None | B | XRCC1 | XRCC1 gene polymorphisms | Binary | NS | 13 | 57-200 | HR | **-** | - in 13 studies |
| Qiu, 2013 | 242 | NSCLC | None | B | XRCC3 polymorphism | XRCC3 expression | Binary | NS | 10 | 62-355 | HR | **<>** | <> in 10 studies |
| Shen, 2013 | 266 | NSCLC | late | B | XRCC3 polymorphism | Presence of XRCC3 polymorphism | Binary | NS | 4 | 62 - 355 | HR | **<>** |  |

**Abbreviations:** ALDH1 aldehyde dehydrogenase 1; Bcl-2 apoptosis regulator B-Cell lymphoma 2; bFGF basic fibroblast growth factor; Cav-1 caveolin-1 scaffolding protein tumour suppressor gene; CD133 AKA prominin-1. Glycoprotein encoded by PROM1 gene; CD44 Includes CD44 isoforms, CD44s: standard form; CD44v6: CD44 variant 6cell surface glycoprotein; CDH13 cadherin 13; CEA carcinoembryonic antigen; COX-2 cyclooxygenase-2; CRP C-reactive protein; CXCR4 C-X-C chemokine receptor 4; CYFRA 21-1 a cytokeratin 19 fragment; DAPK death-associated protein kinase; E-cadherin epithelial cadherin; EGFR epidermal growth factor receptor; EphA2 EPH receptor A2 gene belongs to the ephrin receptor subfamily of the protein-tyrosine kinase family; ERbeta oestrogen receptor beta; ERCC1 excision repair cross-complementation; ESR1 estrogen receptor 1; ESR2 estrogen receptor 2; EZH2 Enhancer of zeste homolog 2; FGFR1 fibroblast growth factor receptor1; FOXM1 forkhead box M1; HER-2 human epidermal growth factor receptor 2; HIF-1alpha hypoxia inducible factor 1 alpha subunit; HIF-2alpha hypoxia inducible factor 2 alpha subunit; HR hazard ratio; ICAM intercellular adhesion molecule; ICAM1 Intercellular adhesion molecule 1; IGF1R insulin-like growth factor receptor 1; IL-17 interleukin-17; IL6 interleukin 6; IL8 interleukin 8; Ki67 antigen KI-67 protein cellular marker for proliferation; K-RAS Kirsten rat sarcoma viral oncogene homolog; LC lung cancer; LVD lymphatic vascular density; MALAT1 metastasis associated lung adenocarcinoma transcript 1; MDM2 murine double minute 2 gene; MET mesenchymal-epithelial transition; MMP 2 matrix metalloproteinase-2; MMP 7 matrix metalloproteinase – 7; mTOR mammalian target of rapamycin; MVD microvessel density; NF-kappaB nuclear factor-kappaB; NS not stated; NSCLC non small cell lung cancer; NSE neuron-specific enolase; OPN osteopontin; OR odds ratio; p-Akt phosphor-Akt; PD-L1 gene expression of programmed cell death 1 (PD-1) and one of its ligands, PD-L1; p-mTOR phosphorylated mammalian target of rapamycin; RAS resistance to audiogenic seizures; RASSF1A methylation Ras association (RalGDS/AF-6) domain family member 1; RR relative risk; RUNX3 runt related transcription factor 3 hypermethylation; SCLC small cell lung cancer; SOX-2 SRY (sex determining region Y)-box 2; STAT3 signal transducer and activator of transcription 3; TIMP-2 tissue inhibitor of metalloproteinase-2; TS thymidylate synthase; TTF-1 thyroid transcription factor 1; TUBB3 Beta tubulin III; VCAM-1 vascular cell adhesion molecule 1; VDR vitamin D receptor; VEGF vascular endothelial growth factor; XRCC 1 x-ray repair cross-complementing group 1; XRCC 3 x-ray repair cross-complementing group 3

**Table D2: Tumour characteristics**

| **Author, year** | **REV ID** | **LC Type** | **LC Subgroup** | **PF Cat** | **PF** | **PF description** | **Measure** | **Study design** | **Evaluable studies** | **Sample size range** | **Summary Measure** | **Pooled results** | **Narrative synthesis** |
| --- | --- | --- | --- | --- | --- | --- | --- | --- | --- | --- | --- | --- | --- |
| Wang, 2011 | 469 | NSCLC | surR | T | blood vessel invasion | Blood vessel invasion in surgically resected primary tumour samples | Binary | NS | 29 | 51 - 2295 | HR | **-** |  |
| Deng, 2015 | 5645 | NSCLC | N0 | T | BMM | Molecular tumour cell detection within the bone marrow (bone marrow micormetastases) | Binary | NS | 7 | 39-821 | HR | **-** |  |
| Ashworth, 2014 | 105 | NSCLC | oligometastatic | T | Brain metastases | Brain metastases (vs absent) | Binary | Prospective | 20 | 6-262 | HR | **<>** |  |
| Huang, 2013 | 234 | NSCLC |  | T | CTCs | Presence (vs absence) of circulating tumour cells (CTCs) | Binary | NS | 11 | 24-208 | RR | **-** |  |
| Ma, 2012 | 401 | LC |  | T | CTCs | Circulating tumour cells (CTCs) in peripheral blood sampled pre-treatment | Binary | NS | 9 | 28-208 | HR | **-** |  |
| Ma, 2012 | 401 | LC | (NSCLC) | T | CTCs | Circulating tumour cells (CTCs) in peripheral blood sampled pre-treatment in NSCLC | Binary | NS | 7 | 28-208 | HR | **-** |  |
| Ma, 2012 | 401 | LC | (SCLC) | T | CTCs | Circulating tumour cells (CTCs) in peripheral blood sampled pre-treatment in SCLC | Binary | NS | 2 | 50-97 | HR | **<>** |  |
| Ma, 2012 | 401 | LC |  | T | CTCs | Circulating tumour cells (CTCs) in peripheral blood sampled after treatment | Binary | NS | 5 | 50-208 | HR | **-** |  |
| Ma, 2012 | 401 | LC | (NSCLC) | T | CTCs | Circulating tumour cells (CTCs) in peripheral blood sampled after treatment in NSCLC | Binary | NS | 4 | 67-103 | HR | **-** |  |
| Ma, 2012 | 401 | LC | (SCLC) | T | CTCs | Circulating tumour cells (CTCs) in peripheral blood sampled after treatment in SCLC | Binary | NS | 1 | 97 | HR |  | - in 1 study |
| Zhang, 2014 | 81 | SCLC | None | T | CTCs | Baseline Circulating Tumour Cells (higher vs lower) before treatment | Binary | NS | 7 | 30-97 | HR | **-** |  |
| Yu, 2015 | 5489 | NSCLC | None | T | GTV | Small gross tumour volume GTV < 112 cm3 | Binary | NS | 5 | 32-115 | HR | + |  |
| Aboshi, 2014 | 182 | NSCLC | late | T | histology | Percentage of patients in study with squamous cell carcinoma <30 (vs ≥30) | Binary | Prospective | 13 | 39-1217 | OR | **+** |  |
| Ashworth, 2014 | 105 | NSCLC | oligometastatic | T | histology | Large cell carcinoma (vs ADC) | Binary | Prospective | 20 | 6-262 | HR | **-** |  |
| Ashworth, 2014 | 105 | NSCLC | oligometastatic | T | histology | Squamous cell carcinoma (vs ADC) | Binary | Prospective | 20 | 6-262 | HR | **-** |  |
| Ashworth, 2014 | 105 | NSCLC | oligometastatic | T | histology | ‘other’ histology subtypes [not LCC or SCC] (vs ADC) | Binary | Prospective | 20 | 6-262 | HR | **-** |  |
| Carter, 2014 | 5362 | NSCLC | III-IV | T | histology | ADC histology | Binary | Retrospective | 31 | NS | NS |  | + in 5 studies;  <> in 26 studies |
| Quinton, 2011 | 6781 | NSCLC | IIIB-IV | T | histology | Adenocarcinoma (vs Squamous carcinoma/ Non-squamous carcinoma) | Binary | Prospective | 7 | NS (total 5408) | HR |  | + in 7 studies |
| Salah, 2012 | 467 | NSCLC | met (isolated) | T | histology | ADC (vs SCC) | Binary | Retrospective | 51 | NA | HR | **<>** |  |
| Tanvetyanon, 2015 | 23 | NSCLC | sync | T | histology | ADC as the sole histology | Binary | Retrospective | 6 | NS (total 467) | HR | **+** |  |
| Carter, 2014 | 5362 | NSCLC | III-IV | T | extent of mets | Less extensive metastases | Binary | Retrospective | 18 | NS | NS |  | + in 13 studies;  <> in 5 studies |
| Luo, 2015 | 58 | NSCLC | <IV | T | LRF | Local regional failure, LRF (vs distant metastases, DM) | Binary | Mixed | 10 | 30-1402 | RR | **+** |  |
| Ashworth, 2014 | 105 | NSCLC | oligometastatic | T | Lung metastases | Lung metastases (vs absent) | Binary | Prospective | 20 | 6-262 | HR | **<>** |  |
| Luo, 2015 | 58 | NSCLC | <IV | T | LVI | Lymphovascular invasion, LVI (vs non-LVI) | Binary | Mixed | 6 | 18-210 | RR | **-** |  |
| Mollberg, 2014 | 196 | NSCLC | I | T | LVI | Presence of neoplastic cells in arterial, venous or lymphatic lumen | Binary | NS | 20 | 47 - 1,929 | HR | **-** |  |
| Wang, 2012 | 356 | NSCLC | surR | T | LVI | LVI in surgically resected primary tumours that had not received irradiation or chemotherapy prior to surgery | Binary | Retrospective | 33 | 26 - 1,074 | HR | **-** |  |
| Knez, 2011 | 525 | SCLC |  | T | MDR1 polymorphism | MDR1 gene polymorphisms or SNPs | Binary | NS | 2 | NS | p-value |  | In 1 study:  - SNP in abcc1 gene;  + SNP in abcc2 gene;  + Halotypes in abcc2 gene  In 1 study:  <> 'SNP in genes coding for various ABC proteins' |
| Knez, 2011 | 525 | SCLC |  | T | MDR1, MRP1, MRP2 or MVP | High protein expression (PE) or gene expression (GE) of MDR1, MRP1, MRP2 or MVP | Binary | NS | 2 | 9-248 | p-value |  | - in 1 study (MDR1 GE); <> in 1 study (MDR1 PE/MRP1 PE/MRP2 PE) |
| Ashworth, 2014 | 105 | NSCLC | oligometastatic | T | N status | N1 (vs N0) | Binary | Prospective | 20 | 6-262 | HR | **-** |  |
| Ashworth, 2014 | 105 | NSCLC | oligometastatic | T | N status | N2 (vs N0) | Binary | Prospective | 20 | 6-262 | HR | **-** |  |
| Ashworth, 2014 | 105 | NSCLC | oligometastatic | T | N status | N3 (vs N0) | Binary | Prospective | 20 | 6-262 | HR | **-** |  |
| Marchevsky, 2010 | 625 | NSCLC | I-II | T | N status | Isolated tumour cells; Micrometastasis (Node negative vs node positive) | Binary | Retrospective | 14 | NR | OR | <> |  |
| Salah, 2012 | 467 | NSCLC | met (isolated) | T | N status | mediastinal lymph node involvement N2 or N3 (vs no mediastinal lymph node involvement N0 or N1) | Binary | Retrospective | 37 | NA | HR | **-** |  |
| Tanvetyanon, 2013 | 320 | NSCLC | NSCLC (sync, surR) | T | N status | Nodal status: N1 (vs N0) | Binary | NS | 6 | 26-116 | Adj HR | **-** |  |
| Tanvetyanon, 2013 | 320 | NSCLC | NSCLC | T | N status | nodal status: N2 (vs N0) | Binary | NS | 6 | 26-116 | Adj HR | **-** |  |
| Lim, 2010 | 662 | NSCLC | None | T | PLC | Positive (vs negative) pleural lavage cytology | Other | NS | 11 | 36-2950 | HR | **-** |  |
| Saso, 2012 | 427 | NSCLC | surR | T | PLC | Positive pre-resection pleural lavage cytology | Binary | Mixed | 17 | 65 -2,113 | HR | **-** |  |
| Aboshi, 2014 | 182 | NSCLC | late | T | Stage | Percentage of patients in study with stage IV disease <80 (vs ≥80) | Binary | Prospective | 13 | 39-1217 | OR | **<>** |  |
| Ashworth, 2014 | 105 | NSCLC | oligometastatic | T | Stage | IB (vs IA) | Binary | Prospective | 20 | 6-262 | HR | **<>** |  |
| Ashworth, 2014 | 105 | NSCLC | oligometastatic | T | Stage | IIA (vs IA) | Binary | Prospective | 20 | 6-262 | HR | **<>** |  |
| Ashworth, 2014 | 105 | NSCLC | oligometastatic | T | Stage | IIB (vs IA) | Binary | Prospective | 20 | 6-262 | HR | **- (unadj)** |  |
| Ashworth, 2014 | 105 | NSCLC | oligometastatic | T | Stage | IIIA (vs IA) | Binary | Prospective | 20 | 6-262 | HR | **- (unadj)** |  |
| Ashworth, 2014 | 105 | NSCLC | oligometastatic | T | Stage | IIIB (vs IA) | Binary | Prospective | 20 | 6-262 | HR | **- (unadj)** |  |
| Behera, 2016 | 5815 | NSCLC | I-II | T | Stage | Adenocarcinoma In Situ, AIS (vs Minimally Invasive Adenocarcinoma, MIS) | Binary | NS | 11 | 8-110 | survival rate | **<>** |  |
| Carter, 2014 | 5362 | NSCLC | III-IV | T | Stage | Less advanced stage, mainly IIIB (vs IV) | Binary | Retrospective | 37 | NS | NS |  | + in 21 studies;  <> in 16 studies |
| Deghaidy 2005 | 923 | LC | (NSCLC) | T | Stage | Stage I (vs II) in NSCLC | Binary | Mixed | 10 | 23-226 | RR | **+** | + in 5 studies;  <> in 5 studies |
| Deghaidy 2005 | 923 | LC | (SCLC) | T | Stage | Stage I (vs II) in SCLC | Binary | Mixed | 4 | 24-296 | RR | **+** | + in 3 studies;  <> in 1 study |
| Deghaidy 2005 | 923 | LC | (NSCLC) | T | Stage | stage I (vs III) in NSCLC | Binary | Mixed | 7 | 33-1342 | RR | **+** | + in 4 studies,  <> in 3 studies |
| Deghaidy 2005 | 923 | LC | (SCLC) | T | Stage | stage I (vs III) in SCLC | Binary | Mixed | 3 | 27-92 | RR | **+** | + in 2 studies;  <> in 1 study |
| Deghaidy 2005 | 923 | LC | (NSCLC) | T | Stage | Stage II (vs III) in NSCLC | Binary | Mixed | 7 | 57-961 | RR | **+** | + in 3 studies,  <> in 4 studies |
| Deghaidy 2005 | 923 | LC | (SCLC) | T | Stage | Stage II (vs III) in SCLC | Binary | Mixed | 3 | 25-95 | RR | **+** | <> in 3 studies |
| Deghaidy 2005 | 923 | LC | (NSCLC) | T | Stage | Stage III (vs IV) in NSCLC | Binary | Mixed | 6 | 21-2198 | RR | **+** | + in 3 studies,  <> in 3 studies |
| Deghaidy 2005 | 923 | LC | (SCLC) | T | Stage | Stage III (vs IV) in SCLC | Binary | Mixed | 2 | 22-319 | RR | **<>** | <> in 2 studies |
| Salah, 2012 | 467 | NSCLC | met (isolated) | T | Stage | Intra-thoracic stage III (vs stage II or I) | Binary | Retrospective | 36 | NA | HR | **<>** |  |
| Ashworth, 2014 | 105 | NSCLC | oligometastatic | T | Sync mets | Synchronous (vs metachronous) | Binary | Prospective | 20 | 6-262 | HR | **-** |  |
| Salah, 2012 | 467 | NSCLC | met (isolated) | T | Sync mets | synchronous (vs metachronous) | Binary | Retrospective | 62 | NA | HR | **<>** |  |
| Tanvetyanon, 2008 | 799 | NSCLC | met | T | Sync mets | Synchronous (vs metachronous) adrenal metastases | Other | NS | 10 | 4-29 (114 pts) | Median survival | **-** |  |
| Jiang, 2015 | 5 | MPLC |  | T | Sync T | Synchronous MPLC (vs metachronous MPLC) | Binary | Retrospective | 22 | 36-121 | HR | **-** |  |
| Jiang, 2015 | 5 | MPLC |  | T | Sync T | Synchronous MPLC (vs metachronous MPLC) starting from diagnosis of 1st metachronous tumour. | Binary | Retrospective | 11 | 36-121 | HR | **-** |  |
| Jiang, 2015 | 5 | MPLC |  | T | Sync T | Synchronous MPLC (vs metachronous MPLC) starting from diagnosis of 2nd metachronous tumour. | Binary | Retrospective | 9 | 90-11 | HR | **<>** |  |
| Tanvetyanon, 2013 | 320 | NSCLC | NSCLC (sync, surR) | T | T loc | tumour side: bilateral (vs unilateral) | Binary | NS | 6 | 26-116 | Adj HR | **+** |  |
| Salah, 2012 | 467 | NSCLC | ADC/SCC/other | T | T status | smaller primary tumours: T1 or T2) (vs larger tumours T3 or T4) | Binary | Retrospective | 62 | NS | HR | **<>** |  |
| Salah, 2012 | 467 | NSCLC | met (isolated) | T | visceral mets | non-visceral (vs visceral metastasis) | Binary | Retrospective | 62 | NA | HR | **<>** |  |
| Huang, 2015 | 95 | NSCLC | I-II | T | VPI | Presence (vs absence) of Visceral Pleural Invasion | Binary | Retrospective | 15 | 73-6048 | HR | **-** |  |
| Jiang, 2015 | 4951 | NSCLC | NOMO | T | VPI | Visceral pleural invasion (vs without visceral pleural invasion) | Binary | Retrospective | 13 | 142-16315 | OR | **-** | - in 8 studies,  <> in 5 studies |
| Luo, 2015 | 58 | NSCLC | <IV | T | VPI | Visceral pleural invasion, VPI (vs non-VPI) | Binary | Mixed | 5 | 198-1402 | RR | **-** |  |

**Abbreviations:** ADC adenocarcinoma; adj adjusted; BMM bone marrow metastasis; CTCs circulating tumour cells; DM distant metastases; GTV gross tumour volume; HR hazard ratio; LC lung cancer; LRF local regional failure ; mets metastases; NS not stated; NSCLC non small cell lung cancer; SCC squamous cell carcinoma; Sync mets Synchronous metastases;Sync T Synchronous tumours; T Tumour;VPI visceral pleural invasion

**Table D3: Metabolic criteria**

| **Author, year** | **REV ID** | **LC Type** | **LC Subgroup** | **PF Cat** | **PF** | **PF description** | **Measure** | **Study design** | **Evaluable studies** | **Sample size range** | **Summary Measure** | **Pooled results** | **Narrative synthesis** |
| --- | --- | --- | --- | --- | --- | --- | --- | --- | --- | --- | --- | --- | --- |
| Nair, 2009 | 706 | NSCLC | I-II | MC | FDG uptake | High (vs low) 18F-fluorodeoxyglucose (FDG) uptake | Binary | Mixed | 5 | 36-380 | HR |  | - in 3 studies <> in 2 studies |
| Im, 2015 | 14 | NSCLC |  | MC | MTV, TGL | High metabolic tumour volume, MTV | Binary | Retrospective | 7 | 39-529 | HR | **-** |  |
| Im, 2015 | 14 | NSCLC |  | MC | MTV, TGL | High total lesion glycolysis ,TGL | Binary | Retrospective | 6 | 39-529 | HR | **-** |  |
| Liu, 2016 | 5680 | NSCLC | surR | MC | MTV, TGL | High levels of MTV | Binary | Retrospective | 4 | 59 - 529 | HR | **-** |  |
| Liu, 2016 | 5680 | NSCLC | surR | MC | MTV, TGL | High levels of TGL | Binary | Retrospective | 5 | 59 - 259 | HR | **-** |  |
| De Geus-Oui, 2007 | 817 | NSCLC |  | MC | SUV | Low FDG uptake, as measured by FDG-PET at diagnosis (Low SUV) | Binary | NS | 11 | 38-315 | HR |  | + in 11 studies |
| De Geus-Oui, 2007 | 817 | NSCLC |  | MC | SUV | Low FDG uptake, as measured by FDG-PET after induction of treatment (low SUV/ negative FDG-PET/ high metabolof rate of glucose) | Binary | NS | 4 | 47-1136 | HR |  | + in 4 studies |
| De Geus-Oui, 2007 | 817 | NSCLC |  | MC | SUV | Low FDG uptake, as measured by FDG-PET in recurrent disease (low SUV/ negative FDG-PET) | Binary | NS | 2 | 62-63 | HR |  | + in 2 studies |
| Berghmans, 2008 | 805 | LC | <IV | MC | SUVMax (FDG-PET) | High (vs low) Standardized Uptake Value (SUVmax) measured on Fluorodeoxyglucose Positron Emission Tomography (FDG-PET) | Binary | NS | 13 | 38-315 | HR | **-** |  |
| Im, 2015 | 14 | NSCLC |  | MC | SUVMax (FDG-PET) | Hight SUVmax | Binary | Retrospective | 4 | NS | HR | **-** |  |
| Liu, 2016 | 5680 | NSCLC | surR | MC | SUVMax (FDG-PET) | High SUVmax | Binary | Mixed | 19 | 57 - 487 | HR | **-** |  |
| Na, 2014 | 133 | NSCLC | <IV | MC | SUVMax (FDG-PET) | Pre-RT Primary tumour maximum standardized uptake value (SUVmax)-based biomarker [(2-[18F]-Fluorodeoxyglucose (FDG) positron emission tomography (PET/CT) (F-FDG PET/CT)] (High vs low uptake) | Binary | NS | 8 | 46-132 | HR | **-** |  |
| Na, 2014 | 133 | NSCLC | <IV | MC | SUVMax (FDG-PET) | Post-RT primary tumor maximum standardized uptake value (SUVmax)-based biomarker [(2-[18F]-Fluorodeoxyglucose (FDG) positron emission tomography (PET/CT) (F-FDG PET/CT)] (High vs low uptake) | Binary | NS | 3 | 46-132 | HR | **-** |  |
| Paesmans, 2010 | 667 | NSCLC | None | MC | SUVMax (FDG-PET) | High levels of SUV | Binary | NS | 21 | 19-487 | HR | - |  |
| Vansteenkiste, 2004 | 982 | LC | (NSCLC) | MC | SUVMax (FDG-PET) | PET stage (vs CT stage or conventional staging) at diagnosis | Binary | Mixed | 2 | 80 - 152 | median survival |  | + in 2 studies |
| Vansteenkiste, 2004 | 982 | LC | (NSCLC) | MC | SUVMax (FDG-PET) | Low SUVMax (FDG-PET) (vs high; different cut-off points used) at diagnosis | Binary | Retrospective | 4 | 73 - 155 | median survival |  | + in 4 studies |
| Vansteenkiste, 2004 | 982 | LC | (NSCLC) | MC | SUVMax (FDG-PET) | Negative PET or no metabolic response (vs positive PET or metabolic response) after treatment | Binary | Prospective | 2 | 46 - 113 | median survival |  | + in 2 studies |

**Abbreviations:** HR hazard ratio; LC lung cancer; MTV/ TGL metabolic tumour volume, total lesion glycolysis; mxdC mixed cancer; NS not stated NSCLC non small cell lung cancer; SUV standardized uptake value; SUVMax (FDG-PET) maximum standardized uptake value

**Table D4: Clinical characteristics or routinely assessed biological variables**

| **Author, year** | **REV ID** | **LC Type** | **LC Subgroup** | **PF Cat** | **PF** | **PF description** | **Measure** | **Study design** | **Evaluable studies** | **Sample size range** | **Summary Measure** | **Pooled results** | **Narrative synthesis** |
| --- | --- | --- | --- | --- | --- | --- | --- | --- | --- | --- | --- | --- | --- |
| Carter, 2014 | 5362 | NSCLC | III-IV | B/C | albumin | Lower albumin levels | Binary | Retrospective | 54 | 4 | NS |  | - in 4 studies |
| Gupta, 2010 | 610 | mxdC LC |  | B/C | albumin | High (or normal) pretreatment serum albumin (vs low) [1/10 study: SCLC + NSCLC] | Binary | Mixed | 10 | 101-150 | RR |  | + in 9 studies; <> in 1 study. |
| Gupta, 2010 | 610 | mxdC LC | (NSCLC) | B/C | albumin | High pretreatment serum albumin in NSCLC | Binary | Mixed | 7 | 101-261 | RR |  | + in 7 studies |
| Gupta, 2010 | 610 | mxdC LC | (SCLC ) | B/C | albumin | High pretreatment serum albumin in SCLC | Binary | Mixed | 2 | 207-341 | RR |  | + in 2 studies |
| Yang, 2013 | 318 | NSCLC | None | P/C | BRCA1 | High/positive BRCA1 expression | Binary | NS | 7 | 42-769 | HR | **-** |  |
| Ma, 2014 | 190 | LC |  | C | D-dimer level | High serum D-dimer level | Binary | Retrospective | 10 | 56-343 | HR | **-** |  |
| Miao, 2012 | 6341 | ADC | surR | C | GGO | High ratio (>50%) of ground glass opacity (GGO) identified on CT | Binary | NS | 7 | 52-260 | HR | **+** |  |
| Carter, 2014 | 5362 | NSCLC | III-IV | C | Hb | Higher haemoglobin concentration | Binary | Retrospective | 9 | NS | NS |  | + in 5 studies;  <> in 4 studies |
| Sun, 2015 | 5469 | NSCLC | None | C | LNR | Higher LNR | Binary | Retrospective | 11 | 75 - 11,324 | HR | **-** |  |
| Zhao, 2015 | 5531 | LC | Mixed | C | NLR | High (vs Low) neutrophil to lymphocyte ratio | Binary | NS | 21 | 59-1238 | HR | **-** |  |
| Zhou, 2014 | 33 | mxdC NSCLC | None | C | PLR | Elevated Platelet Lymphocyte Ratio (PLR) | Binary | NS | 3 | 94-210 | HR | **-** |  |
| Liu, 2013 | 332 | NSCLC | None | O/C | Skin rash | Skin rash (vs no rash) resulting from EGFR Tyrosine Kinase Inhibitor (TKI) administration | Binary | Prospective | 17 | 23-2983 | HR | **+** |  |
| Petrelli, 2012 | 389 | NSCLC | None | O/C | Skin rash | Skin rash occurrence and greater severity in patients treated with erlotinib and gefitinib | Binary | Mixed | 7 | NS | HR | **+** |  |
| Christopoulos, 2013 | 5789 | LC | none | C | TB | Active tuberculosis, TB (vs no TB) | Binary | Retrospective | 5 | 4-56 | Median survival |  | - 1 study;  ? in 4 studies (no comparative data) |
| Buttigliero, 2011 | 494 | mxdC LC | (NSCLC) | C | VitD level | Low serum VitD | Binary | Prospective | 2 | 294-447 | HR |  | <> in 2 studies |

**Abbreviations:** BRCA BRCa homolog (tumour suppressor gene BRCA); GGO ground glass-opacity; Hb haemoglobin level; HR hazard ratio LC lung cancer; LNR lymph node ratio; mxd C mixed cancer; NLR neutrophil to Lymphocyte Ratio; NS not stated; NSCLC non small cell lung cancer; PLR platelet Lymphocyte Ratio. Inflammation parameters; RR relative risk; TB tuberculosis; VitD level Serum vitamin D level; WBC white blood cell count.

**Table D5: Patient characteristics**

| **Author, year** | **REV ID** | **LC Type** | **LC Subgroup** | **PF Cat** | **PF** | **PF description** | **Measure** | **Study design** | **Evaluable studies** | **Sample size range** | **Summary Measure** | **Pooled results** | **Narrative synthesis** |
| --- | --- | --- | --- | --- | --- | --- | --- | --- | --- | --- | --- | --- | --- |
| Aboshi, 2014 | 182 | NSCLC | late | P | age | Percentage of patients in study with average age <63 (vs ≥63), | Binary | Prospective | 13 | 39-1217 | OR | **+** |  |
| Carter, 2014 | 5362 | NSCLC | III-IV | P | age | older age | Binary | Retrospective | 37 | NS | NS |  | + in 4 studies;  <> in 33 studies |
| Tanvetyanon, 2013 | 320 | NSCLC | NSCLC (sync, surR) | P | age | Increased age (per year) | Categorical | NS | 6 | 26-116 | Adj HR | **-** |  |
| Carter, 2014 | 5362 | NSCLC | III-IV | P | BMI | Less weight loss or normal BMI | Binary | Retrospective | 21 | NS | NS |  | + in 11 studies;  <> in 10 studies |
| Carter, 2014 | 5362 | NSCLC | III-IV | P | comorbidities | Less comorbidities | Binary | Retrospective | 9 | NS | NS |  | + in 3 studies;  <> in 6 studies |
| Soo, 2011 | 520 | NSCLC | late | P | ethnicity | Asian ethnicity (vs Caucasian) | Binary | Prospective | 391 arms | NS | median survival | **+** |  |
| Aboshi, 2014 | 182 | NSCLC | late | P | gender | Percentage of patients in study being male <70 (vs ≥70) | Binary | Prospective | 13 | 39-1217 | OR | **+ (unadj)**  **<> (adj)** |  |
| Carter, 2014 | 5362 | NSCLC | III-IV | P | gender | female | Binary | Retrospective | 43 | NS | NS |  | + in 17 studies;  <> in 26 studies |
| Nakamura, 2011 | 481 | NSCLC | None | P | gender | Female gender | Binary | NS | 38 | 45-19072 | HR | **+** |  |
| Salah, 2012 | 467 | NSCLC | met (isolated) | P | gender | Male (vs female) | Binary | Retrospective | 62 | NA | HR | **<>** |  |
| Tanvetyanon, 2013 | 320 | NSCLC | NSCLC (sync, surR) | P | gender | female | Binary | NS | 6 | 26-116 | Adj HR | **+** |  |
| Carter, 2014 | 5362 | NSCLC | III-IV | P | previous treatment response | Response to previous treatment | Binary | Retrospective | 9 | NS | NS |  | + in 9 studies |
| Aboshi, 2014 | 182 | NSCLC | late | P | PS | Percentage of patients in study with ECOG PS1 <60 (vs ≥60) | Binary | Prospective | 13 | 39-1217 | OR | **<>** |  |
| Carter, 2014 | 5362 | NSCLC | III-IV | P | PS | Better PS, ECOG 0-1/KPS<70 (vs ≥2, or vs ≥80) | Binary | Retrospective | 47 | NS | NS |  | + in 36 studies;  <> in 11 studies |
| Carter, 2014 | 5362 | NSCLC | III-IV | P | QoL | Better pre-treatment health related QoL/fewer symptoms | Binary | Retrospective | 6 | NS | NS |  | + in 6 studies |
| Montazeri, 2009 | 703 | mxdC NSCLC/SCLC |  | P | QoL | Pre-treatment (baseline) quality of life | Other | NS | 26 | 30-651 | NS |  | + in 24 study;  <> in 2 studies |
| Carter, 2014 | 5362 | NSCLC | III-IV | P | Smoking status | Less/no smoking status | Binary | Retrospective | 9 | NS | NS |  | + in 6 studies;  <> in 3 studies |
| Florou, 2014 | 51 | mxdC NSCLC/SCLC | Early/limited | P | Smoking status | non-smoker (vs smoker) | Binary | Retrospective | 1 | 215 | NS |  | + in 1 study |
| Florou, 2014 | 51 | mxdC NSCLC/SCLC | Early/limited | P | Smoking status | ex-smoker - quit smoking at or after diagnosis (vs current smoker) | Binary | Prospective | 1 | 284 | NS |  | + in 1 study |
| Florou, 2014 | 51 | mxdC NSCLC/SCLC | Early/limited | P | Smoking status | never smoked (vs ex- or current smoker) | Binary | Prospective | 2 | 238-284 | NS |  | + in 1 study; <> in 1 study (+Women) |
| Florou, 2014 | 51 | mxdC NSCLC/SCLC | Early/ limited | P | Smoking status | ex-smoker - quit smoking before diagnosis (vs current smoker or never smoked) | Binary | Prospective | 1 | 543 | NS |  | <> in 1 study |
| Parsons, 2010 | 695 | LC | (NSCLC) | P | Smoking status | Continued smoking after diagnosis (vs quit smoking) in NSCLC | Binary | Mixed | 4 | 93-311 | HR | **<> unadj  - adj (1 study)** |  |
| Parsons, 2010 | 695 | LC | (SCLC) | P | Smoking status | Continued smoking after diagnosis (vs quit smoking) in SCLC | Binary | Retrospective | 2 | 70-611 | HR | **-** |  |

**Abbreviations:** Adj adjusted; BMI body mass index; HR hazard ration; LC lung cancer; mxdC mixed cancer; NS not stated; NSCLC non-small cell lung cancer; OR odds ratio; PS performance status; QoL quality of life; SCLC small cell lung cancer; unadj unadjusted

**Table D6: Healthcare provider and system**

| **Author, year** | **REV ID** | **LC Type** | **LC Subgroup** | **PF Cat** | **PF** | **PF description** | **Measure** | **Study design** | **Evaluable studies** | **Sample size range** | **Summary Measure** | **Pooled results** | **Narrative synthesis** |
| --- | --- | --- | --- | --- | --- | --- | --- | --- | --- | --- | --- | --- | --- |
| Slatore, 2010 | 621 | LC | None | SD | insurance status | Medicaid or no insurance (vs private, non-Medicaid or other funded) | Binary | NS | 4 | 3,702-13,649 | HR/OR |  | -in 4 studies |
| Slatore, 2010 | 621 | LC | None | SD | insurance status | Commercial or other insurance (vs private insurance) | Binary | NS | 2 | 336-1403 | HR/OR |  | <> in 2 studies |
| Slatore, 2010 | 621 | LC | None | SD | insurance status | Medicaid/Medicare (vs Medicare) | Binary | NS | 2 | 3,094-26,073 | HR |  | -in 2 studies |
| Slatore, 2010 | 621 | LC | None | SD | insurance status | Medicare health maintenance organisation service status (vs Medicare Fee for Service) | Binary | NS | 1 | 10,229 | HR |  | <> in 1 study |
| Prades, 2015 | 5807 | mxdC LC | None | SD | MDT | MDT patient management | Binary | Prospective | 1 | NS | Survival rate |  | + in 1 study (NSCLC) |
| von Meyenfeldt, 2012 | 414 | LC | None | SD | procedural volume | High hospital annual volume of surgical resections (vs low volume) – post operative mortality | Binary | NS | 10 | 987-90088 | OR | **+** | + in 6 studies;  <> in 5 studies |
| von Meyenfeldt, 2012 | 414 | LC | None | SD | procedural volume | High hospital annual volume of surgical resections (vs low volume) – overall survival | Binary | NS | 7 | 1097-40754 | HR | **<>** | + in 5 studies;  - In 1 study;  <> in 2 studies |
| von Meyenfeldt, 2012 | 414 | LC | None | SD | procedural volume | High annual surgeon procedural volume (vs low volume) – post operative mortality | Binary | NS | 2 | 4841-24092 | OR | **<>** | + in 2 studies |
| von Meyenfeldt, 2012 | 414 | LC | None | SD | Surgeon | Surgeon specialty: general thoracic surgeon (vs general surgeon) – post operative mortality | Binary | NS | 3 | 19745-86538 | OR | **+** | + in 1 study;  <> in 2 studies |
| von Meyenfeldt, 2012 | 414 | LC | None | SD | Surgeon | Surgeon specialty: general thoracic surgeon (vs general surgeon) – overall survival | Binary | NS | 2 | 1097-19745 | HR | **<>** | + in 1 study;  <> in 1 studies |
| von Meyenfeldt, 2012 | 414 | LC | None | SD | Surgeon | Surgeon specialty: cardiothoracic surgeon (vs general surgeon) – post operative mortality | Binary | NS | 3 | 19745-86538 | OR | **+** | + in 1 study;  <> in 2 studies |
| von Meyenfeldt, 2012 | 414 | LC | None | SD | Surgeon | Surgeon specialty: cardiothoracic surgeon (vs general surgeon) – overall survival | Binary | NS | 2 | 1097-19745 | HR |  | + in 1 study;  <> in 1 studies |
| Neal, 2015 | 8441 | mxdC LC |  | SD | timeliness of care | Shorter diagnostic interval: time from first seen in primary care to diagnosis | NS | Mixed | 4 | 122-378 |  |  | <> in 3 studies; + in 1 study |
| Neal, 2015 | 8441 | mxdC LC | (NSCLC) | SD | timeliness of care | Shorter treatment interval: time from first seen in primary care to treatment | NS | Retrospective | 2 | 415-495 | NS |  | <> in 1 study; - in 1 study |
| Neal, 2015 | 8441 | mxdC LC | (NSCLC) | SD | timeliness of care | Shorter patient interval: time from symptom onset to first seen in primary | NS | Retrospective | 2 | 122-7358 | NS |  | <> in 1 study; - in 1 study |
| Neal, 2015 | 8441 | mxdC LC |  | SD | timeliness of care | Shorter time from symptom onset to diagnosis | NS | Retrospective | 1 | 566 |  |  | + in 1 study |
| Neal, 2015 | 8441 | mxdC LC |  | SD | timeliness of care | Shorter time from symptom onset to treatment | NS | Retrospective | 1 | 103 | NS |  | <> in 1 study |
| Neal, 2015 | 8441 | mxdC LC | (NSCLC) | SD | timeliness of care | Shorter time from symptom onset to being seen in specialist care | NS | Retrospective | 1 | 415 | NS |  | <> in 1 study |
| Olsson 2009 | 722 | LC | None | SD | timeliness of care | Shorter intervals to diagnosis or treatment | NS | Mixed | 15 | NS | NS |  | - in 4 studies;  <> in 8 studies;  + in 3 studies |

**Abbreviations:** BMI body mass index; HR hazard ratio; LC lung cancer; MDT multidisciplinary team; MxdC mixed cancer; NSCLC non small call lung cancer; NS not stated; OR odds ratio; PS performance status; RR relative risk

**Table D7: Prognostic factors classified as ‘other’**

| **Author, year** | **REV ID** | **LC Type** | **LC Subgroup** | **PF Cat** | **PF** | **PF description** | **Measure** | **Study design** | **Evaluable studies** | **Sample size range** | **Summary Measure** | **Pooled results** | **Narrative synthesis** |
| --- | --- | --- | --- | --- | --- | --- | --- | --- | --- | --- | --- | --- | --- |
| Luan, 2014 | 171 | LC | I-II | O | Blood transfusion | Perioperative allogenic blood transfusion | Binary | Retrospective | 14 | 30-731 | RR | **-** |  |
| Wang 2014 | 180 | LC | surR | O | Blood transfusion | Perioperative blood transfusion | Binary | Mixed | 14 | 48-856 | HR | **-** | - in 14 studies |
| Salah, 2012 | 467 | NSCLC | met (isolated) | P | Previous treatment | perioperative chemotherapy (vs no perioperative chemotherapy) | Binary | Retrospective | 62 | NA | HR | **<>** |  |
| Ashworth, 2014 | 105 | NSCLC | oligometastatic | O | Surgical treatment | Surgical treatment for primary LC (vs non-surgical) | Binary | Prospective | 20 | 6-262 | HR | **+ (unadj)** |  |

**Abbreviations:** HR hazard ratio; LC lung cancer; MDT multidisciplinary team; mxdC mixed cancer; NS not stated; NSCLC non small cell lung cancer; RR relative risk; surR surgical resection; unadj unadjusted
