# Supplementary material for: Pragmatic methods for reviewing exceptionally large bodies of evidence: systematic mapping review and overview of systematic reviews using lung cancer survival as an exemplar
Source: Syst Rev. 2019 Jul 16;8:171. doi: 10.1186/s13643-019-1087-4 (PMC6631880; doi:10.1186/s13643-019-1087-4)
Supplement: Supplementary file 5 — Appendix E. Description and results of reviews that aimed to evaluate any prognostic factor. Table E1. Summary of included reviews that considered the inclusion of any prognostic factor. Table E2. Modifiable prognostic factors evaluated by reviews that investigated ‘any’ prognostic factor associated with survival. (DOCX 22 kb) [file 13643_2019_1087_MOESM5_ESM.docx]

**APPENDIX E: DESCRIPTION AND RESULTS OF REVIEWS THAT AIMED TO EVALUATE ANY PROGNOSTIC FACTOR**

Three included reviews, which aimed to identify ‘any’ prognostic factor associated with survival, included over 50 factors and their findings were therefore, summarised separately. The reviews are summarised in Table E1 and their findings relating to potential modifiable factors are presenting in Table E2.

Brundage et al, 2002 identified weight loss, performance status, and stage as an ‘essential factor’ for decision making in patients with both advanced and resected NSCLC. The prominent prognostic factors identified by Berghmans et al, 2011, for patients with stage III NSCLC were: distinction between stage IIIA and IIIB, performance status, age, gender, weight loss, and some characteristics describing locoregional extension of the tumour. They also concluded that there is a place for the standardized uptake value (SUV) as a prognostic factor for survival in early NSCLC, but its role in stage III NSCLC needs further assessed, and that some new biomarkers involved in cell cycle regulation or in apoptosis have also been shown to have potential value. The review by Ashworth et al, 2013 limited inclusion to patients with oligometastatic NSCLC. Highly significant factors associated with improved survival included: definitive treatment of the primary tumour, nodal-stage, and disease-free interval of at least 6-12 months.

**Table E1: Summary of included reviews that considered the inclusion of any prognostic factor**

| **Author, year [Review ID]** | **Brundage, 2002 [1051]** | **Berghmans, 2011 [8434]** | **Ashworth, 2013 [237]** |
| --- | --- | --- | --- |
| **Review method** | SR – narrative synthesis | SR and review of MAs – narrative synthesis | SR – narrative synthesis |
| **Search dates** | 1990-2001  *Original search 1990-1999, extended to June 2001 in response to peer-review comments*. | Search year not stated, but included studies published between 1997-2009 | 1985-2012 |
| **Overall aim** | To provide an overview of the breadth of prognostic factors described to date, to analyse patterns in the design of this literature, to highlight problematic aspects of the studies, and to advocate for appropriate directions of future research. | To review and summarise the available evidence about prognostic factors in stage III NSCLC. | To describe outcomes and prognostic factors that identify patients with oligometastatic NSCLC who may benefit from aggressive treatment strategies.  *The study also evaluated long-term survival in pts with oligometastatic NSCLC (PROGRESS Theme 1*). |
| **Prognostic research theme (PROGRESS)** | 2 | 2 | 1, 2 |
| **Lung cancer type** | NSCLC  Separate results presented for advanced disease, surgically resected patients, and early stage (resectable; treatment naive) disease; representing 3 common presenting clinical scenarios. More in-depth evaluation presented for early stage resected NSCLC, based on studies reporting multivariate analysis (this included tumour cell type as a PF). | NSCLC (stage III)  Review of MAs considered any NSCLC stage, but findings of those specifically dealing with stage III also reported separately.  To be homogeneous only studies that explicitly reported using the 4^th^ - 7th international staging system of the UICC were included. | Oligometastatic NSCLC  The majority of included patients (82%) had a controlled primary tumour and 60% of studies included patients with brain metastases only.  *39/49 studies reported histology of primary tumour: 59% ADC, 20% SCC, 21% other (large cell/ neuroendocrine/ not specified).* |
| **No. of factors considered** | Any factor considered. Included studies identified 169.  PFs were grouped as tumour-related and host-related factors, and presented under the headers: essential for decision making, additional factors that may refine the accuracy of prognosis, and new or promising factors.  All PFs for early stage (resected) NSCLC examined in studies that report at least one multivariate analysis were summarised in a table, with factors categorised into seven groups: anatomical features, histologic features, serum markers, proliferation, other biological features, patient features, and other. | Any factor considered, with >50 variables identified.  PFs were categorised into three groups: conventional prognostic factors (39 studies), metabolic criteria (2 studies), and new biomarkers (15 studies).  In order to try to reduce heterogeneity in patient populations ‘conventional prognostic factors’ were further subdivided according to administered treatment (surgical, n=20; and medical series, n=19). Conventional prognostic factors included anatomical factors, clinical and sociodemographic parameters and some routinely assessed biological variables that are commonly used and not investigational. | Any factor considered. The total number identified not stated. 18 PFs were identified as significant by 2 or more studies using multivariate analysis.  PFs were defined as ‘highly significant’ if they were assessed by multivariate analysis in more than 2 studies and significant in ≥50% of studies; ‘moderately significant’ if they were assessed only in 2 studies and found to be significant in ≥50%; and ‘occasionally significant’ if they were assessed in >2 studies and found to be positive in <50% of the studies. |
| **Number of included studies** | 887 | 39 | 23 evaluating PFs for survival (49 included in main review). |
| **Limited study inclusion according to type of analysis?** | 176/887 included studies reported multivariate analyses. 153 studies of early-stage NSCLC reported multivariate analyses. | In the Methods it was noted that inclusion limited to multivariate analysis, but univariate and multivariate analysis included in results for ‘new’ biomarkers. | Analysis of PFs limited to studies reporting multivariate analysis. Studies with fewer than 10 cases were also excluded. |
| **Overall results** | The median no. of factors, per study, that were significantly associated with patient survival rate in univariate analyses was 4 (range, 1 to 12), and in multivariate analyses this was 2 (range, 0 to 6). The median no. of factors that were independently predictive of patient survival in multivariate models was 2 (range, 0 to 6). The median number of studies examining each PF was 1 (range, 1 to 105).  *Essential factors for patients with advanced NCSLC*  Host related: weight loss, performance status. Tumour related: stage III vs IV, hypercalcaemia, SVCO.  *Essential factors for patient with surgically resected NSCLC*  Host related: weight loss, performance status. Tumour related: stage, N factor, hypercalcaemia, SVCO.  *For early stage disease (clinically respectable; treatment naive)* – studies focused on factors that estimate the patient’s likelihood of surviving pulmonary resection.  Factors found to be significantly associated with survival most often (excluding anatomic stage, and factors examined in fewer than three studies):   - markers of angiogenesis (13 of 16 studies; 81%), - p21 status (4 of 5 studies; 80%), - status of the serum assay for detection of the cytokeratin 19 fragment (4 of 5 studies; 80%), - status of the argyrophilic nucleolar organizer region (3 of 4 studies; 75%), - p185 status (3 of 4 studies; 75%), - Ki-67 status (4 of 6 studies; 67%), - vascular endothelial growth factor status (7 of 11 studies; 64%), - vessel invasion (11 of 21 studies; 52%), and - p53 status (16 of 38 studies; 42%). | The number of factors found to be significant within each study ranged from 0 to 5 for surgical series and 1 to 10 for medical series. The most prominent prognostic factors were: subgroup of stage (distinction between stage IIIA and IIIB), performance status, age, gender, weight loss, and some characteristics describing locoregional extension of tumour. There is a potential prognostic role for new metabolic (standardized uptake value SUV) and biological factors (biomarkers) such as p53, Bcl-2, the apoptotic index, hMSH2, cyclin D1 or DR5 receptor of the rhTRAIL. | Highly significant PFs associated with improved survival were: definitive treatment of the primary tumour, N-stage, and disease-free interval of at least 6-12 months.  Moderately significant PFs associated with improved survival were: smaller primary tumours (1-3cm), the use of PET-CT scan (vs CT), lobectomy (vs pneumonectomy) and RPA classification 1 (vs RPA 2).  Moderately significant PFs associated with decreased survival were: Presence of extracranial mets  Occasionally significant prognostic factors were: ADC histology, younger age, per-operative CTX, stage; single oligometastases; gender (not specified), T stage, synchronous (vs metachronous) oligometastases; visceral or ipsilateral location of oligometastases, and use of whole brain RT. |
| **Author’s conclusions** | While the breadth of prognostic factors studied in the literature is extensive, the scope of factors evaluated in individual studies is inappropriately narrow. Individual studies are typically statistically underpowered and are remarkably heterogeneous with regard to their conclusions. Larger studies with clinically relevant modelling are required to address the usefulness of newly available prognostic factors in defining the management of patients with NSCLC. | Few PFs have been well evaluated in stage III NSCLC. New studies, taking into account the modifications derived from the 7th international staging system of the UICC, have to be performed. | This review identified several prognostic factors for survival that were consistently found to be significant on multivariate analyses in the literature, and these mostly reflect the status of intra-thoracic disease: definitive treatment/control of the primary tumour, intra-thoracic N-stage and a disease free interval of at least 6–12 months. We propose that these factors be used in future prognostic models to identify those oligometastatic patients who are most likely to be long-term survivors. |
| **Was study quality assessed** | No | No | No |
| **Was level of evidence assessed** | No. Limitations and heterogeneous nature of evidence base discussed | No. Limitations and heterogeneous nature of evidence base discussed in depth. | Partial; PFs were graded as highly, moderately, or occasionally significant based on number of studies that evaluated them and whether their results were significant. Limitations and heterogeneous nature of evidence base discussed. The authors also recommended the use of individual patient data meta-analysis, which they went on to conduct *(See Ashworth, 2014 [ID 105])* |
| **Overall quality of review** | **Poor** | **Poor** | **Moderate** |
| **OVERAL comments** | MEDLINE was the only database searched. But considered as a seminal paper; frequently referenced. | MEDLINE was the only database searched, and no search terms or dates were not provided. But included a review of MAs | Search based on MEDLINE and EMBASE. Review conducted according to PRISMA guidance. |

**Abbreviations**: C clinical stage; CT computed tomography; CTX chemotherapy; MA meta-analysis; NSCLC non-small cell lung cancer; p pathological stage; PET-CT positron emission tomography-computed tomography; PFs prognostic factors; RT radiotherapy; SR systematic review; SVCO superior vena caval obstruction; UICC Union for International Cancer Control.

**Table E2: Modifiable prognostic factors evaluated by reviews that investigated ‘any’ prognostic factor associated with survival**

| **Author, year** | **REV ID** | **Patient population** | **PF cat** | **PF code** | **Description of prognostic factor** | **No. of studies evaluated PF** | **Narrative synthesis** | **Significant studies** | **Level of significance*** |
| --- | --- | --- | --- | --- | --- | --- | --- | --- | --- |
| Ashworth, 2013 | 237 | NSCLC (oligometastatic) | T | Stage | Recursive partitioning analysis (RPA) classification 1 (vs 2) | 2 | + in 1 studies;  <> in 1 studies | 50% | Moderately significant |
|  |  |  | T | Surgery | lobectomy (vs pneumonectomy) | 2 | + in 1 studies;  <> in 1 studies | 50% | Moderately significant |
|  |  |  | T | Stage | Stage I (vs III or IV) | 5 | + in 1 studies;  <> in 4 studies | 25% | Occasionally significant |
|  |  |  | T | T-stage | Primary tumour stage | 7 | <> in 7 studies | 0% | Occasionally significant |
| Brundage, 2002 | 1051 | NSCLC (early; resected) | P | Smoking status | Smoking habit | 23 | + in 2 studies;  <> in 21 studies | 9% | NA |
|  |  |  | P | PS | Performance status | 13 | + in 4 studies;  <> in 9 studies | 31% | NA |
|  |  |  | P | Wt loss | Weight loss | 6 | <> in 6 studies | 0% | NA |
|  |  |  | T | Stage | “Stage” | 103 | + in 68 studies;  <> in 35 studies | 66% | NA |
|  |  |  | O | Surgery | Surgical procedure | 15 | + in 4 studies;  <> in 11 studies | 27% | NA |
| Berghmans, 2011 | 8434 | NSCLC (III) | P | Wt loss | weight loss | NS | + in 5 studies | NS | NA |
|  |  |  | P | QoL | Quality of life | NS | + in 1 study | NS | NA |
|  |  |  | P | PS | performance status | NS | + in 13 studies | NS | NA |
|  |  |  | C | BMI | Body mass index | NS | + in 1 study | NS | NA |
|  |  |  | P | Smoking | Smoking | NS | + in 1 study | NS | NA |
|  |  |  | T | Stage | Stage | NS | + in 6 studies | NS | NA |
|  |  |  | T | T volume | Tumour volume | NS | + in 2 studies | NS | NA |
|  |  |  | O | surgery | R0 (complete resection) | NS | + in 4 studies | NS | NA |

**Ashworth, 2013 [237]:** Only included prognostic factors assessed by MVA in at least 2 studies. Factors were categorised as being ‘highly significant’ if assessed by >2 studies and were significant in ≥50% of studies; ‘moderately significant’ if assessed in only 2 studies and found to be significant in ≥50% of studies; and ‘occasionally significant’ if assessed by >2 studies and found to be positive in <50% of studies. Actual comparison made (description of prognostic factor) only reported for significant studies.

**Berghmans, 2011 [8434]:** Only studies reporting MVA were included. A brief list of factors found to be significant by included study was provided, but in a very abbreviated format and most studies reported multiple significant factors. The number and actual factors evaluated by included studies was not stated. The review did not report the evidence underpinning individual factors, i.e. it did not the number of studies evaluating each factor, or found it to be significant.

**Brundage, 2002 [1051]:** Included separate analysis for studies that investigated prognostic factors in resected NSCLC. Limited to studies that report at least one multivariate analysis. The description of the prognostic factors were very brief and are reported verbatim here (not stated what the comparator was).

**Prognostic factor category (PF Cat):**

**T** Tumour characteristics

**P** Patient characteristics

**C** Clinical characteristics or routinely assessed biological variables

**O** Prognostic factors classified as ‘other’

**Abbreviations:** cat category; MVA multivariate analysis; NA not applicable; NS not stated; NSCLC non-small cell lung cancer; PF prognostic factor; REV ID review unique identifier number.
