# Supplementary material for: Pragmatic methods for reviewing exceptionally large bodies of evidence: systematic mapping review and overview of systematic reviews using lung cancer survival as an exemplar
Source: Syst Rev. 2019 Jul 16;8:171. doi: 10.1186/s13643-019-1087-4 (PMC6631880; doi:10.1186/s13643-019-1087-4)
Supplement: Supplementary file 6 — Appendix F. List of modifiable prognostic factors. Table F1: Modifiable prognostic factors evaluated by included reviews. (DOCX 14 kb) [file 13643_2019_1087_MOESM6_ESM.docx]

**APPENDIX F: LIST OF MODIFIABLE PROGNOSTIC FACTORS**

**Table F1: Modifiable prognostic factors identified by public health and clinical stakeholders**

(†*modifiable factors that were evaluated by studies included in the overview of reviews*)

| **PF CODE** | **DESCRIPTION** | **MODIFIABLE (CG)** | **MODIFIABLE**  **(DWH)** |
| --- | --- | --- | --- |
| ***Clinical characteristics or routinely assessed biological variables*** | | | |
| anaemia |  | yes |  |
| cachexia | Includes anorexia-cachexia syndrome, and cancer cachexia | yes |  |
| dyspnoea | Shortness of breath or breathlessness, is a subjective sensation of breathing discomfort. | yes |  |
| pain |  | yes |  |
| sarcopenia | Loss of skeletal muscle mass quality and strength | yes |  |
| TB | Tuberculosis | † | yes† |
| VitD level | Serum vitamin D level | **yes**† | **yes**† |
| WBC | White blood cell count |  | yes |
| ***Healthcare provider and system*** | | | |
| insurance status |  | **yes**† | **yes**† |
| MDT | Multidisciplinary team | **yes**† | **yes**† |
| procedural volume | Surgical procedure volume | yes† | † |
| surgeon |  | **yes**† | **yes**† |
| timeliness of care |  | **yes**† | **yes**† |
| ***Tumour characteristics*** | | | |
| GTV | Gross tumour volume GTV identified via three-dimensional conformal radiotherapy (3D-CRT) | † | Yes† |
| isolated mets | Isolated metastases. No other metastases |  | yes |
| Stage | Includes TNM tumour stage for NSCLC; differentiation between adenocarcinoma in situ (AIS) and minimally invasive adenocarcinoma (MIA) for adenocarcinoma (ADC); and limited vs extensive small cell lung cancer (SCLC). | † | yes† |
| ***Patient characteristics*** |  |  |  |
| BMI / *Wt loss*** | Body mass index or *Body weight loss* | **yes**† | ***yes***† |
| marital status |  | **yes** | **yes** |
| PS | Performance status | **yes/no**† | **yes**† |
| psychosocial factors | Stress-related psychosocial factors | **yes/no** | **yes** |
| QoL | Quality of life | yes/no† | † |
| Smoking status |  | **yes**† | **yes**† |
| ***Socioeconomic and sociodemographic factors*** | | | |
| SES | Socioeconomic status |  | yes |
| ***Other*** | | | |
| Occupation - painter |  | **yes** | **yes** |
| Surgical treatment | Received surgical treatment | **yes**† | **yes**† |
| surgery | Type of surgery received | **yes**† | **yes**† |
| vomiting |  | **yes** | **yes** |

*Based on post hoc decision

**Weight loss was originally categorised as a separate prognostic factor under ‘clinical characteristics or routinely assessed biological variables’, and BMI here under ‘patient characteristics’, but both factors are grouped together here as they were considered as a single prognostic factor in one review.
