# Supplementary material for: Pragmatic methods for reviewing exceptionally large bodies of evidence: systematic mapping review and overview of systematic reviews using lung cancer survival as an exemplar
Source: Syst Rev. 2019 Jul 16;8:171. doi: 10.1186/s13643-019-1087-4 (PMC6631880; doi:10.1186/s13643-019-1087-4)
Supplement: Supplementary file 7 — Appendix G. Quality appraisal of reviews investigating modifiable factors using a modified AMSTAR checklist. AMSTAR questions. Table G1. Quality appraisal of included reviews assessing modifiable factors. (DOCX 18 kb) [file 13643_2019_1087_MOESM7_ESM.docx]

**APPENDIX G: QUALITY APPRAISAL OF REVIEWS INVESTIGATING MODIFIABLE FACTORS USING A MODIFIED AMSTAR CHECKLIST**

**AMSTAR questions**

These are based on the checklist provided on: <http://amstar.ca/Amstar_Checklist.php> (Accessed July 2015) and published by *Shea et al. (BMC Medical Research Methodology 2007 7:10 doi:10.1186/1471-2288-7-10)*

1. Were there pre-defined eligibility criteria for relevant studies reported?

2. Was there duplicate study selection and data extraction?

3. Was a comprehensive literature search performed?

4 (a). Were grey literature or unpublished literature included]

4 (b). Did the review include publications not published in the English language?

5. Was a list of included or excluded studies, or PRISMA flow diagram provided?

6 (a). Was there systematic presentation of the characteristics of included studies provided?

*These should include known prognostic factors, such as age, sex, performance status, stage, and histology.*

6 (b). Was there systematic presentation of the findings of included studies?

7. Was the scientific quality of the included studies assessed and documented?

8. Was the scientific quality of the included studies used appropriately in formulating conclusions?

9 (a). Were meta-analysis conducted?

9 (b). Was the presence of heterogeneity assessed?

9 (c). Was significant heterogeneity identified?

9 (d). Was a random-effects approach used to pool the data?

9 (e). Did the meta-analysis include adjusted effect estimates?

10 (a). Was the likelihood of publication bias assessed?

10 (b). Was potential publication bias identified?

Each item was recorded as: yes (Y), no (N), partial (P), unable to answer (not stated) (U), or not applicable (NA).

**Table G1: Quality appraisal of included reviews assessing modifiable factors**

| **Author, Year** | **REV ID** | **A1** | **A2** | **A3** | **A4** | **A4b** | **A5** | **A6a** | **A6b** | **A7** | **A8** | **A9a** | **A9b** | **A9c** | **A9d** | **A9e** | **A10a** | **A10b** | **Quality assessment tool used** | **Overall Q** |
| --- | --- | --- | --- | --- | --- | --- | --- | --- | --- | --- | --- | --- | --- | --- | --- | --- | --- | --- | --- | --- |
| Aboshi, 2014 | 182 | Y | P | N | N | N | Y | Y | Y | N | N | Y | N | NA | N | Y | N | NA |  | Moderate |
| Ashworth, 2013 | 237 | Y | Y | Y | N | Y | Y | N | P | N | N | N | Y | Y | NA | Y | N | NA |  | Moderate |
| Ashworth, 2014 | 105 | Y | Y | Y | N | Y | N | P | Y | N | N | Y | Y | Y | Y | Y | N | NA |  | Moderate |
| Behera, 2016 | 5815 | Y | P | Y | N | U | Y | Y | N | N | N | Y | N | NA | N | N | N | NA |  | Moderate |
| Berghmans, 2011 | 8434 | Y | N | N | N | Y | N | P | N | N | N | N | Y | Y | NA | NA | N | NA |  | Poor |
| Brundage, 2002 | 1051 | N | N | N | N | N | N | N | N | N | N | N | Y | Y | NA | Y | N | NA |  | Very poor |
| Buttigliero, 2011 | 494 | Y | Y | Y | P | N | P | Y | Y | Y | N | N | Y | Y | NA | Y | N | NA | Newcastle-Ottawa scale | Good |
| Carter, 2014 | 5362 | Y | P | Y | Y | Y | Y | N | P | Y | Y | N | Y | Y | NA | Y | N | NA |  | Moderate |
| Christopoulos, 2013 | 5789 | Y | P | Y | N | U | Y | N | N | N | N | N | N | NA | NA | NA | N | NA |  | Very poor |
| Deghaidy 2005 | 923 | Y | N | N | N | N | N | N | Y | N | N | Y | Y | Y | Y | NS | N | NA |  | Very poor |
| Florou, 2014 | 51 | Y | Y | Y | N | N | N | N | N | N | N | N | N | NA | NA | NA | N | N |  | Poor |
| Montazeri, 2009 | 703 | Y | N | Y | N | N | Y | N | Y | N | N | NA | N | NA | NA | NA | N | NA | NS | Poor |
| Neal, 2015 | 8441 | Y | Y | Y | N | N | Y | P | N | Y | N | N | Y | Y | NA | U | N | NA | Checklist developed based on checklists by Altman 2001, Harden, 2013, and Crawford, 2002 (waiting time paradox) | Moderate |
| Olsson 2009 | 722 | Y | P | N | N | N | Y | N | N | Y | N | N | Y | Y | NA | P | N | NA | NS | Poor |
| Parsons, 2010 | 695 | Y | Y | Y | N | Y | Y | N | Y | Y | N | Y | Y | N | Y | Y | N | NA | Checklist developed by Altman (2001) for PFs | Good |
| Prades, 2015 | 5807 | Y | P | N | N | N | N | N | N | N | N | N | N | NA | NA | NA | N | NA |  | Very poor |
| Salah, 2012 | 467 | P | N | P | N | N | N | Y | Y | N | Y | NA | NA | NA | NA | Y | N | N | NS | Poor |
| Slatore, 2010 | 621 | Y | P | Y | P | NA | Y | Y | Y | Y | P | N | NA | NA | NA | NA | N | NA | A 16-item inventory developed for the review. | Moderate |
| von Meyenfeldt, 2012 | 414 | Y | Y | Y | N | N | Y | P | Y | N | N | Y | Y | Y | Y | Y | N | U | The authors noted that each study was quality appraised, but did not report how this was done or list the criteria used. However, they noted that studies were checked for selection bias, and when multiple use of the same database was established, the study with the largest patient cohort was used | Moderate |
| Yu, 2015 | 5489 | Y | N | Y | N | Y | Y | N | N | Y | N | Y | Y | Y | Y | U | Y | N | Newcastle Ottawa Scale | Poor |
| ***Total number of reviews that passed criterion:*** | | 18 | 7 | 13 | 1 | 6 | 12 | 5 | 9 | 7 | 2 | 7 | 12 | 11 | 5 | 9 | 1 | 0 |  |  |

**Abbreviations:** AMSTAR question; REV ID review unique identifier number; N no; NA not applicable; NS not stated; P partial; PFs prognostic factors; Q quality; Y yes
