# Supplementary material for: Pragmatic methods for reviewing exceptionally large bodies of evidence: systematic mapping review and overview of systematic reviews using lung cancer survival as an exemplar
Source: Syst Rev. 2019 Jul 16;8:171. doi: 10.1186/s13643-019-1087-4 (PMC6631880; doi:10.1186/s13643-019-1087-4)
Supplement: Supplementary file 8 — Appendix H. Summary of magnitude of effect for modifiable factors. Table H1. Summary effect estimates based on pooled data. Table H2. Summary effect estimates from individual studies included in reviews reporting narrative synthesis. (DOCX 23 kb) [file 13643_2019_1087_MOESM8_ESM.docx]

**APPENDIX H: SUMMARY OF MAGNITUDE OF EFFECT FOR MODIFIABLE FACTORS**

**Table H1: Summary effect estimates based on pooled data**

| **REVIEW: Author, year** | **IPD**  **REV** | **MODIFIABLE FACTORS CODE** | **DESCRIPTION OF MODIFIABLE FACTOR** | **POPULATION** | **RESULTS: Unadjusted analyses** | **RESULTS: Adjusted analyses** |
| --- | --- | --- | --- | --- | --- | --- |
| **Parsons, 2010 [695]*** | N | Smoking cessation after diagnosis | Smoking cessation after diagnosis (vs non) | NSCLC  (predominantly early stage, I-IIIA) | Unadjusted HR for all-cause mortality (4 studies):  1.19, 95% CI: 0.91 to 1.54 (I^2^=0.0%).  [*no studies reported impact on cancer specific mortality*] | Adjusted HR for all-cause mortality (1 study):  2.94, 95% CI: 1.15 to 7.54;  *adjusted for age, gender, type of operation, histology, prospective RT, N status, T status, and previous malignancies*.  [no studies reported impact on cancer specific mortality] |
| **Parsons, 2010 [695]*** | N | Smoking cessation after diagnosis | Smoking cessation after diagnosis (vs non) | SCLC  (predominantly limited stage) | Unadjusted HR for all-cause mortality (2 studies):  1.18, 95% CI: 1.03 to 1.36 (I^2^=0.0%)  [*no studies reported impact on cancer specific mortality*] | Adjusted HR for all-cause mortality ():  1.86, 95% CI: 1.33 to 2.59;  *adjusted for sex, age, and volume of limited disease*  [no studies reported impact on cancer specific mortality] |
| **Behera, 2016 [5815]** | N | Stage | ADC in situ, AIS) (vs minimally invasive ADC, MIS) | NSCLC (I; ADC) | ONLY LIMITED DATA PRESENTED  [*Pooled OS rates across all ADC, and then separately for AIS and MIA*]  5-yr survival rate:  AIS (7 studies, 215 pts): 100%;  MIA (8 studies, 136 pts): 98.5%  (*no significant difference found between the two groups; t-test*)  Entire group (11 studies, 547 pts): 97.5%, 95% CI: 0.96 to 0.99 [*95% CI ONLY reported for the pooled data across the entire*] |  |
| **Deghaidy 2005 [923]** | N | Stage | Stage I (vs II) | LC (NSCLC) | Risk of death at 5-yrs (10 studies, 4557 pts) [RR<1 corresponds to reduced risk of death]:  RR: 0.78, 95% CI: 0.7 to 0.87 |  |
| **Deghaidy 2005 [923]** | N | Stage | Stage I (vs II) | LC (SCLC) | Risk of death at 5yrs (4 studies, 502 pts):  RR: 0.84, 95% CI: 0.77 to 0.91 |  |
| **Deghaidy 2005 [923]** | N | Stage | Stage I (vs III) | LC (NSCLC) | Risk of death at 5yrs (7 studies, 2895 pts):  RR: 0.67, 95% CI: 0.56 to 0.79 |  |
| **Deghaidy 2005 [923]** | N | Stage | Stage I (vs III) | LC (SCLC) | Risk of death at 5yrs (3 studies, 211 pts):  RR: 0.77, 95% CI: 0.66 to 0.87 |  |
| **Deghaidy 2005 [923]** | N | Stage | stage II (vs III) | LC (NSCLC) | Risk of death at 5yrs (7 studies, 21927 pts):  RR: 0.81, 95% CI: 0.72 to 0.91 |  |
| **Deghaidy 2005 [923]** | N | Stage | stage II (vs III) | LC (SCLC) | Risk of death at 5yrs (3 studies, 170 pts):  RR: 0.93, 95% CI: 0.84 to 1.04 |  |
| **Deghaidy 2005 [923]** | N | Stage | stage III (vs IV) | LC (NSCLC) | Risk of death at 5yrs (6 studies, 2910 pts):  RR: 0.84, 95% CI: 0.75 to 0.93 |  |
| **Deghaidy 2005 [923]** | N | Stage | stage III (vs IV) | LC (SCLC) | Risk of death at 5yrs (2 studies, 341 pts):  RR: 0.99, 95% CI: 0.94 to 1.06 |  |
| **Aboshi, 2014 [182]** | N | PS | Percentage of patients in study with ECOG PS1 <60 (vs ≥60) | NSLC (late) | Unadjusted OR (13 studies, 2419 pts): 1.49, 95% CI: 0.54 to 4.10 |  |
| **Aboshi, 2014 [182]** | N | Stage | Percentage of patients in study with stage IV disease <80 (vs ≥80) | NSLC (late) | Unadjusted OR (13 studies, 2419 pts): 0.24, 95% CI: 0.05 to 1.17 | Adjusted OR (13 studies, 2419 pts):  0.42, 95% CI: 0.16 to 1.18 |
| **Ashworth, 2014 [105]** | Y | Stage | IB (vs IA) | NSCLC (oligometastatic; received curative treatment of primary lung cancer) | Unadjusted HR [*risk of death*] (training set, 505 pts)  1.16, 95% CI: 0.78 to 1.72 |  |
| **Ashworth, 2014 [105]** | Y | Stage | IIA (vs IA) | NSCLC (oligometastatic; received curative treatment of primary lung cancer) | Unadjusted HR [*risk of death*] (training set, 505 pts)  1.55, 95% CI: 0.70 to 3.45 |  |
| **Ashworth, 2014 [105]** | Y | Stage | IIB (vs IA) | NSCLC (oligometastatic; received curative treatment of primary lung cancer) | Unadjusted HR [*risk of death*] (training set, 505 pts)  1.53, 95% CI: 1.03 to 2.26 |  |
| **Ashworth, 2014 [105]** | Y | Stage | IIIA (vs IA) | NSCLC (oligometastatic; received curative treatment of primary lung cancer) | Unadjusted HR [*risk of death*] (training set, 505 pts)  1.99, 95% CI: 1.37 to 2.88 |  |
| **Ashworth, 2014 [105]** | Y | Stage | IIIB (vs IA) | NSCLC (oligometastatic; received curative treatment of primary lung cancer) | Unadjusted HR (training set, 505 pts)  1.91, 95% CI: 1.14 to 3.20 |  |
| **Ashworth, 2014 [105]** | Y | Surgical treatment | Surgical treatment for primary LC (vs non-surgical) | NSCLC (oligometastatic; received curative treatment of primary lung cancer) | Unadjusted HR (training set, 505 pts)  0.74, 95% CI: 0.55 to 1.00 [p=0.48] |  |
| **Salah, 2012 [467]** | Y*** | Stage | Intra-thoracic **stage III** (vs stage II or I) of the primary tumour | NSCLC (solitary metastases in sites other than brain or adrenal gland; and undergone resection for metastasis) | Significant in UVA  Individual patient data (n=62) from published studies used to develop Kaplan Meir curves.  5-yr survival rate: 0% (III) vs 77% (II) vs 63% (I);  Mean survival: 23 mths (95% CI: 12 to 33) vs 79 mths (95% CI: 58 to 100) vs 77 mths (95% CI: 62 to 92);  p<0.001 Log Rank test | Not significant in multivariate regression analysis (results not reported) |
| **von Meyenfeldt, 2012 [414]**** | N | procedural volume | High annual hospital volume of surgical resections (vs low volume) | LC |  | Adjusted OR for post-operative mortality (10 studies):  0.71, 95% CI: 0.62 to 0.81 (I^2^=53%);  *adjusted for age, gender, and other parameters*.  Outcome ‘post-operative mortality = 30-day or in-hospital mortality. 1 study excluded from MA as it did not report OR. *Significant inverse relationship between hospital volume and mortality found in 5/11 studies*.  Regression analysis did not identify a cut-off value for volume of resections for lung cancer to differentiate hospitals with high vs low post-operative mortality. |
| **von Meyenfeldt, 2012 [414]**** | N | procedural volume | High annual hospital volume of surgical resections (vs low volume) | LC |  | Adjusted HR [*for risk of death*] for overall survival (7 studies):  0.93, 95% CI: 0.84 to 1.03 (I^2^=85%);  *adjusted for age, gender, and other parameters*.  Subgroup analysis – adjusted for comorbidities (4 studies):  0.88, 95% CI: 0.81 to 0.95  1 study excluded from MA as it did not report HR. 5/8 studies showed significant inverse relationship between hospital volume and survival, and one showed a significant benefit for the lower-volume category. |
| **von Meyenfeldt, 2012 [414]** | N | procedural volume | High annual surgeon procedural volume of surgical resections (vs low volume) | LC |  | Adjusted OR for post-operative mortality (2 studies):  0.68, 95% CI: 0.42 to 1.08 (I^2^=66%);  *adjusted for age, gender, comorbidities, +/- other parameters.* |
| **von Meyenfeldt, 2012 [414]** | N | Surgeon | Surgeon specialty: general thoracic surgeon (vs general surgeon) | LC |  | Adjusted OR for post-operative mortality of lung resections for cancer (3 studies):  0.78, 95% CI: 0.70 to 0.88 (I^2^=NS);  *adjusted for age, gender, comorbidities, treatment*, *+/- other parameters*. |
| **von Meyenfeldt, 2012 [414]** | N | Surgeon | Surgeon specialty: general thoracic surgeon (vs general surgeon) | LC |  | Adjusted HR [*for risk of death*] for survival (2 studies):  0.86, 95% CI: 0.74 to 1.00 (I^2^=28%);  adjusted for age, gender, tumour characteristics, +/- other parameters. |
| **von Meyenfeldt, 2012 [414]** | N | Surgeon | Surgeon specialty: cardiothoracic surgeon (vs general surgeon) | LC |  | Adjusted OR for post-operative mortality of lung resections for cancer (3 studies):  0.82, 95% CI: 0.69 to 0.96 (I^2^=NS);  *adjusted for age, gender, comorbidities, treatment*, *+/- other parameters*. |
| **Yu, 2015 [5489]** | N | GTV | Small gross tumour volume GTV < 112 cm3vs (vs large GTV >= 112cm3) | NSCLC (unresectable) | HR for overall survival (5 studies, 4172 pts):  1.52, 95% CI: 1.10 to 1.94  HR for 1-yr survival (5 studies, 1058 pts):  1.27, 95% CI: 1.10 to 1.46  HR for 3-yr survival (3 studies, 855 pts):  2.06, 95% CI: 1.63 to 2.61  HR for 5-yr survival (2 studies, 682 pts):  2.25, 95% CI: 1.63 to 3.10  All pts were treated with RT |  |

OR / RR /HR <1 corresponds to a reduced risk in the group with the prognostic factor, and an OR / RR /HR >1 corresponds to an increased risk (NB some studies reported the risk of survival whilst others the risk of death)

***Parsons, 2010 [695]:** Life table modelling indicated that most of the benefit from smoking cessation is due to a reduced likelihood of cancer progression rather than a reduction in cardiorespiratory deaths

****von Meyenfeldt, 2012 [414]:** Reported two outcome measures, post-operative mortality and longer term survival. The HR for the latter appears to relate to risk of death. All the forest plots indicated that the results falling on the left hand side favoured the ‘high volume group’, and the direction of the effects were interrupted in the same way for both the outcomes, post-operative mortality and survival.

*****Salah, 2012 [467]:** Evaluation based on data from published papers, with meta-regression analyses conducted on all participants treated as a single cohort (51 articles included 75 pts; 13 pts were excluded and 62 included in the analysis). Variables found to be approaching significant or potentially significant in univariate analysis were included in the multivariate analysis (Cox Proportional hazard regression model).

**Ashworth, 2014 [105]:** Factors predictive of overall survival were evaluated using Cox regression based on individual patient data. Analyses were conducted on training and validating sets (two-thirds and one-third of patients, respectively), developed using a nonstratified random number generator. Any variables associated with OS or PFS on univariable analysis (P < .05) were considered for entry into a forward-stepwise multivariable model with entry and removal at the 10% level of significance (ie, P <0.10).

**Abbreviations:** CI confidence interval; HR hazard ratio; LC lung cancer; mths months; MVA multivariate analysis; NSCLC non-small cell lung cancer; NS not stated; OR odds ratio; OS overall survival; pts patients; RR relative risk; surR surgical resection; syn synchronous; UVA univariate analysis

**Table H2: summary effect estimates from individual studies included in reviews reporting narrative synthesis**

| **REVIEW: AUTHOR, YEAR [ID]** | **MODIFIABLE FACTOR** | **MODIFIABLE FACTOR DESCRIPTION** | **POPULATION** | **RESULTS** |  |
| --- | --- | --- | --- | --- | --- |
| **Prades, 2015 [5807]** | MDT | MDT patient management | mxdC LC (NSCLC) | Included any cancer. 3/29 studies that assessed impact of MDT patient management on outcomes included LC. Only 1 study evaluated impact on survival (and treatment), which was a prospective study of hospital cancer registry database.  1–year survival: 33% among pts discussed at MDT vs 18% in pts not discussed in MDT (p=0.05, statistical test NS) | **+** |
| **Christopoulos, 2013 [5789]** | TB | Active tuberculosis, TB (vs no TB) | LC | No data were reported on histological subgroups.  Median survival reported for 5 studies, but only one study included pts with and without TB; the remaining 4 studies only included data for LC patients with concurrent TB.  Median survival:  pts with active TB 4 months (range 3 to 5 months; 5 studies)  pts without TM 8 months (1 study)  The mean mortality rate associated with TB was 22.6% (SD +/- 14.4%) (11 studies) | **?** |
| **Slatore, 2010 [621]** | Insurance status |  | LC | The study included 9 studies that evaluated the effect of insurance coverage on LC survival (any type). Only the findings of three studies limited to NSCLC are reported here.  Study also investigated association between insurance status and LC incidence rates, stage at diagnosis, access to care, and delivery of care. |  |
|  |  | Commercial or other insurance (vs private insurance) | (NSCLC; any / I-II) | Any stage: Adjusted OR for mortality (1 study, 1403 pts):  1.04, 95% CI: 0.93 to 1.76  Stage I-III: Adjusted HR for mortality (1 study, 336 pts):  1.10, p=0.48 | **<>** |
|  |  | Medicaid/Medicare (vs Medicare) | (NSCLC I-III) | Adjusted HR for mortality (1 study, 3094 pts):  (all-cause) if resected: 1.42, 95% CI: 1.09 to 1.87  (all-cause) if no resection: 1.17, 95% CI: 0.96 to 1.73  Perioperative: 1.67, 95% CI: 0.54 to 5.17 | **+**  **<>**  **<>** |
| **Florou, 2014 [51]** | Smoking status |  | mxdC LC | Included any cancer. The study evaluated the effects of smoking on survival, treatment effects, a second primary tumour and quality of life. 8/20 included studies evaluated effect of smoking on survival. This included 4 LC studies (2 NSCLC and 2 SCLC). |  |
|  |  | ex-smoker - quit smoking before diagnosis (vs current smoker or never smoked) | (NSCLC; 1A-IIB) | Smoking cessation associated with non-significant better OS; p=0.9, statistical test NS. (1 study n=543; investigated whether smoking cessation after diagnosis affects survival, post-surgical resection).  The same study also found:  Significant benefit of quitting found in women (subgroup-analysis).  Length of survival was positively correlated with duration of smoking abstinence (no results presented). | **<>** |
|  |  | *never smoked (vs ex- or current smoker)* | (NSCLC; curative surgery) | ‘Smoking status before surgery (ex-smokers and current smokers) and after surgery (ex-smokers, smokers who quit, and smokers who continued) were found to be significantly associated with survival’. (1 study n=238; investigated the effect of multiple factors, no results presented). | **+** |
|  |  | non-smoker (vs smoker) | (SCLC; limited) | Significant difference in actual OS favouring non-smoking cohort (1 study n=215; investigated the effect of multiple factors, results NS) | **+** |
|  |  | ex-smoker - quit smoking at or after diagnosis (vs current smoker) | (SCLC; limited) | Pts who quit smoking at or after diagnosis had a reduced risk of death compared to smokers (*those who never quit smoking*). Adjusted risk of death was reduced by 45%; no further data reported. (1 study n=284; investigated the effect of multiple factors). | **+** |
|  |  | never smoked (vs ex- or current smoker) | (SCLC; limited) | Neither smoking status at time of diagnosis (ex-smokers or current smokers) nor intensity (pack years smoked) had a significant impact on survival (1 study n=284; investigated the effect of multiple factors; results NS). | **<>** |
| **Montazeri, 2009 [703]** | QoL |  | mxdC LC | *Included any cancer, 26 studies included in a separate table for LC. The findings reported here only relate to 12 studies of NSCLC or SCLC. Not considered here are 14 studies: 8 of lung cancer only (or lung cancer plus other solid tumour type), 2 studies that reported combined data for SCLC and NSCLC, 2 studies of pleural* mesothelioma, 1 of recurrent malignant pleural effusion, and 1 of bronchogenic carcinoma. |  |
|  |  | FACT-L (TOI) | (NSCLC) | Change in TOI score not associated with survival. A trend was noted for shorter survival with the longest negative change in TOI score. (1 study, 30 pts) | **<>** |
|  |  | EORTC QLC-C30 +EORTC QLQ-LC17 + DDC | (NSCLC) | Global QoL role functioning, fatigue, appetite, and constipation were prognostic indicators for survival. (1 study, 273 pts) | **+** |
|  |  | LCCS | (NSCLC) | Pre-treatment LCSS global symptom score was independent determinant of OS. (1 study, 301 pts) | **+** |
|  |  | EORTC QLC-C30 | (NSCLC; III) | Appetite loss was the most significant prognostic factor of survival. (1 study, 301 pts) | **+** |
|  |  | EORTC QLC-C30 + Duke-UNC Social Support Scale | (NSCLC; late) | Pain was a significant predictor of OS, but overall QoL was not (1 study, 206 pts) | **+** |
|  |  | Total FACT-L score | (NSCLC; late) | Total FACT-L score was a predictor of survival. (1 study, 222 pts) | **+** |
|  |  | FACT-L +TOI | (NSCLC; late) | Baseline physical wellbeing and TOI scores predict survival. (1 study, 573 pts) | **+** |
|  |  | Pain, dysphagia | (NSCLC; late) | Pain, and dysphagia were significant prognostic factors of survival. (1 study, 391 pts) | **+** |
|  |  | ADL + IADL + EORTC QLC-C30 (global QoL) | (NSCLC; late) | Global QoL and IADL were significant prognostic factors for OS. (1 study, 566 pts) | **+** |
|  |  | Psychological well-being and disease related symptoms + personal functioning + everyday activity | (NSCLC; inoperable) | General symptoms and psychological well-being were the best predictive value for survival. (1 study, 102 pts) | **+** |
|  |  | EORTC QLC-C30 | (NSCLC; inoperable) | Global QoL was a strong prognostic factor of survival. (1 study, 198 pts) | **+** |
|  |  | EORTC QLC-C30 + CES + MOS Social Support Questionnaire + sleep quality scale | (SCLC) | Higher depressive symptoms were borderline significant in predicting decreased survival. (1 study, 70 pts) | **<>** |

**Montazeri, 2009 [703]**: All results were obtained from multivariate analyses after controlling for one or more demographic and known biomedical prognostic factors.

**Abbreviations**: ADL: Activities of Daily Living; CES-D: Centre for Epidemiologic Studies-Depression Scale; DDC: Daily Diary Card; EORTC QLQ-C30: European Organization for Research and Treatment of Cancer Quality of Life Core Questionnaire; EORTC QLQ-LC13 (or QLQ LC17): EORTC Lung Cancer specific Quality of Life Questionnaire (previously containing 17items); FACT-G: Functional Assessment of Cancer Therapy-General module; FACT-L: Functional Assessment of Cancer Therapy-Lung module; FLI-C: Functional Living Index-Cancer; IADL: Instrumental Activities of Daily Living; KPS: Karnofsky Performance Status; LC lung cancer; LCSS: Lung Cancer Symptoms Scale; MDT multidisciplinary team; MOS: Medical Outcomes Study; mxdC LC review included mixed cancers and reported separate data for lung cancer; NHP: Nottingham Health Profile; OS overall survival; Pts patients; QOL: quality of life; RT radiotherapy; SDS: Self-rating Depression Scale; TIQ: Therapy Impact Questionnaire; TOI: Trial Outcome Index.
